# Calcium-dependent protein kinases in cotton: insights into early plant responses to salt stress

**DOI:** 10.1186/s12870-018-1230-8

**Published:** 2018-01-17

**Authors:** Wei Gao, Fu-Chun Xu, Dan-Dan Guo, Jing-Ruo Zhao, Ji Liu, Ya-Wei Guo, Prashant Kumar Singh, Xiao-Nan Ma, Lu Long, Jose Ramon Botella, Chun-Peng Song

**Affiliations:** 1 State Key Laboratory of Cotton Biology; Henan Key Laboratory of Plant Stress Biology; School of Life Science, Henan University, Kaifeng, Henan 475004 People’s Republic of China; 2 State Key Laboratory of Cotton Biology, Institute of Cotton Research of CAAS, Anyang, 455000 People’s Republic of China; 3 School of Agriculture and Food Sciences, University of Queensland, Brisbane, QLD 4072 Australia

**Keywords:** Calcium dependent protein kinase, *Gossypium hirsutum*, Genome-wide identification, Transcriptome, Virus-induced gene silencing

## Abstract

**Background:**

Soil salinization is one of the major environmental constraints to plant growth and agricultural production worldwide. Signaling components involving calcium (Ca^2+^) and the downstream calcium-dependent protein kinases (CPKs) play key roles in the perception and transduction of stress signals. However, the study of CPKs in cotton and their functions in response to salt stress remain unexplored.

**Results:**

A total of 98 predicted CPKs were identified from upland cotton (*Gossypium hirsutum* L. ‘TM-1’), and phylogenetic analyses classified them into four groups. Gene family distribution studies have revealed the substantial impacts of the genome duplication events to the total number of *GhCPK*s. Transcriptome analyses showed a wide distribution of *CPK*s’ expression among different organs. A total of 19 *CPK*s were selected for their rapid responses to salt stress at the transcriptional level, most of which were also incduced by the thylene-releasing chemical ethephon, suggesting a partal overlap of the salinity and ethylene responses. Silencing of 4 of the 19 *CPKs* (*GhCPK8*, *GhCPK38*, *GhCPK54*, and *GhCPK55*) severely compromised the basal cotton resistance to salt stress.

**Conclusions:**

Our genome-wide expression analysis of *CPK* genes from up-land cotton suggests that *CPK*s are involved in multiple developmental responses as well as the response to different abiotic stresses. A cluster of the cotton *CPK*s was shown to participate in the early signaling events in cotton responses to salt stress. Our results provide significant insights on functional analysis of *CPK*s in cotton, especially in the context of cotton adaptions to salt stress.

**Electronic supplementary material:**

The online version of this article (doi: 10.1186/s12870-018-1230-8) contains supplementary material, which is available to authorized users.

## Background

Soil salinization is one of the major environmental constraints to crop production worldwide. The accumulation of salt in the arable soil causes detrimental changes in the morphology and physiology of plants, leading to dramatic reductions in crop quality and yield [1, 2]. To date, about one third of total croplands are threatened by one or more types of salinization [3]. The causes of high salt concentrations in soil are complex, including arid climates, high underground water levels, seawater infiltration, and so on. According to global climate change patterns and irrigation practices, the adverse effects of salt stress on crop production are expected to increase in the immediate future [1].

Plants have developed important defensive responses that enable their survival and reproduction in hostile environments, which can be activated within minutes to several weeks after exposure to a high salinity environment. The initial perception and response to salt stress is a crucial step for plants to switch to a resistent state in saline soil. In this step, the toxic ions (principally Na^+^ and Cl^−^) enter through the roots, causing increased water loss and turgor pressure. Additionally, a series of signaling networks involving rapid and long-distance signaling may be activated or depressed. Expanded studies have suggested the involvement of some signal events in the early stages of plant responses, including calcium (Ca^2+^) perception, reactive oxygen species production, and protein phosphorylation. Although intensive studies have been conducted on this subject, the mechanisms behind early plant responses to salt stress remain unclear.

Ca^2+^, which has been characterized as a conserved second messenger, plays essential roles in the signal perception and transduction in the plant which utilize Ca^2+^-dependent signal networks. The transient and minor changes of Ca^2+^ caused by extracellular stimuli can be sensed by Ca^2+^ sensors or Ca^2+^-binding proteins and lead to strong responses of downstream factors. Calcium-dependent protein kinases (CPKs) as the well-known Ca^2+^-sensor proteins, form one of the largest and most differentiated gene family in plants. CPK proteins are composed of four characterized domains: a highly variable N-terminal domain, a catalytic Ser/Thr kinase domain, an autoinhibitory region, and a conserved regulatory calmodulin-like domain (CaM-LD). The C-terminal CaM-LD, which binds Ca^2+^ with EF-hands structure, has enabled CPK functions in signaling perception and transduction coupled with plant responses involving Ca^2+^ [4, 5].

CPKs have been characterized from many plant species [5–10] and a recent review by Simeunovic et al. provides an overview of identified CPK targets [11]. CPK proteins exhibit very specific distribution among different tissues and organelles, which match their substantially differentiated functions [11]. CPKs have been reported to play important roles in the plant response to salt stress. For example, CDPK1 and CDPK1a can activate drought and high salt stress-inducible promoters in maize protoplasts, suggesting the involvement of CPKs in plant signaling responses to salt stresses [12]. *OsCPK7* and *OsCPK12* are two positive regulators salt tolerance in rice. *OsCPK7* is predominantly expressed in vascular bundles, where water stress is most severe when rice plants are subjected to salt and drought stresses. Transgenic rice overexpressing *OsCPK7* showed increased resistance to salt as well as enhanced induction of some stress-responsive genes, such as *rab16A* [13, 14]. OsCPK12 promotes salt stress responses through the up-regulation of ROS-scavenging enzymes (OsAPx2 and OsAPx8) and repression of stress-induced ROS overproduction. The expression of *OsCPK12* is closely related to salt tolerance in rice with mutations in *OsCPK12* and silencing by RNA interference resulting in reduced tolerance to salt stress [15]. AtCPK23 participates in responses to abiotic stress; *cpk23* mutants have shown greatly enhanced tolerance to drought and salt stresses, while seedlings overexpressing *AtCPK23* have displayed opposite phenotypes compared to mutants [16]. Several CPKs regulate ion channel activity [11], with Arabidopsis’ AtCPK3, 4, 5, 11 and 29 being able to phosphorylate AtTPK1 in vitro, a vacuolar K^+^ channel that controls K^+^ efflux and stomatal movement [17, 18]. Plant CPKs are also involved in the transcriptional response to important signaling molecules such as ABA, ROS and so on [11]. Despite the intensive work of CPKs participating in plant responses to salt stresses, their involvement in the early signaling events remains to be elucidated.

Cotton is a globally cultivated crop of significant agricultural and economic importance, as a major source of fiber and oil. The productivity and quality of cotton are adversely affected by hostile environments. The continual soil salinization process limits arable land. Therefore an alarming global demand for cotton requires the development of new cotton varieties with enhanced productivity in saline environments [19, 20]. Although, CPKs from various plants have been identified and their regulation mechanisms in plant development or responses to stress have been explored [21–26], little is known about the sensing and regulatory network of CPKs in cotton involved in response to salt stress. The only functionally characterized cotton CPK, GhCPK1, is reported to induce and play a role in calcium signaling in fiber development [27]. Recently, it has been reported that CPK11 of *Arabidopis thaliana* could phosphorylate drought-induced protein 19 (GhDi19–1 and GhDi19–2) of Cotton plant in vitro [28]. Unfortunately, the endogenous cotton CPK which activates these proteins is no yet studied. The complete genome of upland cotton (*Gossypium hirsutum* L.) cultivar ‘TM-1’ was sequenced and released, and this genome provides the necessary data for a genome-wide identification of the *CPK* gene family in allotetraploid cotton [29, 30].

In the present study, we first identified 98 putative CPK members from *G. hirsutum* by using the publicly available database in conjunction with bioinformatics, and we then analyzed their expression patterns among different organs as well as in response to salt stress and phytohormones treatments. The expression and clustering analysis under salt stress revealed an early accumulation of 19 *CPK* genes’ transcripts, which sharply increased within 1 h upon salt treatment. Thus, these *CPK* genes could be used as important indicators of salt stress in *G. hirsutum*. Our results provide both important insights into the evolution and function of cotton *CPKs* as well as candidate genes for breeding salt-resistant cotton varieties.

## Results and discussion

### Identification of CPKs in *G. hirsutum*

CPK proteins are evolutionarily conserved Ca^2+^ sensory proteins involved in the regulation of plant responses to a wide range of stimuli. The number of CPK members has substantially increased throughout the evolutionary process, resulting in the diversification of homologues with various functions across different species [31]. The putative CPKs were determined by searching the cotton genome database of the allotetraploid (AADD genome) upland cotton cultivar ‘TM-1’ with HMM (Hidden Markov Model) profiles constructed using *CPK* sequences from *Arabidopsis*, rice, maize, and grape as queries [5–7, 9]. All the putative CPKs were further subjected to Pfam and SMART analyses to identify their conserved domains and signature sequences. The candidates that possess both Ser/Thr kinase domain and CaM-LD were considered as cotton CPK proteins. Respectively, 41 and 43 CPKs were identified from the diploid cotton genomes of *G. raimondii* (DD genome) and *G. arboreum* (AA) [10]. After this screening method, a total of 98 putative CPKs were identified and named CPK1 to CPK98 based on their accession numbers (Additional file 1). The CPKs in *G. hirsutum* outnumbered the combined CPKs of *G. raimondii* and *G. arboretum*. Allotetraploid cotton originated from interspecific hybrids between species with A and D genomes, suggesting that the expansion of *CPK* family genes from diploid cotton to allotetraploid cotton is mainly a consequence of the tetraploidization of *G. hirsutum* during the evolution process. The 98 cotton *CPK* genes encode proteins that varied widely from 264 to 1191 amino acids in length, with predicted isoelectric points (*pI*) ranging from 4.92 to 9.28, and molecular weights (Mw) from 29.75 to 134.30 kD (Additional file 1). The variation in CPKs reflects their diverse cellular functions and is consistent with previous reports in other species [5–10].

### Phylogenetic analysis of cotton CPKs

**Fig. 1:**
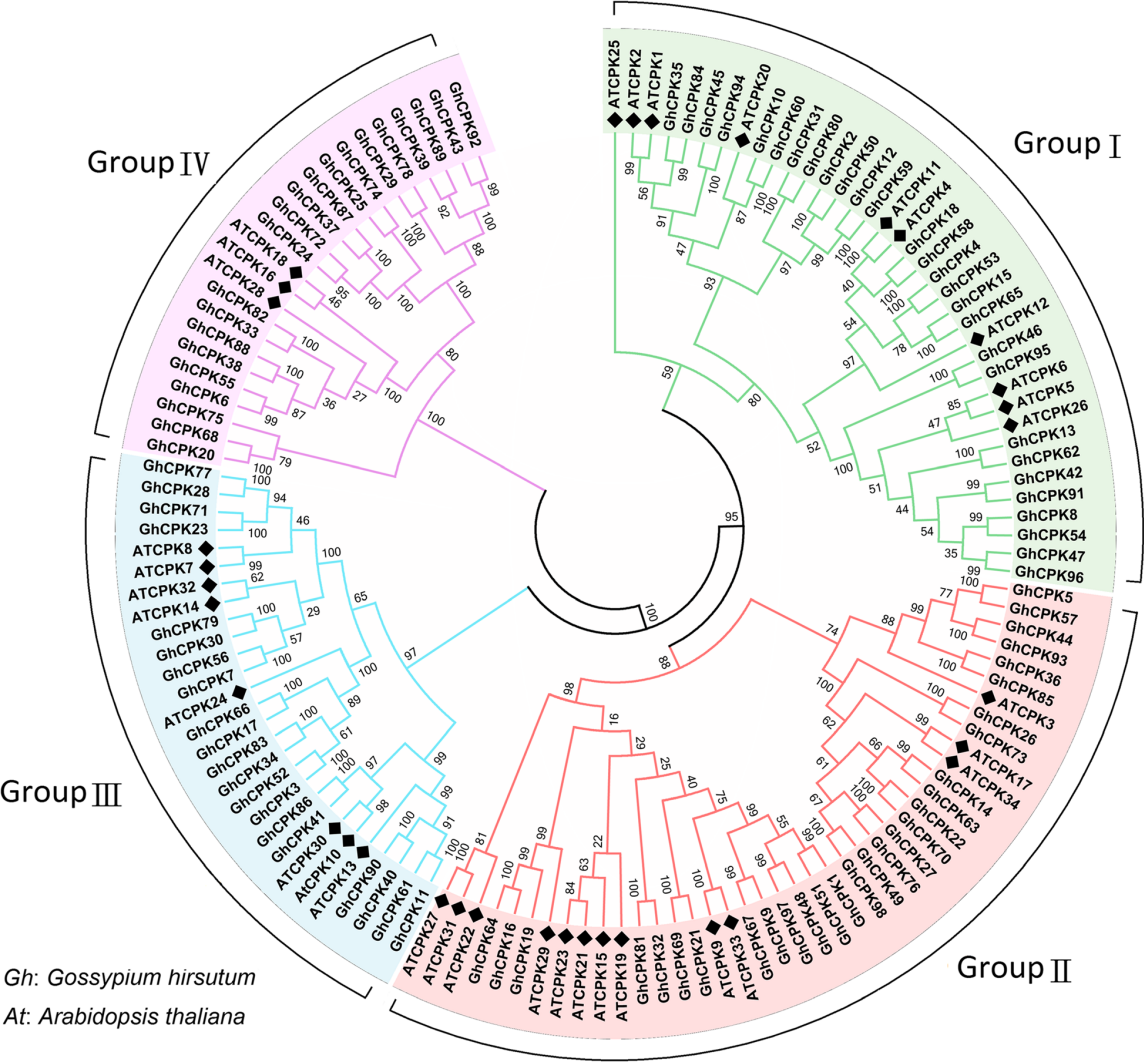
Phylogenetic analysis of CPK family members in cotton. The phylogenetic tree depicts 98 predicted cotton CPKs and 34 CPKs from *A. thaliana*, and the un-rooted tree was generated using MEGA6.0 with the neighbor-joining method (1000 bootstrap replicates). *Arabidopsis* CPKs are denoted with black rhombuses; the numbers represent bootstrap values. All CPKs were classified into one of four groups (groups I-IV); the distinct groups are shown by differently colored branches and backgrounds

MEGA6.0 was used to generate an un-rooted phylogenetic tree to categorize the CPKs from *G. hrisutum* and investigate their evolutionary relationships with homologues from *Arabidopsis* (Additional file 2). The 98 identified CPKs from cotton were divided into four groups based on sequence conservation (Fig. 1). The results of sequence alignment and phylogenetic analysis of cotton *CPK* genes revealed gene duplication as a common phenomenon in this family. For example, GhCPK21 and GhCPK69 shared an identity of 98.1% in their protein sequences (Additional file 3) and were predicted to have similar *pI* and Mw values (Additional file 1), with only a few single nucleotide polymorphism (SNP), which were observed in the non-functional region. GhCPK21 was located on chromosome A6 (accession number A06G1772) and GhCPK69 on chromosome D6 (accession number D06G2206). Thus, GhCPK21 and GhCPK69 are homologues from two different sub-genomes. Similarly, four highly homologous members (GhCPK14, GhCPK22, GhCPK63, and GhCPK70) shared more than 91.14% amino acid sequence identity (Additional file 4). These results indicated that polyploidization duplicated a considerable number of *CPK* genes in cotton. This may explain the large number CPKs in cotton relative to other plant species, i.e., the 34 CPKs in *Arabidopsis* [5], 31 in rice [6], and 31 in maize [7].

Among these four groups, group II was the largest group in both cotton and *Arabidopsis*, containing 29 cotton CPKs and more than one-third of the total *Arabidopsis* CPKs. Groups I, III, and IV included 28, 20, and 21 cotton CPKs, respectively. According to previous research, group IV is the smallest group and contained only 3 CPKs from *Arabidopsis* [5], 4 from rice [6], 3 from maize [7], and 3 from grape [9], respectively. Here we found that 21 cotton CPKs were categorized into group IV (Additional files 2 and 5). The above results suggested that in addition to the whole genome duplication, other evolutionary events were also contributed to the number of cotton CPKs.

### Structural divergence of *CPK* genes in *G. hirsutum*

**Fig. 2:**
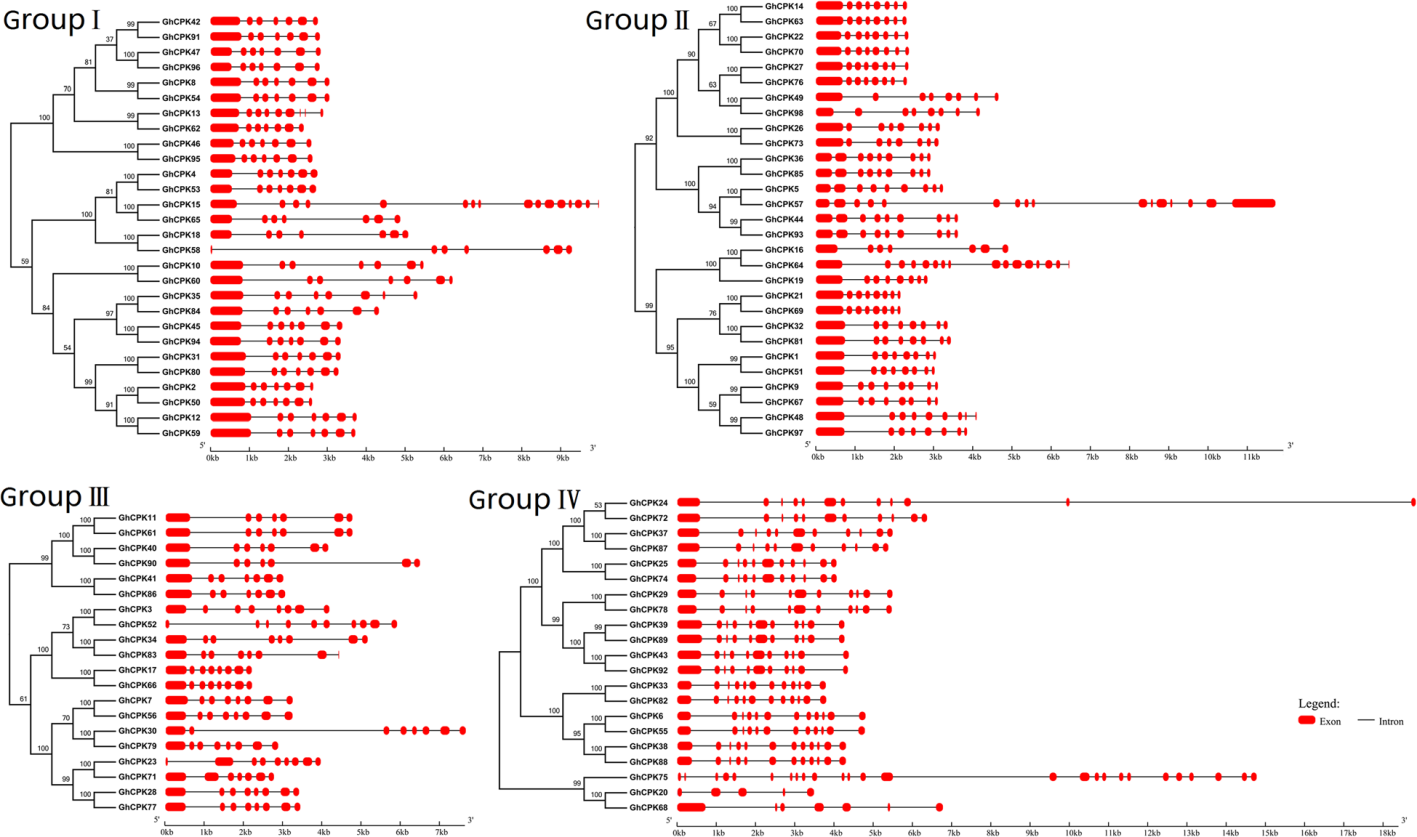
Phylogenetic relationships and exon-intron organization of *G. hirsutum CPK* genes (*GhCPKs*). The phylogenetic analysis of *G. hirsutum* CPK members (left) and the schematic for *CPK* intron-exon structures (right) of the four different groups. The phylogenetic tree was built using MEGA6.0 with the neighbor-joining method (1000 bootstrap replicates). Gene structures were inferred using GSDS 2.0 and are proportionally displayed according to the scale shown; the black lines indicate introns, while the red boxes represent exons

The structural analysis of *CPK* genes revealed that all the identified *CPK*s have several introns (Fig. 2). *CPK*s within the same group shared similar gene structure patterns. For example, *CPK*s in group I have 7 exons, *CPKs* in group II have 8 exons, group III *CPK*s have 7 or 8 exons, and most *CPK*s in group IV have 11 exons. Usually, the conservation of gene structure within a gene group is closely related to their evolutionary relationship with a few exceptions, such as *GhCPK5* in group I, which has 16 exons, and *GhCPK20* in group IV, which has only 5. Additionally, the longest exon of most cotton *CPK* genes is exon 1. However, a small portion of the gene family members evolved apparently divergent structures. For example, *GhCPK18* and *GhCPK58* are homologues from different sub-genomes; *GhCPK18* has the typical gene structure, whereas *GhCPK58* has a short exon 1 and much longer intron 1. All of the above results are indicative of the occurrence of independent evolutionary events in some cotton *CPK*s. This structural divergence most likely evolved randomly and after the formation of upland cotton. The changing gene structure may have led to mutations at splice sites, resulting in novel transcripts or nonfunctional gene products. In other words, the novel transcripts may have disrupted ancestral functions, produced novel functions, or even have suppressed the parental homologue [31], thereby expanding the roles of the *CPK* gene family.

### Genomic distribution and synteny analysis of cotton *CPK* genes

**Fig. 3:**
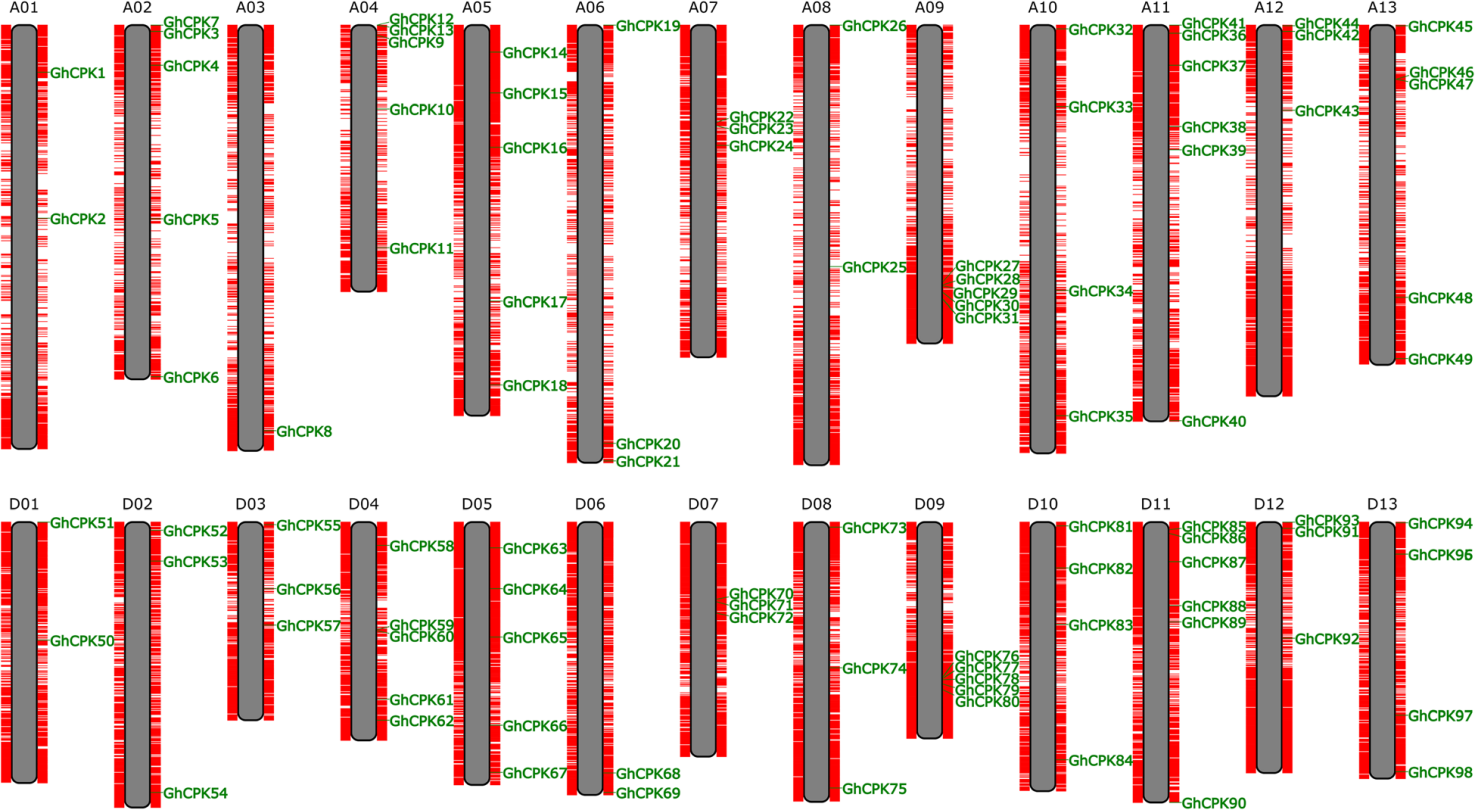
Chromosomal distribution of *GhCPK* genes in the cotton genome. The chromosome numbers are labeled at the top of each chromosome. The red lines on both sides of the chromosomes indicate the coding sequence of the cotton genome and tandem duplications, respectively. *GhCPK* genes were mapped onto cotton chromosomes and are marked with green lines

To investigate the chromosomal locations of these *CPK* genes, we analyzed 26 chromosomes of the published *G. hirsutum* genome [29, 30]. The 26 chromosomes were labeled A01 to A13 and D01 to D13. The genomic sequences of *CPK* genes were used to query the genome with BLAST to assess their genomic distribution. As shown in Fig. 3, 98 *CPK* genes were localized to 26 chromosomes. The distribution pattern of cotton *CPK* genes is non-random, with most of the homologous genes split evenly between the A and D genomes. However, the numbers of *CPK* genes of each chromosome are distributed unevenly. For example, A11 and D11 respectively contain six *CPK*s, but only one *CPK* gene localized to chromosome A03. Several *CPKs* appeared to be clustered together on specific chromosomes, such as chromosomes A09 and D09.

**Fig. 4:**
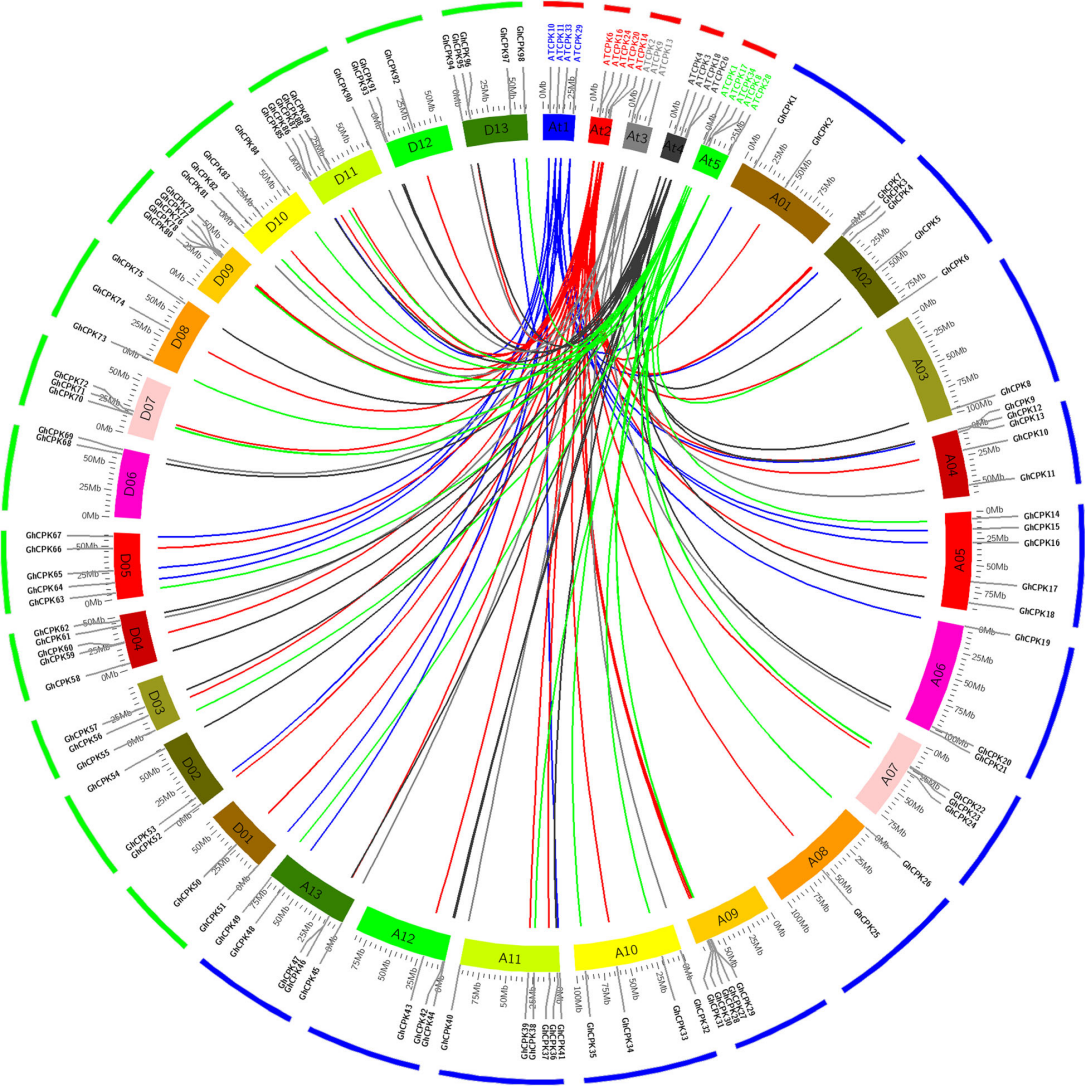
Synteny analysis of *G. hirsutum* and *A. thaliana CPK* genes. Chromosomes of *G. hirsutum* and *A. thaliana* are shown in a circular form with different colors. Approximate positions of *GhCPK* and *AtCPK* genes are labeled with a short gray line on the circle. Synteny between *GhCPK* and *AtCPK* genes is shown with colored curves

Throughout the process of gene family evolution, the duplication and divergence of gene members lead to functional diversity in biological processes carried out by the gene family [31]. To further investigate the origin and evolution of cotton *CPK* genes, we examined the syntenic relationship between cotton and *Arabidopsis* using Circos. As shown in Fig. 4, most of the cotton *CPK* genes exhibit synteny with *Arabidopsis CPK* homologues. As expected, several cotton *CPK* genes corresponded to individual *Arabidopsis* genes. For example, the regions containing *GhCPK31*/*GhCPK80*/*GhCPK2*/*GhCPK50*/*GhCPK12*/*GhCPK59* in *G. hirsutum* was syntenic to the region containing *ATCPK20* in *Arabidopsis*, while *GhCPK35*/*GhCPK84* and *ATCPK1* exhibited a similar relationship. These results demonstrate that cotton *CPK* genes duplicated during the evolutionary process and may have important roles in cotton development or defense response. This provides broader insights into the functional diversification of cotton *CPK* genes.

### Cotton *CPK* genes broadly expressed across different tissues

**Fig. 5:**
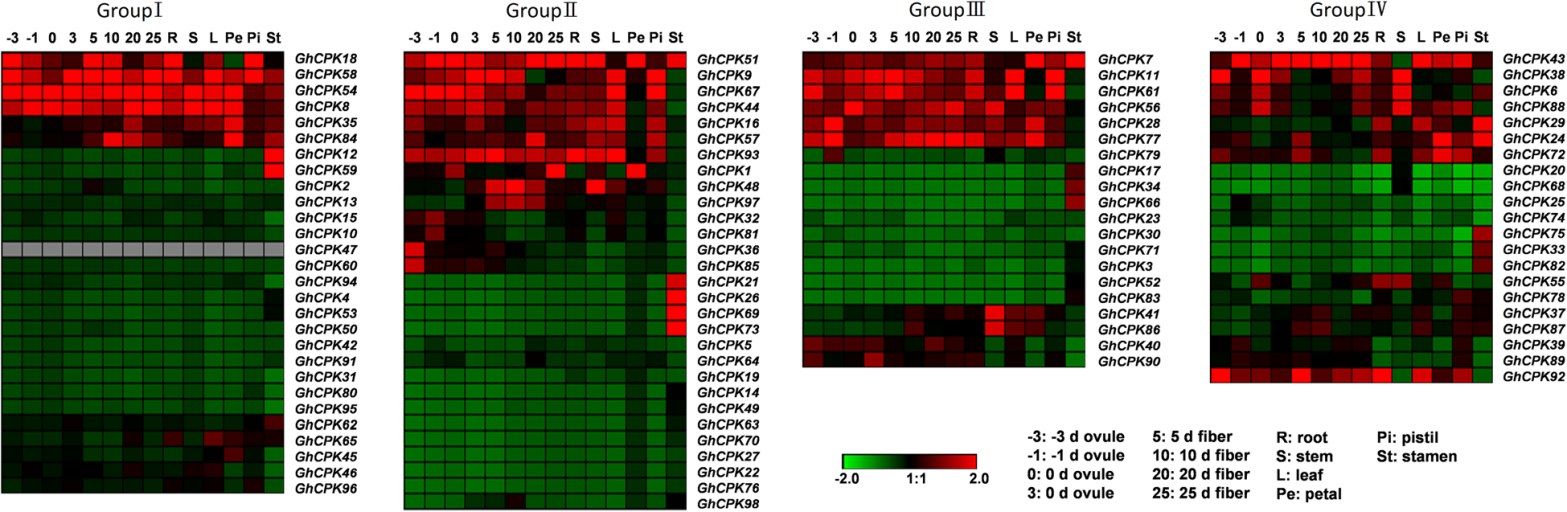
Expression patterns of *GhCPK* genes in 14 representative tissues from *G. hirsutum*. The heat map, generated with Genesis, shows the hierarchical clustering of *GhCPK* genes into four groups in vegetative organs (root, stem, and leaf), in floral organs (petal, pistil, and stamen), and at different developmental stages of ovule and fiber tissues. The reads per kilobase of transcript per million mapped reads (RPKM) values were log_10_ transformed and indicated the expression level of *GhCPK* genes, while the gradient color (red/black/green) reflects the expression levels (high to low)

Transient changes in Ca^2+^ concentrations activate CPKs, as is required for various biological proceses such as plant growth, development, reproduction, and defenses against biotic or abiotic stresses [4, 32]. It has also been reported that *CPK* genes are broadly expressed in different tissues, even in fruits, pollen, embryos, and guard cells [33, 34]. The expression levels of *CPK* genes in various tissues were analyzed using previously published transcriptome data to understand the possible functional roles of cotton CPKs, [30]. The reads per kilobase of transcript per million mapped reads (RPKM) values in 14 different tissues (root, stem, leaf, petal, pistil, stamen, as well as ovule and fiber at different development stages) are listed in Additional file 6. These values were used to create a heat-map of *CPK* genes’ expression. Substantial diversity is manifested in the expression profile of *CPK* members (Fig. 5). *GhCPK47* transcripts were not detected in any tissues, suggesting that it is a pseudogene or that its expression is induced only under particular conditions. Among the remaining *CPK* genes, more than half were expressed at a low level across all 14 tissues, and about 25% of *CPK*s were observed to have a comprehensive expression range with consistently high expression in most tissues. Additionally, a few genes exhibited tissue-specific expression. For example, four genes (*GhCPK21*, *GhCPK26*, *GhCPK69*, and *GhCPK73*) from group II had high expression in stamens but low expression in all other tissues, which suggested the possibility of important roles of these four genes in cotton reproductive biology. Similar patterns occurred in other groups, the genes with constitutive expression, little expression and tissue-specific expression occurred in all four groups, indicating massive functional diversification within the *CPK* gene family or even within the individual gene groups. As a consequence of gene duplication, which is widespread in tetraploid cotton genomes, the function of duplicated genes diverged in three different major ways: toward non functional or pseudogenized gene copies; toward new genes with novel functions; and toward homologous genes with similar or identical functions [30]. The heat-map indicates most homologues in the *CPK* family were conserved functionally; however, a few of them exhibited differentiated gene functions. For example, *GhCPK1* and *GhCPK51*, which have similar *pI* and Mw values as well as protein and gene structures, showed distinct expression pattern across the 14 tested tissues. *GhCPK51* has high expression across all tissues except leaves, whereas *GhCPK1* was highly expressed only in the 0 DPA (days post anthesis) ovule, 25 DPA fiber and petal. Conversely, some homologues, such as *GhCPK18* and *GhCPK58*, have evolved divergent gene structures and protein structures despite similar expression patterns. All the above results indicate a considerable functional diversification of the cotton *CPK* gene family.

### Salt stress and ethylene induced CPK expression

**Fig. 6:**
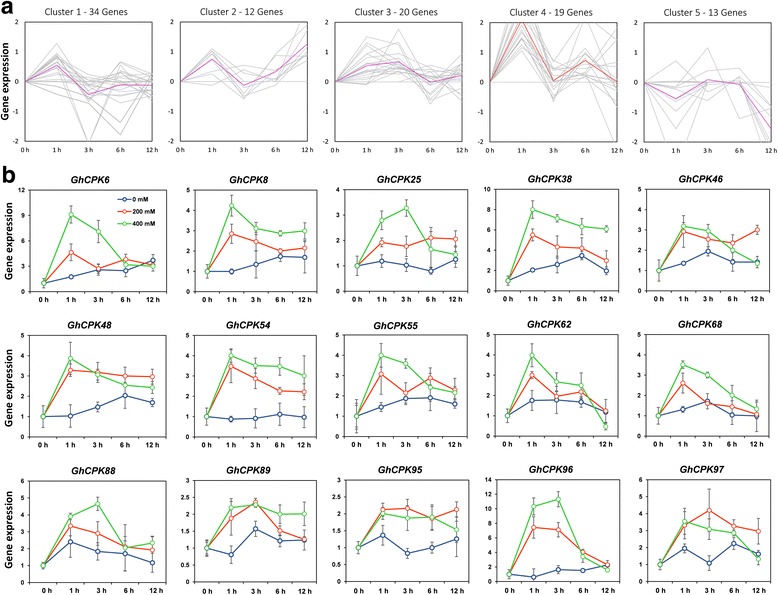
Expression profile of *GhCPK* genes in response to salt stress. **a** The cluster analysis was developed using the K-means method on the expression profiles for a total of 98 *CPK* genes in *G. hirsutum*. **b** qPCR analyses of cluster 4 *CPK* genes expression following NaCl treatment. Cotton seedlings were irrigated with water containing 0 mM, 200 mM or 400 mM NaCl, respectively. Cotton leaves were collected at 0, 1, 3, 6, and 12 h after the treatment. The *GhUB7* (*ubiquitin 7*, Accession: DQ116441) gene was used as an internal control. The error bars indicate the standard deviation estimated from the four replicates

Soil salinization is one of the major environmental constraints to crop production worldwide [1–3]. CPK proteins were previously demonstrated to have key roles in salt stress resistance in other plant species, but have rarely been examined in cotton [12–16]. The expression profiles of 98 *CPK* genes were analyzed to clarify the involvement of cotton CPKs in response to salt stress, (listed in Additional file 7). As shown in Fig. 6a, most of the cotton *CPK* genes were up-regulated after salt treatment. Under prolonged exposure to salt, the number of salt-induced genes decreased, indicating that CPKs may function in the early stage of cotton responses to salt. Based on their expression patterns in response to salinity, the cotton *CPK* genes can be divided into five clusters. Cluster 5 contained few genes, and they were down-regulated after salt treatment. In contrast, the genes in the other four clusters were all up-regulated. Notably, 19 *CPK* genes that were categorized into cluster 4 exhibited rapid changes in the very early stage (up-regulation, with peaks within 1 h) in response to salinity (Fig. 6a). This results suggest that these genes are likely involved in the early stages of salinity responses in cotton. Therefore, we focused on those genes for further analysis. qPCR analysis confirmed the induction of cluster 4 *CPK* genes by salt stress. From the initial 19 genes, 4 could not be amplified by PCR while the expression of the remaining 15 genes was clearly induced by treatment with two different salt concentrations, 200 mM and 400 mM NaCl (Fig. 6b). Some of the genes showed a dose-dependent level of induction at the two concentrations used in this study. We also performed bioinformatics analysis of the 1.5 kb promoter regions upstream of the start of transcription for the 19 cluster 4 *CPK*s and found multiple stress- and hormonal-related *cis*-elements in all the promoters (Additional file 8). Our results reflect the multiple stress responsive nature of cotton CPKs.

**Fig. 7:**
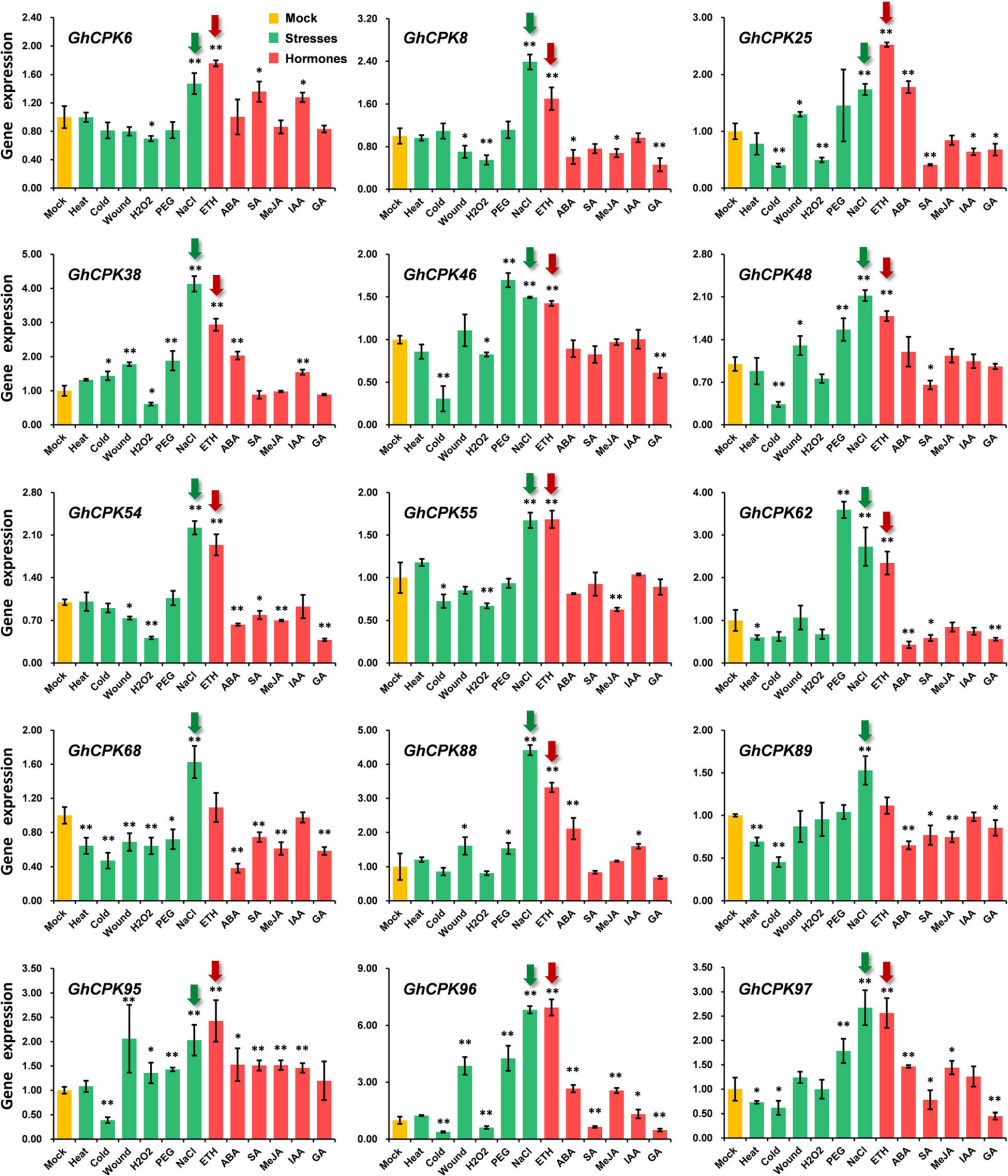
Abiotic stress and hormonal treatments induced expression of *CPK* genes. qPCR analysis of the relative expression level of the *CPK* genes in cluster 4. Cotton seedlings were treated with 100 mM H_2_O_2_, 100 μM ABA, 1 mM SA, 100 μM MeJA, 200 μM ETH, 5 μM IAA, 0.5 μM GA, 20% PEG, 400 mM NaCl, 4 °C (cold), 37 °C (heat), wounding, or water (as a control), respectively. The *GhUB7* gene was used as an internal control. The yellow, green and red bars represent mock, abiotic stresses and hormones treatments, respectively. The green and red arrows emphasize the significantly upregulated expression of target genes under salt and ETH treatments. The error bars indicate the standard deviation estimated from the four replicates (* P < 0.05, ** P < 0.01, *t*-test)

We further analyzed the expression of the 15 *CPK* genes under abiotic stress and hormonal treatments. As shown in Fig. 7, salt treatment induces the expression of all 15 genes. Notably, 13 out of the 15 genes were also induced by treatment with ethephon (ETH), a plant growth regulator that releases ethylene upon being metabolized by the plant [35]. A few recent reports have revealed the correlation between salt response and ethylene synthesis in plants. Under high salt conditions, the expression of ethylene synthases (*ACS5* and *ACS7*) was highly induced in *Arabidopsis* [36]. The ethylene-activated transcription factors *EIN3* (*ethylene insensitive 3*) and *EIL1* (*EIN3-Like 1*) play important roles in plant defenses to salt stress [37]. This indicates that regulation of the ethylene signal pathway allows plants to adjust their salt tolerance. The present study demonstrates that most cotton *CPK* genes that were rapidly induced by salt stress were also up-regulated by ETH treatment within 1 h. This suggests that there is a possible overlap between the signal networks of cotton responses to salt stress and ethylene. Moreover, this overlap occurs early in response to salinity.

### Cluster 4 CPKs localized to plasma membranes

**Fig. 8:**
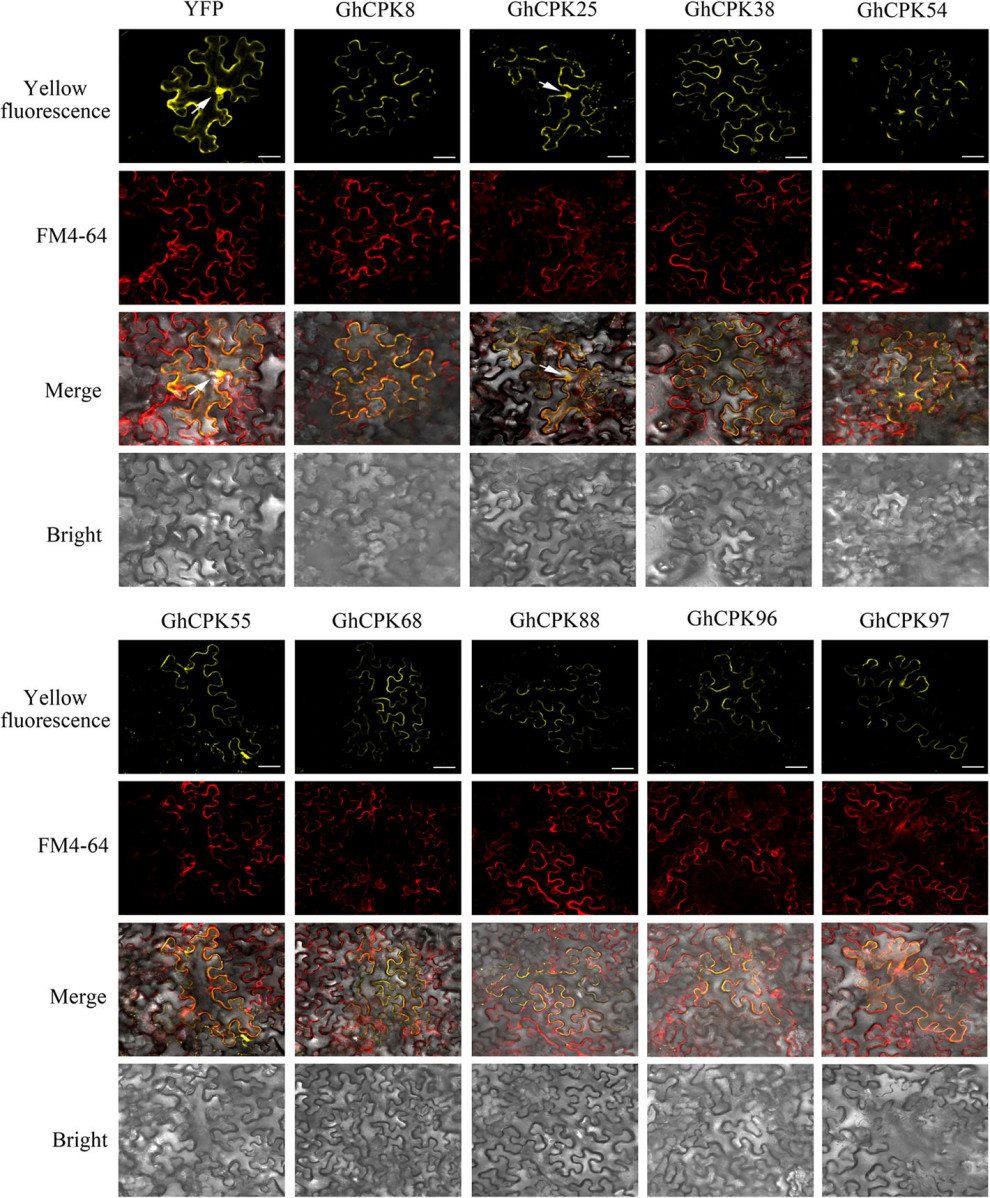
Subcellular localization of GhCPKs. Nine GhCPKs (GhCPK8, GhCPK25, GhCPK38, GhCPK54, GhCPK55, GhCPK68, GhCPK88, GhCPK96, and GhCPK97) that were predicted to be involved in salt response were fused with YFP at the C-terminus and transiently expressed in 3-week-old tobacco leaf cells. YFP driven by the CaMV35S promoter was used as a control. Yellow fluorescence was observed 48 h post-*Agrobacterium* infiltration using confocal microscopy. The plasma membrane labeling by FM4–64 is shown as red fluorescence, the YFP observed in cell nuclei were marked with white arrows (scale bar = 10 μm)

The subcellular localization of proteins is closely related to their functions. Previous research indicates that CPKs are widely distributed within plant cells, including within nuclei, plasmalemma, cytoplasm, endoplasmic reticulum membranes and peroxisomes [38–42]. This wide range of localizations illustrates the important role of CPKs in plants. Typical CPKs have an N-terminal variable domain. In some cases, it contains the *N*-myristoylation site (MGXXXSK) or/and the *S*-palmitoylation site [10]. These acylation sites are responsible for targeting CPK proteins to membranes. In the present study, the yellow fluorescent protein was fused to nine of the CPKs in cluster 4: GhCPK8, GhCPK25, GhCPK38, GhCPK54, GhCPK55, GhCPK68, GhCPK88, GhCPK96, and GhCPK97 (Fig. 8, Additional file 9). The plasma membrane of tobacco cells labeled with FM4–64, which is a lipophilic probe exhibits strong red fluorescence when specifically targets the membrane system [43]. Of the nine cotton CPK proteins, eight of them localized to the plasma membrane, except for GhCPK25, which was detected in both the plasma membrane and nucleus. Plants respond to external stimuli through signal transduction, and the speed of stimuli perception, signal transduction, and downstream responses determines whether the plant can survive in the constantly changing environment [2, 3]. The plasma membrane is one of the very first barriers in plant defense responses. Proteins located in the plasma membrane recognize surrounding signals while receiving extracellular stimuli rapidly and further transduce these stimuli into intracellular signals [32, 44]. The cotton *CPK* genes of cluster 4 were swiftly and notably induced by salt stress and also exhibited plasma membrane localization. Hence, cluster 4 CPKs are early salt responsive genes that may play a crucial role in responding to stresses via their rapid signal perception and transduction.

### *CPK* genes positively regulate salt resistance in cotton

**Fig. 9:**
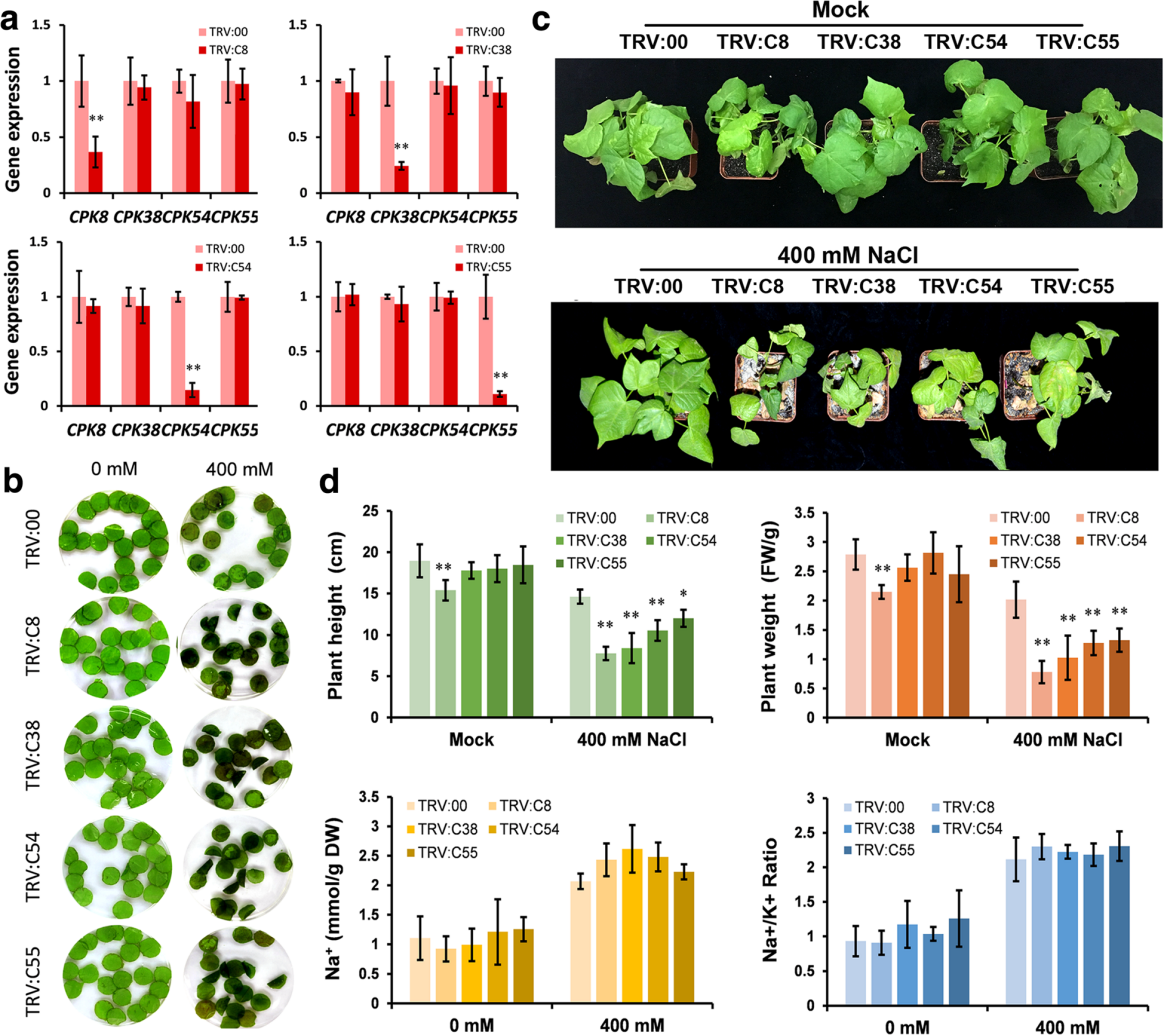
Functional analysis of cotton CPKs in response to salt stress. **a** Detection of *GhCPK* transcripts in TRV:00 and *CPK*-silenced plants. The cotton *UB7* gene was amplified as an internal control. The standard deviations were calculated from the results of three independent experiments (** *P* < 0.01, *t*-test). **b** Leaf disks of control and *CPK*-silenced plants were incubated in water supplemented with different concentrations of NaCl (0 mM and 400 mM) for 4 days. **c** Photographs of representative plants of control (TRV:00) and *CPK*-silenced seedlings (TRV:C8, TRV:C38, TRV:C54, and TRV:C55) under normal condition (Mock) and 400 mM NaCl treatment for 10 days with re-watering. **d** Measures of plant height and fresh weight, and quantification of Na^+^ and Na^+^/ K^+^ of the aerial portions of TRV:00 and *CPK*-silenced plants treated with water (control) or 400 mM NaCl for 10 days with re-watering. The standard deviations were calculated from the results of three independent experiments (*n* ≥ 16, * *P* < 0.05, ** *P* < 0.01, *t*-test)

The transcript levels of *CPK* genes in cluster 4 were significantly increased under salinity, with the highest peak observed within 1 h of the salt treatment. The observed gene expression patterns may reflect their functions [45]. The allotetraploid nature of the *G. hirsutum* genome and the high sequence homology displayed by many of its genes makes it sometimes difficult to amplify specific cDNAs. Despite repeated attempts, we could successfully amplify only nine of the CPKs present in cluster 4 (*GhCPK8*, *GhCPK25*, *GhCPK38*, *GhCPK54*, *GhCPK55*, *GhCPK68*, *GhCPK88*, *GhCPK96* and *GhCPK97*), all of which were subsequently silenced in cotton using the virus-induced gene silencing (VIGS) method, to further investigate their possible involvement in salt stress resistance [46, 47]. Some of the coding regions for the selected CPKs were highly homologous therefore we used the highly divergent 3’-UTRs for the VIGS constructs to ensure specific silencing of each *CPK* gene. Ten-days-old cotton seedlings were inoculated with *Agrobacterium* containing the VIGS vectors, and seedlings subjected to experimental treatments 2 weeks after inoculation. Silencing efficiency and specificity was analyzed by qPCR, and the results showed strong and specific silencing of the targeted genes (Fig 9a). To study the effect of silencing the selected CPKs in the overall resistance to salt stress in cotton, leaf discs from silenced plants were incubated in 0 or 400 mM NaCl solutions for 4 days. Silencing of *GhCPK25*, *GhCPK68*, *GhCPK88*, *GhCPK96* and *GhCPK97* did not produce any observable differences in the leaf discs compared to control treatments infected with an empty VIGS construct (TRV:00) (data not shown). In contrast, leaf discs from plants infected with constructs targeting *GhCPK8*, *GhCPK38*, *GhCPK54*, and *GhCPK55* showed significantly more browning than leaf discs from TRV:00 infected controls (see TRV:C8, TRV:C38, TRV:C54, and TRV:C55 in Fig 9b). The further investigate the effect of silencing in salt stress resistance, *CPK*-silenced plants were grown in pots and irrigated with 400 mM NaCl for 10 days. *CPK*-silenced plants showed increased sensitivity to salt stress compared to control plants (Fig. 9c, d). The Na^+^ and K^+^ content in cotton leaves were also measured. The Na^+^ concentration and Na^+^/K^+^ ratio in control and *CPK*-silenced plants were increased compared to mock treatment, suggesting that plants were suffering from severe salt stress after the treatment, however, no significant differences were found between control and *CPK*-silenced plants (Fig. 9d). The increased sensitivity to salt shown by the silenced lines strongly suggest a role for these CPKs in cotton’s resistance to salt although it is still unknown if all of them are involved in the same or different resistance mechanisms. Note that the TRV:C8 seedlings were smaller than TRV:00 and other *CPK*-silenced plants both with and without salt treatment (Fig. 9c), indicating the possible involvement of *GhCPK8* in cotton development aside from salt resistance.

The functions of *CPK* genes are diverse. *CPK* genes influence plant salt resistance by regulating the downstream components of calcium signaling pathways [14, 15 20]. Similarly, they are also involved in cold tolerance, pathogen defense, hormone responses, and so on [13, 32]. Four representative cotton *CPK* genes in cluster 4 were confirmed to positively regulate salt tolerance, which is consistent with the accumulated evidence from their expression profiles and protein localization patterns. However, the downstream components of CPKs, as well as physiological adaptaion mechanisms in cotton and their response to salt, remain to be examined in future studies.

## Conclusion

The *G. hirsutum* genome contains 98 putative CPKs, which are broadly distributed in the genome. Expression profiling identified 19 *CPKs* with significantly induced gene expression during the early stage of salt stress, 15 of which were further confirmed by qPCR. Interestingly, 13 out of the 15 CPKs also showed strong induction upon exposure to the ethylene-releasing chemical ETH, suggesting that they may be involved in the crosstalk between the salt stress and ethylene signaling networks. All studied CPKs are localized to the plasma membrane, consistent with a possible role in cell signaling. Silencing of some *CPKs* severely compromised cotton’s basal resistance to salt stress indicating their involvement in the resistance mechanism. This study aims to broaden our understanding of CPK functions and provides key insights for further research into the molecular mechanisms of cotton responses to salt stresses, as well as provididng candidate target genes for increasing salt-tolerance in cotton.

## Methods

### Identification of *CPK* genes from *G. hirsutum* L. TM-1

The reference genome of upland cotton (*G. hirsutum* L.) cultivar ‘TM-1’ was obtained from Nanjing Agricultural University’s CRI (http://mascotton.njau.edu.cn/info/1054/1118.htm). The CPK protein sequences of various species (*Arabidopsis*, rice, maize, and grape) were downloaded from NCBI (http://www.ncbi.nlm.nih.gov/) and used as reference sequences for building a Hidden Markov Model (HMM). To identify the cotton CPK proteins, the HMM was used as a query to search the upland cotton protein database using HMMER 3.1 (http://hmmer.janelia.org/).To further confirm the reliability of putative cotton CPKs, representative motifs of the CPK family were also characterized using SMART (http://smart.embl-heidelberg.de/) and InterPro (http://www.ebi.ac.uk/interpro/). The predicted molecular weight (Mw) and isoelectric point (*pI*) of each CPK were inferred using the compute *pI*/Mw tool (http://web.expasy.org/compute_pi/). The 1.5 kb DNA sequences upstream of the initiation codon of *CPK* genes were downloaded from CRI, and cis-regulatory elements prediction were performed using online software PlantCARE (http://bioinformatics.psb.ugent.be/webtools/plantcare/html/).

### Phylogenetic analysis and gene structure prediction

Multiple sequence alignments of cotton CPKs were produced using Clustal W [48]. The phylogenetic tree was generated with the aligned sequences using the neighbor-joining method in MEGA6 with 1000 bootstrap replicates [49]. The genomic DNA and corresponding CDS sequences of cotton *CPK*s were obtained from Nanjing Agricultural University’s CRI (http://mascotton.njau.edu.cn/info/1054/1118.htm). The gene structures concerning intron–exon organization were inferred using the Gene Structure Display Server (GSDS), a web-based bioinformatics tool (http://gsds.cbi.pku.edu.cn/).

### Chromosomal localization and synteny analysis of CPKs in cotton

The chromosomal locations of cotton *CPK* genes were obtained from the cotton genome database. Subsequently, the cotton *CPK* genes were mapped onto chromosomes according to their chromosomal positions using the MapInspect program (http://mapinspect.sharewarejunction.com/), a tool for drawing chromosomal locations. Circos [50] was used to generate a syntenic diagram illustrating the genomic distribution of *CPK* genes and synteny relationships between cotton and *Arabidopsis*.

### Heat-map analysis

The reads per kilobase of transcript per million mapped reads (RPKM) values representing the expression levels of *CPK*s were collected from the ‘TM-1’ cultivar transcriptome data downloaded from NCBI (http://www.%20ncbi.nlm.nih.gov/sra/?term=PRJNA248163). The gene expression data were analyzed using the Genesis 1.8.1 program to generate heat maps [51]. The cluster analysis, which was developed using the K-means method on the expression profiles of all 98 *CPK* genes, was also performed using the Genesis program.

### Plant materials and treatments

Cotton seeds were germinated in a high humidity environment at 28 °C for 48 h and then transferred to soil in an incubator at 22/25 °C (night/day). After 4 weeks, the well-growing cotton seedlings were treated with abiotic stresses and phytohormones. 100 μM ABA, 1 mM SA, 100 μM MeJA, 200 μM ETH, 5 μM IAA, 0.5 μM GA was applied for homonal treatments by using pure water as a control. The ETH was employed as an ethylene-release agent in our experiments [35, 52]. For oxidative stress treatments, cotton plants were sprayed with 100 mM H_2_O_2_. For salt stress, the seedlings were irrigated with 200 or 400 mM NaCl, respectively. For wound treatment assays, leaves were injured with scissors as previously described [52]. For cold and heat stresses treatments, plants were moved into 4 °C and 37 °C incubators, respectively. Osmotic stress treatments were also performed by irrigating seedlings with 20% PEG6000. The second true leaves of treated cotton seedlings were harvested 1 h after the treatments were initiated and stored at − 80 °C until further experiments were performed. Each treatment included five seedlings, and there were three experimental replicates of each treatment.

Tobacco (*Nicotiana benthamiana*) seeds were also germinated and grown in soil-filled pots in an incubator at 22 °C/25 °C (night/day). After 3 weeks, young leaves of tobacco plants were used to determine the subcellular localization of cotton CPKs. The detailed procedures of subcellular localization were performed as reported previously [46].

### RNA isolation and qPCR

Total RNA was isolated from fresh 0.1 g samples using the EASYspin Plus plant RNA kit (AidLab, Beijing, China) according to the manufacturer’s instructions. First strand cDNAs were synthesized from 1 μg of total RNA using the ReverTra Ace qPCR RT kit (TOYOBO, Osaka, Japan). The reverse transcription product was diluted 30-fold with sterile distilled water and stored at − 20 °C before further experiments. Then, four replicates of qPCR were performed using AceQ qPCR SYBR Green Master Mix (Vazyme, Nanjing, China) on an ABI 7500-Fast Real Time PCR system (Applied Biosystems, Foster City, CA, USA). As described in our previous publication [52], serial dilutions of cDNA were amplified using primers of the target genes to examine their PCR amplification efficiency. The cotton *Ubiquitin7* gene (*UB7*) was used as a reference gene to normalize the cDNA amplification in each reaction, and the relative changes in the target genes were calculated using the 2^-∆∆Ct^ method [53].

### Subcellular location

The CDSs of *CPK*s were amplified from the ‘TM-1’ cotton cDNAs using Phanta Super-Fidelity DNA Polymerase (Vazyme, Najing, China). Then, the CPK genes were fused to the C-terminal of a YFP Vector (pGWB441) to generate the fusion expression vectors. Meanwhile, the 35S–YFP vector was used as a control. The constructed vectors were introduced into *Agrobacterium tumefaciens* strain GV3101. Three-week-old tobacco leaves were infiltrated with GV3101 containing the fusion expression vectors. After 48 h, yellow fluorescence was observed using a confocal microscope (Leica Microsystems TCS SP2 AOBS; Leica Microsystems GmbH, Wetzlar, Germany). The samples were stained with FM6–64 for 10 min to mark the membrane before fluorescence observation.

### VIGS in cotton

The silencing fragments of each *CPK* gene were amplified from cotton and inserted into the VIGS vector (TRV:00) to generate the *CPK*-silencing constructs [46, 47]. TRV:00 with no silencing fragment was used as a control. Subsequently, the vectors were transformed into *A. tumefaciens* strain GV3101. The GV3101 contains TRV:00 and TRV: CPKs were mixed in equal amounts and infiltrated into the cotyledons of 10-day-old cotton seedlings by syringe infiltration. The silenced cotton seedlings were used for gene expression analysis and NaCl treatment at 2-weeks after inoculation. For the measurement of Na^+^ and K^+^ content, the leaves of cotton seedlings were harvested after water and NaCl treatments. The followed steps were performed following the description of Rus et al. [54].

## Additional files

Additional file 1: Summary of cotton CPKs. (XLSX 15 kb)

Additional file 2: CPK proteins used for phylogenetic analysis. (DOCX 55 kb)

Additional file 3: Sequence alignments showing the homologies GhCPK21 and GhCPK69. The alignments were performed using DNAMAN software. Identical amino acids are indicated with black highlighting. (TIFF 96 kb)

Additional file 4: The multiple sequence alignments showing the homologies of GhCPK14, GhCPK22, GhCPK63, and GhCPK70. The alignments were performed using DNAMAN software. Identical amino acids are shown with dark blue highlighting. (TIFF 192 kb)

Additional file 5: The phylogenetic tree of group IV CPKs. The un-rooted tree was generated using MEGA6.0 with the neighbor-joining method (1000 bootstrap replicates). CPKs from different species are denoted with differently colored rhombuses. (TIFF 2051 kb)

Additional file 6: Reads per kilobase of transcript per million mapped reads (RPKM) values of *CPK* genes in different cotton tissues. (XLSX 22 kb)

Additional file 7: Reads per kilobase of transcript per million mapped reads (RPKM) values of *CPK* genes in cotton leaves under salt stress. (XLSX 19 kb)

Additional file 8: The *cis*-elements prediction of *CPKs* promoter. (XLSX 11 kb)

Additional file 9: Subcellular localization of GhCPKs without FM4–64 staining. The YFP observed in cell nuclei were marked with red arrows (scale bar = 10 μm). (PNG 3100 kb)

## Abbreviations

ABA: Abscisic acid
CaM-LD: Calmodulin-like domain
CDS: Coding sequence
CPK: Calcium-dependent protein kinase
DPA: Days post anthesis
ETH: Ethephon
GA: Gibberellic acid
Gh: *Gossypium hirsutum*
HMM: Hidden Markov Model
IAA: Auxin
MeJA: Methyl jasmonate
Mw: Molecular weight
PCR: Polymerase chain reaction
PEG: Polyethylene glycol
pI: Isoelectric point
ROS: Reactive oxygen species
SA: Salicylic acid
VIGS: Virus-induced gene silencing
