# Supplementary material for: Calcium-dependent protein kinases in cotton: insights into early plant responses to salt stress
Source: BMC Plant Biol. 2018 Jan 17;18:15. doi: 10.1186/s12870-018-1230-8 (PMC5772696; doi:10.1186/s12870-018-1230-8)
Supplement: Supplementary file 2 — CPK proteins used for phylogenetic analysis. (DOCX 55 kb) [file 12870_2018_1230_MOESM2_ESM.docx]

**Additional file 2:** CPK proteins used for phylogenetic analysis.

>VvCDPK3

MGACLSATKVRSSNSNTTANAAATKNTRPRGSSKTSNKNQQKKPQEGDRNRSNQQHRNPQPQKVKDRANGRRGTGIIPCGKRTDFGYAKDFDARYTIGKLLGHGQFGYTYVATDKANGDRVAVKRIEKNKMILPIAVEDVKREVKILEALTGHENVVQFHNAFEDDSYVYIVMELCEGGELLDRILAKKDSRYSEKDAAKVVRQMLKVAAECHLHGLVHRDMKPENFLFKSTKEDSPLKATDFGLSDFIKPGKKFQDIVGSAYYVAPEVLRRKSGPESDVWSIGVITYILLCGKRPFWDKTEDGIFKEVLKNKPDFRRKPWPTISNGAKDFVKKLLVKDPRARLTAAQALSHPWVREGGDASEIPIDISVLSNMREFVKYSHLKQFALRALASTLDDEELADLRDQFDAIDVDKNGSISLEEMRQALAKDLPWKMKESRVLEILQAIDSNTDGLVDFTEFVAATLHVHQLEEHDSDKWRQRSQAAFDKFDVDRDGFITPEELKLHTGLRGSIDPLLEEADIDKDGRISLAEFRRLLRTASISSRQVPSSSGFRNPRKI

>VvCDPK18

MGLCQGKPIENPQTQSQDLIIPGDGELSTNTQTTKTPKFPFYSPSPLPSGFKNSPANSSVSSTPLRIFKRPFPPPSPAKHIRALLARRHGSVKPNEATIPEGNECEVGLDKNFGFSKQFVAHYEMGEEVGRGHFGYTSSAKAKKGSLKGQDVAVKVIAKSKMTTAIAIEDVRREVKILRALTGHKNLVQFYEAYEDDDNVYIVMELCRGGELLDRILARGGKYSEEDAKAVMVQILNVTAFCHLQGVVHRDLKPENFLFISKEENSPLKAIDFGLSDYVKPDERLNDIVGSAYYVAPEVLHRSYGTEADMWSVGVIAYILLCGSRPFWARTESGIFKAVLKADPSFDEAPWPSLSSDAIDFVKRLLNKDYRKRLTAAQALSHPWLANYHDVKIPLDMIVYKLVKAYIFSSSLRKSALAALAKTLSIAQLAYFREQFTLLGPNKSGFVSLQNFKTAVTKNSTDAIKDSRVLEYVSMVGSLQYRKLDFEEFCAAAISVLQLEGMESWEQHARRGYELFEKDGNRPIMIEELASELGLSPSVPVHVVLQDWIRHSDGKLSFLGFVRLLHGPSRAFPKA

>VvCDPK19

MGQETRRLLDEYEVSDVLGRGGFSVVRRGTRKSSSGENPVAIKTLKRCGQTNLPGLPRNRGSEKRVASMAFPTWKQVSISDALLTNEILVMRKIVEHVSPHPNVINLHDVYEDPSGVHLVLELCSGGELFDRIVAQARYSEAGAAAVVKQLAEGLKALHQANIIHRDLKPENCLFLDKSEDATLKIMDFGLSSVEEFTDPVVGLFGSIDYVSPEVLSQGKISSASDMWSLGVILYILLSGYPPFIAQSNRQKQQMIIAGDFSFYEKTWKNISSSAKQLISSLLTVDPERRPTAHQLLQHPWVMGDSAKQDQMDAEIVSRLQSFNARRKFRAAAIASVWSSTVFLRTKKLKTLVGSHDLTQEELENLRIHFKEICLKGDNATLSEFEQVLKAMNMSSLIPLAGRIFDLFDNNRDGTVDMREILCGFSSLRNSQGDDALRLCFQMYDTDRSGCITKEEVASMLRALPDDCLPADITEPGKLDEIFDLMDANSDGKVTFEEFKTAMQRDSSLQDVVLSSLRPL

>OsCPK4

MGACFSSHTATAAADGGSGKRQQRKGDHKGKLPDGGGGEKEKEAARVEFGYERDFEGRYQVGRLLGHGQFGYTFAATDRASGDRVAVKRIDKAKMVRPVAVEDVKREVKILKELKGHENIVHFYNAFEDDSYVYIVMELCEGGELLDRILAKKNSRYSEKDAAVVVRQMLKVAAECHLHGLVHRDMKPENFLFKSTKEDSPLKATDFGLSDFIKPGKKFHDIVGSAYYVAPEVLKRRSGPESDVWSIGVITYILLCGRRPFWNKTEDGIFREVLRNKPDFRKKPWPGISSGAKDFVKKLLVKNPRARLTAAQALSHPWVREGGEASEIPVDISVLSNMRQFVKYSRFKQFALRALASTLKEEELADLKDQFDAIDVDKSGSISIEEMRHALAKDLPWRLKGPRVLEIIQAIDSNTDGLVDFEEFVAATLHIHQMAELDSERWGLRCQAAFSKFDLDGDGYITPDELRMVQHTGLKGSIEPLLEEADIDKDGRISLSEFRKLLRTASMSNLPSPRGPPNPQPL

>OsCPK18

MGLCSSSSARRDAGTPGGGNGAGNKDNAGRKGIVACGKRTDFGYDKDFEARYALGKLLGHGQFGYTFAAVDRRSSERVAVKRIDKNKMVLPVAVEDVKREVKILKALQGHENVVHFYNAFEDDNYVYIVMELCEGGELLDRILAKKDSRYSEKDAAVVVRQMLKVAAECHLHGLVHRDMKPENFLFKSTKEDSSLKATDFGLSDFIRPGKHFRDIVGSAYYVAPEVLKRKSGPESDVWSIGVITYILLCGRRPFWDKTEDGIFKEVLKNKPDFRRKPWPNITPCAKDFVQKLLVKDPRARLTAAQALSHEWVREGGQASDIPLDISVLHNMRQFVKYSRFKQFALRALASTLNAEELSDLRDQFNAIDVDKNGTISLEELKQALAKDVPWRLKGPRVLEIVEAIDSNTDGLVDFEEFVAATLHVHQLVEHDTEKWKSLSQAAFDKFDVDGDGYITSDELRMQTGLKGSIDPLLEEADIDRDGKISLDEFRRLLKTASMSSRNVQTPRSVHRS

>ZmCPK38

MGLCSSSTAARAASDPGAAAAGDAAAAKKGRGIVACGKRTDFGYDKDFEVRYSLGKLLGHGQFGYTFAAVDRASTERVAVKRIDKNKMVLPVAVEDVKREVKILKALQGHENVVHFYNAFEDDNYVYIVMELCEGGELLDRILAKKDSRYSEKDAAVVVRQMLKVAAECHLHGLVHRDMKPENFLFKSKKEDSPLKATDFGLSDFIKPGRQFRDIVGSAYYVAPEVLKRRSGPESDVWSIGVITYILLCGRRPFWDKTEDGIFKEVLKKKPDFRRKPWSNITSSAKDFVQKLLVKDPRARLTAAQALSHDWVREGGKASEIPLDISVLHNMRQFVKYSRFKQFALRALASTLNSEEMSDLRDQFNAIDVDKNGTISLEELKQALAKDVPWRLKGPRVLEIIEAIDSNTDGLVDFEEFVAATLHVHQLVEHDTEKWKSLSQAAFDKFDVDRDGYITPDELRMHTGMKGSIDPLLEEADIDKDGKISLDEFRRLLKTASMSARNVQTPRGVRKS

>ZmCPK39

MGACFSSASAAPAGAAVDERRPSKEGDGKKRRRAAGASPDAAAPVRVEFGYERDFEARYEVGRLLGHGQFGYTFAATDRGSGDRVAVKRIDKAKMTRPVAVEDVKREVKILKALKGHQNIVHFYNAFEDDSYVYIVMELCEGGELLDRILAKKNSRYSEKDAAVVVRQMLKVAAECHLRGLVHRDMKPENFLFKSNKEDSPLKATDFGLSDFIKPGKKFHDIVGSAYYVAPEVLKRRSGPESDVWSIGVITYILLCGRRPFWDKTEDGIFKEVLRNKPDFRKRPWSSISPGAKDFVKRLLVKNPRARLTAAQALSHPWVREGGEASDIPVDISVLSNMRQFVKYSRFKQFALRALASTLNEEELSDLKDQFDAIDIDKSGSISIEEMRHALAKDLPWRLKGPRVLEIIQAIDSNTDGLVDFKEFVAATLHIHQMAELDSERWGIRCQAAFSKFDLDGDGYITPEELRMVQHTGLKGSIEPLLEEADIDKDGKISLSEFRKLLRTASMSNVPSPRGPPNPQAL

>ZmCPK40

MGACFSSASAAPAGAAVDERRPSKEGDGKKRRRAAGASPDAAAPVRVEFGYERDFEARYEVGRLLGHGQFGYTFAATDRGSGDRVAVKRIDKAKMTRPVAVEDVKREVKILKALKGHQNIVHFYNAFEDDSYVYIVMELCEGGELLDRILAKKNSRYSEKDAAVVVRQMLKVAAECHLRGLVHRDMKPENFLFKSNKEDSPLKATDFGLSDFIKPGKKFHDIVGSAYYVAPEVLKRRSGPESDVWSIGVITYILLCGRRPFWDKTEDGIFKEVLRNKPDFRKRPWSSISPGAKDFVKRLLVKNPRARLTAAQALSHPWVREGGEASDIPVDISVLSNMRQFVKYSRFKQFALRALASTLNEEELSDLKDQFDAIDIDKSGSISIEEMRHALAKDLPWRLKGPRVLEIIQAIDSNTDGLVDFKEFVAATLHIHQMAELDSERWGIRCQAAFSKFDLDGDGYITPEELRMVQHTGLKGSIEPLLEEADIDKDGKISLSEFRKLLRTASMSNVPSPRGPPNPQAL

>AtCPK10

MGNCNACVRPDSKESKPSSKPKKPNRDRKLNPFAGDFTRSPAPIRVLKDVIPMSNQTQISDKYILGRELGRGEFGITYLCTDRETHEALACKSISKRKLR

TAVDIEDVRREVAIMSTLPEHPNVVKLKASYEDNENVHLVMELCEGGELFDRIVARGHYTERAAAAVARTIAEVVMMCHSNGVMHRDLKPENFLFANKKE

NSPLKAIDFGLSVFFKPGDKFTEIVGSPYYMAPEVLKRDYGPGVDVWSAGVIIYILLCGVPPFWAETEQGVALAILRGVLDFKRDPWPQISESAKSLVKQ

MLDPDPTKRLTAQQVLAHPWIQNAKKAPNVPLGDIVRSRLKQFSMMNRFKKKVLRVIAEHLSIQEVEVIKNMFSLMDDDKDGKITYPELKAGLQKVGSQL

GEPEIKMLMEVADVDGNGFLDYGEFVAVIIHLQKIENDELFKLAFMFFDKDGSTYIELDELREALADELGEPDASVLSDIMREVDTDKDGRINYDEFVTM

MKAGTDWRKASRQYSRERFKSLSINLMKDGSLHLHDALTGQTVPV

>ATCPK11

METKPNPRRPSNTVLPYQTPRLRDHYLLGKKLGQGQFGTTYLCTEKSTSANYACKSIPKRKLVCREDYEDVWREIQIMHHLSEHPNVVRIKGTYEDSVFV

HIVMEVCEGGELFDRIVSKGHFSEREAVKLIKTILGVVEACHSLGVMHRDLKPENFLFDSPKDDAKLKATDFGLSVFYKPGQYLYDVVGSPYYVAPEVLK

KCYGPEIDVWSAGVILYILLSGVPPFWAETESGIFRQILQGKLDFKSDPWPTISEAAKDLIYKMLERSPKKRISAHEALCHPWIVDEQAAPDKPLDPAVL

SRLKQFSQMNKIKKMALRVIAERLSEEEIGGLKELFKMIDTDNSGTITFEELKAGLKRVGSELMESEIKSLMDAADIDNSGTIDYGEFLAATLHMNKMER

EENLVAAFSYFDKDGSGYITIDELQSACTEFGLCDTPLDDMIKEIDLDNDGKIDFSEFTAMMRKGDGVGRSRTMMKNLNFNIADAFGVDGEKSDD

>ATCPK33

MGNCLAKKYGLVMKPQQNGERSVEIENRRRSTHQDPSKISTGTNQPPPWRNPAKHSGAAAILEKPYEDVKLFYTLSKELGRGQFGVTYLCTEKSTGKRFA

CKSISKKKLVTKGDKEDMRREIQIMQHLSGQPNIVEFKGAYEDEKAVNLVMELCAGGELFDRILAKGHYSERAAASVCRQIVNVVNICHFMGVMHRDLKP

ENFLLSSKDEKALIKATDFGLSVFIEEGRVYKDIVGSAYYVAPEVLKRRYGKEIDIWSAGIILYILLSGVPPFWAETEKGIFDAILEGEIDFESQPWPSI

SNSAKDLVRRMLTQDPKRRISAAEVLKHPWLREGGEASDKPIDSAVLSRMKQFRAMNKLKKLALKVIAENIDTEEIQGLKAMFANIDTDNSGTITYEELK

EGLAKLGSRLTEAEVKQLMDAADVDGNGSIDYIEFITATMHRHRLESNENVYKAFQHFDKDGSGYITTDELEAALKEYGMGDDATIKEILSDVDADNDGR

INYDEFCAMMRSGNPQQPRLF

>ATCPK19

MGCLCINLKKKVKKPTPDISGEQNTEVKSREITPKEQPRQRQPAPRAKFQIVVQPHKLPLPLPQPQEKQKLINHQKQSTLQQPEPILGRPFEDIKEKYSL

GRELGRGQFGITYICTEISSGKNFACKSILKRKLIRTKDREDVRREIQIMHYLSGQPNIVEIKGAYEDRQSVHLVMELCEGGELFDKITKRGHYSEKAAA

EIIRSVVKVVQICHFMGVIHRDLKPENFLLSSKDEASSMLKATDFGVSVFIEEGKVYEDIVGSAYYVAPEVLKRNYGKAIDIWSAGVILYILLCGNPPFW

AETDKGIFEEILRGEIDFESEPWPSISESAKDLVRNMLKYDPKKRFTAAQVLEHPWIREGGEASDKPIDSAVLSRMKQLRAMNKLKKLAFKFIAQNLKEE

ELKGLKTMFANMDTDKSGTITYDELKSGLEKLGSRLTETEVKQLLEDADVDGNGTIDYIEFISATMNRFRVEREDNLFKAFQHFDKDNSGFISRQELETA

MKEYNMGDDIMIKEIISEVDADNDGSINYQEFCNMMKSCSQSHQSKLVQPN

>ATCPK30

MGNCIACVKFDPDNSKPNQKKKPPRGRQRNPYDDPDGLRTHAPLRVIPMSHQSQISDKYILGRELGRGEFGITYLCTDRETREALACKSISKRKLRTAVD

VEDVRREVTIMSTLPEHPNVVKLKATYEDNENVHLVMELCEGGELFDRIVARGHYTERAAATVARTIAEVVRMCHVNGVMHRDLKPENFLFANKKENSAL

KAIDFGLSVLFKPGERFTEIVGSPYYMAPEVLKRNYGPEVDVWSAGVILYILLCGVPPFWAETEQGVALAILRGVLDFKRDPWSQISESAKSLVKQMLEP

DSTKRLTAQQVLDHPWIQNAKKAPNVPLGDIVRSRLKQFSMMNRLKKKALRVIAEHLSIQEVEVIRNMFTLMDDDNDGKISYLELRAGLRKVGSQLGEPE

IKLLMEVADVNGNGCLDYGEFVAVIIHLQKMENDEHFRQAFMFFDKDGSGYIESEELREALTDELGEPDNSVIIDIMREVDTDKDGKINYDEFVVMMKAG

TDWRKASRQYSRERFKSLSLNLMKDGSMHLHDALTGQSIAV

>ATCPK29

MLQNQHKTTKNQRNKNIGTKYFLRKKIMGFCFSKFGKSQTHEIPISSSSDSSPPHHYQPLPKPTVSQGQTSNPTSNPQPKPKPAPPPPPSTSSGSQIGPI

LNRPMIDLSALYDLHKELGRGQFGITYKCTDKSNGREYACKSISKRKLIRRKDIEDVRREVMILQHLTGQPNIVEFRGAYEDKDNLHLVMELCSGGELFD

RIIKKGSYSEKEAANIFRQIVNVVHVCHFMGVVHRDLKPENFLLVSNEEDSPIKATDFGLSVFIEEGKVYRDIVGSAYYVAPEVLHRNYGKEIDVWSAGV

MLYILLSGVPPFWGETEKTIFEAILEGKLDLETSPWPTISESAKDLIRKMLIRDPKKRITAAEALEHPWMTDTKISDKPINSAVLVRMKQFRAMNKLKKL

ALKVIAENLSEEEIKGLKQTFKNMDTDESGTITFDELRNGLHRLGSKLTESEIKQLMEAADVDKSGTIDYIEFVTATMHRHRLEKEENLIEAFKYFDKDR

SGFITRDELKHSMTEYGMGDDATIDEVINDVDTDNDGRINYEEFVAMMRKGTTDSDPKLIR

>ATCPK6

MGNSCRGSFKDKIYEGNHSRPEENSKSTTTTVSSVHSPTTDQDFSKQNTNPALVIPVKEPIMRRNVDNQSYYVLGHKTPNIRDLYTLSRKLGQGQFGTTY

LCTDIATGVDYACKSISKRKLISKEDVEDVRREIQIMHHLAGHKNIVTIKGAYEDPLYVHIVMELCAGGELFDRIIHRGHYSERKAAELTKIIVGVVEAC

HSLGVMHRDLKPENFLLVNKDDDFSLKAIDFGLSVFFKPGQIFKDVVGSPYYVAPEVLLKHYGPEADVWTAGVILYILLSGVPPFWAETQQGIFDAVLKG

YIDFDTDPWPVISDSAKDLIRKMLCSSPSERLTAHEVLRHPWICENGVAPDRALDPAVLSRLKQFSAMNKLKKMALKVIAESLSEEEIAGLRAMFEAMDT

DNSGAITFDELKAGLRRYGSTLKDTEIRDLMEAADVDNSGTIDYSEFIAATIHLNKLEREEHLVSAFQYFDKDGSGYITIDELQQSCIEHGMTDVFLEDI

IKEVDQDNDGRIDYEEFVAMMQKGNAGVGRRTMKNSLNISMRDV

>ATCPK16

MGLCFSSAAKSSGHNRSSRNPHPHPPLTVVKSRPPRSPCSFMAVTIQKDHRTQPRRNATAKKTPTRHTPPHGKVREKVISNNGRRHGETIPYGKRVDFGY

AKDFDHRYTIGKLLGHGQFGYTYVATDKKTGDRVAVKKIDKAKMTIPIAVEDVKREVKILQALTGHENVVRFYNAFEDKNSVYIVMELCEGGELLDRILA

RKDSRYSERDAAVVVRQMLKVAAECHLRGLVHRDMKPENFLFKSTEEDSPLKATDFGLSDFIKPGKKFHDIVGSAYYVAPEVLKRRSGPESDVWSIGVIS

YILLCGRRPFWDKTEDGIFKEVLKNKPDFRRKPWPTISNSAKDFVKKLLVKDPRARLTAAQALSHPWVREGGDASEIPIDISVLNNMRQFVKFSRLKQFA

LRALATTLDEEELADLRDQFDAIDVDKNGVISLEEMRQALAKDHPWKLKDARVAEILQAIDSNTDGFVDFGEFVAAALHVNQLEEHDSEKWQQRSRAAFE

KFDIDGDGFITAEELRMHTGLKGSIEPLLEEADIDNDGKISLQEFRRLLRTASIKSRNVRSPPGYLISRKV

>ATCPK24

MGSCVSSPLKGSPFGKRPVRRRHSSNSRTSSVPRFDSSTNLSRRLIFQPPSRVLPEPIGDGIHLKYDLGKELGRGEFGVTHECIEISTRERFACKRISKE

KLRTEIDVEDVRREVEIMRCLPKHPNIVSFKEAFEDKDAVYLVMEICEGGELFDRIVSRGHYTERAAASVAKTILEVVKVCHEHGVIHRDLKPENFLFSN

GTETAQLKAIDFGLSIFFKPAQRFNEIVGSPYYMAPEVLRRNYGPEIDVWSAGVILYILLCGVPPFWAETEEGIAHAIVRGNIDFERDPWPKVSHEAKEL

VKNMLDANPYSRLTVQEVLEHPWIRNAERAPNVNLGDNVRTKIQQFLLMNRFKKKVLRIVADNLPNEEIAAIVQMFQTMDTDKNGHLTFEELRDGLKKIG

QVVPDGDVKMLMDAADTDGNGMLSCDEFVTLSIHLKRMGCDEHLQEAFKYFDKNGNGFIELDELKVALCDDKLGHANGNDQWIKDIFFDVDLNKDGRISF

DEFKAMMKSGTDWKMASRQYSRALLNALSIKMFKEDFGDNGPKSHSMEFPIARKRAKLLDAPKNKSMELQISKTYKPSGLRN

>ATCPK25

MGNVCVHMVNNCVDTKSNSWVRPTDLIMDHPLKPQLQDKPPQPMLMNKDDDKTKLNDTHGDPKLLEGKEKPAQKQTSQGQGGRKCSDEEYKKRAIACANS

KRKAHNVRRLMSAGLQAESVLKTKTGHLKEYYNLGSKLGHGQFGTTFVCVEKGTGEEYACKSIPKRKLENEEDVEDVRREIEIMKHLLGQPNVISIKGAY

EDSVAVHMVMELCRGGELFDRIVERGHYSERKAAHLAKVILGVVQTCHSLGVMHRDLKPENFLFVNDDEDSPLKAIDFGLSMFLKPGENFTDVVGSPYYI

APEVLNKNYGPEADIWSAGVMIYVLLSGSAPFWGETEEEIFNEVLEGELDLTSDPWPQVSESAKDLIRKMLERNPIQRLTAQQVLCHPWIRDEGNAPDTP

LDTTVLSRLKKFSATDKLKKMALRVIAERLSEEEIHELRETFKTIDSGKSGRVTYKELKNGLERFNTNLDNSDINSLMQIPTDVHLEDTVDYNEFIEAIV

RLRQIQEEEANDRLESSTKV

>ATCPK20

MGNTCVGPNLNPNGFLQSVSAAVWRNQKPDDSIKSSKDESSRKKNDKSVNGDDSNGHVSSTVDPAPSTLPTPSTPPPPVKMANEEPPPKPITENKEDPNS

KPQKKEAHMKRMASAGLQIDSVLGRKTENLKDIYSVGRKLGQGQFGTTFLCVDKKTGKEFACKTIAKRKLTTPEDVEDVRREIQIMHHLSGHPNVIQIVG

AYEDAVAVHVVMEICAGGELFDRIIQRGHYTEKKAAELARIIVGVIEACHSLGVMHRDLKPENFLFVSGDEEAALKTIDFGLSVFFKPGETFTDVVGSPY

YVAPEVLRKHYSHECDVWSAGVIIYILLSGVPPFWDETEQGIFEQVLKGDLDFISEPWPSVSESAKDLVRRMLIRDPKKRMTTHEVLCHPWARVDGVALD

KPLDSAVLSRLQQFSAMNKLKKIAIKVIAESLSEEEIAGLKEMFKMIDTDNSGHITLEELKKGLDRVGADLKDSEILGLMQAADIDNSGTIDYGEFIAAM

VHLNKIEKEDHLFTAFSYFDQDGSGYITRDELQQACKQFGLADVHLDDILREVDKDNDGRIDYSEFVDMMQDTGFGKMGLKVS

>ATCPK14

MGNCCGTAGSLIQDKQKKGFKLPNPFSNEYGNHHDGLKLIVLKEPTGHEIKQKYKLGRELGRGEFGVTYLCTEIETGEIFACKSILKKKLKTSIDIEDVK

REVEIMRQMPEHPNIVTLKETYEDDKAVHLVMELCEGGELFDRIVARGHYTERAAASVIKTIIEVVQMCHKHGVMHRDLKPENFLFANKKETASLKAIDF

GLSVFFKPGERFNEIVGSPYYMAPEVLRRSYGQEIDIWSAGVILYILLCGVPPFWAETEHGVAKAILKSVIDFKRDPWPKVSDNAKDLIKKMLHPDPRRR

LTAQQVLDHPWIQNGKNASNVSLGETVRARLKQFSVMNKLKKRALRVIAEHLSVEETSCIKERFQVMDTSNRGKITITELGIGLQKLGIVVPQDDIQILM

DAGDVDKDGYLDVNEFVAISVHIRKLGNDEHLKKAFTFFDKNKSGYIEIEELRDALADDVDTTSEEVVEAIILDVDTNKDGKISYDEFATMMKTGTDWRK

ASRQYSRDLFKCLSLKLMQDGSLQSNGDTK

>ATCPK2

MGNACVGPNISGNGFLQTVTAAMWRPRIGAEQASSSSHGNGQVSKEAASEPATDQVQNKPPEPITMPSSKTNPETKLKPDLEIQPEEKKEKVLAEETKQK

VVPEESKQEVPPEESKREVVVQPESAKPETKSESKPETTKPETTSETKPETKAEPQKPKHMRRVSSAGLRTESVLQRKTENFKEFYSLGRKLGQGQFGTT

FLCLEKGTGNEYACKSISKRKLLTDEDVEDVRREIQIMHHLAGHPNVISIKGAYEDVVAVHLVMELCSGGELFDRIIQRGHYTERKAAELARTIVGVLEA

CHSLGVMHRDLKPENFLFVSREEDSLLKTIDFGLSMFFKPDEVFTDVVGSPYYVAPEVLRKRYGPESDVWSAGVIVYILLSGVPPFWAETEQGIFEQVLH

GDLDFSSDPWPSISESAKDLVRKMLVRDPKRRLTAHQVLCHPWVQIDGVAPDKPLDSAVLSRMKQFSAMNKFKKMALRVIAESLSEEEIAGLKQMFKMID

ADNSGQITFEELKAGLKRVGANLKESEILDLMQAADVDNSGTIDYKEFIAATLHLNKIEREDHLFAAFSYFDKDESGFITPDELQQACEEFGVEDARIEE

MMRDVDQDKDGRIDYNEFVAMMQKGSIMGGPVKMGLENSISISLKH

>ATCPK9

MGNCFAKNHGLMKPQQNGNTTRSVEVGVTNQDPPSYTPQARTTQQPEKPGSVNSQPPPWRAAAAAPGLSPKTTTKSNSILENAFEDVKLFYTLGKELGRG

QFGVTYLCTENSTGKKYACKSISKKKLVTKADKDDMRREIQIMQHLSGQPNIVEFKGAYEDEKAVNLVMELCAGGELFDRIIAKGHYTERAAASVCRQIV

NVVKICHFMGVLHRDLKPENFLLSSKDEKALIKATDFGLSVFIEEGKVYRDIVGSAYYVAPEVLRRRYGKEVDIWSAGIILYILLSGVPPFWAETEKGIF

DAILEGHIDFESQPWPSISSSAKDLVRRMLTADPKRRISAADVLQHPWLREGGEASDKPIDSAVLSRMKQFRAMNKLKKLALKVIAENIDTEEIQGLKAM

FANIDTDNSGTITYEELKEGLAKLGSKLTEAEVKQLMDAADVDGNGSIDYIEFITATMHRHRLESNENLYKAFQHFDKDSSGYITIDELESALKEYGMGD

DATIKEVLSDVDSDNDGRINYEEFCAMMRSGNPQQQQPRLF

>ATCPK13

MGNCCRSPAAVAREDVKSNYSGHDHARKDAAGGKKSAPIRVLSDVPKENIEDRYLLDRELGRGEFGVTYLCIERSSRDLLACKSISKRKLRTAVDIEDVK

REVAIMKHLPKSSSIVTLKEACEDDNAVHLVMELCEGGELFDRIVARGHYTERAAAGVTKTIVEVVQLCHKHGVIHRDLKPENFLFANKKENSPLKAIDF

GLSIFFKPGEKFSEIVGSPYYMAPEVLKRNYGPEIDIWSAGVILYILLCGVPPFWAESEQGVAQAILRGVIDFKREPWPNISETAKNLVRQMLEPDPKRR

LTAKQVLEHPWIQNAKKAPNVPLGDVVKSRLKQFSVMNRFKRKALRVIAEFLSTEEVEDIKVMFNKMDTDNDGIVSIEELKAGLRDFSTQLAESEVQMLI

EAVDTKGKGTLDYGEFVAVSLHLQKVANDEHLRKAFSYFDKDGNGYILPQELCDALKEDGGDDCVDVANDIFQEVDTDKDGRISYEEFAAMMKTGTDWRK

ASRHYSRGRFNSLSIKLMKDGSLNLGNE

>ATCPK32

MGNCCGTAGSLAQNDNKPKKGRKKQNPFSIDYGLHHGGGDGGGRPLKLIVLNDPTGREIESKYTLGRELGRGEFGVTYLCTDKETDDVFACKSILKKKLR

TAVDIEDVRREVEIMRHMPEHPNVVTLKETYEDEHAVHLVMELCEGGELFDRIVARGHYTERAAAAVTKTIMEVVQVCHKHGVMHRDLKPENFLFGNKKE

TAPLKAIDFGLSVFFKPGERFNEIVGSPYYMAPEVLKRNYGPEVDIWSAGVILYILLCGVPPFWAETEQGVAQAIIRSVLDFRRDPWPKVSENAKDLIRK

MLDPDQKRRLTAQQVLDHPWLQNAKTAPNVSLGETVRARLKQFTVMNKLKKRALRVIAEHLSDEEASGIREGFQIMDTSQRGKINIDELKIGLQKLGHAI

PQDDLQILMDAGDIDRDGYLDCDEFIAISVHLRKMGNDEHLKKAFAFFDQNNNGYIEIEELREALSDELGTSEEVVDAIIRDVDTDKDGRISYEEFVTMM

KTGTDWRKASRQYSRERFNSISLKLMQDASLQVNGDTR

>ATCPK31

MGCYSSKNLKQSKRTILEKPFVDIGKVYILGDELGQGQFGITRKCVEKTSGKTYACKTILKTNLKSREDEEAVKREIRIMKHLSGEPNIVEFKKAYEDRD

SVHIVMEYCGGGELFKKIEALSKDGKSYSEKEAVEIIRPIVNVVKNCHYMGVMLRDLKPENFLLSSTDKNATVKAIDFGCSVFIEEGEVHRKFAGSAYYI

APEVLQGKYGKEADIWSAGIILYILLCGKPPFVTEPEAQMFSEIKSAKIDVDSESWKFIDVKAKHLVNRMLNRNPKERISAAEVLGHPWMKDGEASDKPI

DGVVLSRLKQFRDMNKLKKVALKVIAANLSEEEIKGLKTLFTNIDTDKSGTITLEELKTGLTRLGSNLSKTEVEQLMEAADVDGNGTIDIDEFISATMHR

YRLDRDDHVYQAFQHFDKDNDGHITKEELEMAMKEHGVGDEVSIKQIITEVDTDNDGKINFEEFRTMMRSGSSLQPQRELLPIK

>ATCPK27

MGCFSSKELQQSKRTILEKPLVDITKIYILGEELGRGNFGLTRKCVEKSTGKTFACKTILKTKLKDEECEEDVKREIRIMKQLSGEPNIVEFKNAYEDKD

SVHIVMEYCGGGELYDKILALYDVGKSYSEKEAAGIIRSIVNVVKNCHYMGVMHRDLKPENFLLTSNDDNATVKVIDFGCSVFIEEGKVYQDLAGSDYYI

APEVLQGNYGKEADIWSAGIILYILLCGKSPFVKEPEGQMFNEIKSLEIDYSEEPWPLRDSRAIHLVKRMLDRNPKERISAAEVLGHPWMKEGEASDKPI

DGVVLSRLKRFRDANKFKKVVLKFIAANLSEEEIKGLKTLFTNIDTDKSGNITLEELKTGLTRLGSNLSKTEVEQLMEAADMDGNGTIDIDEFISATMHR

YKLDRDEHVYKAFQHFDKDNDGHITKEELEMAMKEDGAGDEGSIKQIIADADTDNDGKINFEEFRTMMRTESSLQPEGELLPIIN

>ATCPK22

MGNCCGSKPLTASDIVSDQKQETILGKPLEDIKKHYSFGDELGKGKSYACKSIPKRTLSSEEEKEAVKTEIQIMDHVSGQPNIVQIKGSYEDNNSIHIVM

ELCGGGELFDKIDALVKSHSYYSEKDAAGIFRSIVNAVKICHSLDVVHRDLKPENFLFSSKDENAMLKAIDFGCSVYIKEGKTFERVVGSKYYIAPEVLE

GSYGKEIDIWSAGVILYILLSGVPPFQTGIESIIVSTLCIVDAEIKECRLDFESQPWPLISFKAKHLIGKMLTKKPKERISAADVLEHPWMKSEAPDKPI

DNVVLSRMKQFRAMNKLKKLALKVIAEGLSEEEIKGLKTMFENMDMDKSGSITYEELKMGLNRHGSKLSETEVKQLMEAVSADVDGNGTIDYIEFISATM

HRHRLERDEHLYKAFQYFDKDGSGHITKEEVEIAMKEHGMGDEANAKDLISEFDKNNDGKIDYEEFCTMMRNGILQPQGKLLKRLYMNLEELKTGLTRLG

SRLSETEIDKAFQHFDKDNSGHITRDELESAMKEYGMGDEASIKEVISEVDTDNVSCTLQHIANISNIKQVLETL

>ATCPK21

MGCFSSKHRKTQNDGGEKSIPINPVQTHVVPEHRKPQTPTPKPMTQPIHQQISTPSSNPVSVRDPDTILGKPFEDIRKFYSLGKELGRGQFGITYMCKEI

GTGNTYACKSILKRKLISKQDKEDVKREIQIMQYLSGQPNIVEIKGAYEDRQSIHLVMELCAGGELFDRIIAQGHYSERAAAGIIRSIVNVVQICHFMGV

VHRDLKPENFLLSSKEENAMLKATDFGLSVFIEEGKVYRDIVGSAYYVAPEVLRRSYGKEIDIWSAGVILYILLSGVPPFWAENEKGIFDEVIKGEIDFV

SEPWPSISESAKDLVRKMLTKDPKRRITAAQVLEHPWIKGGEAPDKPIDSAVLSRMKQFRAMNKLKKLALKVIAESLSEEEIKGLKTMFANIDTDKSGTI

TYEELKTGLTRLGSRLSETEVKQLMEAADVDGNGTIDYYEFISATMHRYKLDRDEHVYKAFQHFDKDNSGHITRDELESAMKEYGMGDEASIKEVISEVD

TDNDGRINFEEFCAMMRSGSTQPQGKLLPFH

>ATCPK23

MGCFSSKHRKTQNDGGGERSIPIIPVQTHIVDQVPDHRKPQIPSPSIPISVRDPETILGKPFEDIRKFYSLGRELGRGGLGITYMCKEIGTGNIYACKSI

LKRKLISELGREDVKTEIQIMQHLSGQPNVVEIKGSYEDRHSVHLVMELCAGGELFDRIIAQGHYSERAAAGTIKSIVDVVQICHLNGVIHRDLKPENFL

FSSKEENAMLKVTDFGLSAFIEEGKIYKDVVGSPYYVAPEVLRQSYGKEIDIWSAGVILYILLCGVPPFWADNEEGVFVEILKCKIDFVREPWPSISDSA

KDLVEKMLTEDPKRRITAAQVLEHPWIKGGEAPEKPIDSTVLSRMKQFRAMNKLKKLALKVSAVSLSEEEIKGLKTLFANMDTNRSGTITYEQLQTGLSR

LRSRLSETEVQQLVEASDVDGNGTIDYYEFISATMHRYKLHHDEHVHKAFQHLDKDKNGHITRDELESAMKEYGMGDEASIKEVISEVDTDNALSPVEIM

REVALEIGVPVNTFKQNNVQEDGLYLPVLNNAA

>ATCPK4

MEKPNPRRPSNSVLPYETPRLRDHYLLGKKLGQGQFGTTYLCTEKSSSANYACKSIPKRKLVCREDYEDVWREIQIMHHLSEHPNVVRIKGTYEDSVFVH

IVMEVCEGGELFDRIVSKGCFSEREAAKLIKTILGVVEACHSLGVMHRDLKPENFLFDSPSDDAKLKATDFGLSVFYKPGQYLYDVVGSPYYVAPEVLKK

CYGPEIDVWSAGVILYILLSGVPPFWAETESGIFRQILQGKIDFKSDPWPTISEGAKDLIYKMLDRSPKKRISAHEALCHPWIVDEHAAPDKPLDPAVLS

RLKQFSQMNKIKKMALRVIAERLSEEEIGGLKELFKMIDTDNSGTITFEELKAGLKRVGSELMESEIKSLMDAADIDNSGTIDYGEFLAATLHINKMERE

ENLVVAFSYFDKDGSGYITIDELQQACTEFGLCDTPLDDMIKEIDLDNDGKIDFSEFTAMMKKGDGVGRSRTMRNNLNFNIAEAFGVEDTSSTAKSDDSP

K

>ATCPK15

MGCFSSKHRNTESDIINGSVQSSIPTNQPENHVSRDVLKPQKPPSPQIPTTTQSNHHHQQESKPVNQQIEKKHVLTQPLKPIVFRETETILGKPFEEIRK

LYTLGKELGRGQFGITYTCKENSTGNTYACKSILKRKLTRKQDIDDVKREIQIMQYLSGQENIVEIKGAYEDRQSIHLVMELCGGSELFDRIIAQGHYSE

KAAAGVIRSVLNVVQICHFMGVIHRDLKPENFLLASTDENAMLKATDFGLSVFIEEGKVYRDIVGSAYYVAPEVLRRSYGKEIDIWSAGIILYILLCGVP

PFWSETEKGIFNEIIKGEIDFDSQPWPSISESAKDLVRKLLTKDPKQRISAAQALEHPWIRGGEAPDKPIDSAVLSRMKQFRAMNKLKKLALKVIAESLS

EEEIKGLKTMFANMDTDKSGTITYEELKNGLAKLGSKLTEAEVKQLMEAADVDGNGTIDYIEFISATMHRYRFDRDEHVFKAFQYFDKDNSGFITMDELE

SAMKEYGMGDEASIKEVIAEVDTDNDGRINYEEFCAMMRSGITLPQQGKILPPCKRVADLN

>ATCPK3

MGHRHSKSKSSDPPPSSSSSSSGNVVHHVKPAGERRGSSGSGTVGSSGSGTGGSRSTTSTQQNGRILGRPMEEVRRTYEFGRELGRGQFGVTYLVTHKET

KQQVACKSIPTRRLVHKDDIEDVRREVQIMHHLSGHRNIVDLKGAYEDRHSVNLIMELCEGGELFDRIISKGLYSERAAADLCRQMVMVVHSCHSMGVMH

RDLKPENFLFLSKDENSPLKATDFGLSVFFKPGDKFKDLVGSAYYVAPEVLKRNYGPEADIWSAGVILYILLSGVPPFWGENETGIFDAILQGQLDFSAD

PWPALSDGAKDLVRKMLKYDPKDRLTAAEVLNHPWIREDGEASDKPLDNAVLSRMKQFRAMNKLKKMALKVIAENLSEEEIIGLKEMFKSLDTDNNGIVT

LEELRTGLPKLGSKISEAEIRQLMEAADMDGDGSIDYLEFISATMHMNRIEREDHLYTAFQFFDNDNSGYITMEELELAMKKYNMGDDKSIKEIIAEVDT

DRDGKINYEEFVAMMKKGNPELVPNRRRM

>ATCPK5

MGNSCRGSFKDKLDEGDNNKPEDYSKTSTTNLSSNSDHSPNAADIIAQEFSKDNNSNNNSKDPALVIPLREPIMRRNPDNQAYYVLGHKTPNIRDIYTLS

RKLGQGQFGTTYLCTEIASGVDYACKSISKRKLISKEDVEDVRREIQIMHHLAGHGSIVTIKGAYEDSLYVHIVMELCAGGELFDRIIQRGHYSERKAAE

LTKIIVGVVEACHSLGVMHRDLKPENFLLVNKDDDFSLKAIDFGLSVFFKPGQIFTDVVGSPYYVAPEVLLKRYGPEADVWTAGVILYILLSGVPPFWAE

TQQGIFDAVLKGYIDFESDPWPVISDSAKDLIRRMLSSKPAERLTAHEVLRHPWICENGVAPDRALDPAVLSRLKQFSAMNKLKKMALKVIAESLSEEEI

AGLREMFQAMDTDNSGAITFDELKAGLRKYGSTLKDTEIHDLMDAADVDNSGTIDYSEFIAATIHLNKLEREEHLVAAFQYFDKDGSGFITIDELQQACV

EHGMADVFLEDIIKEVDQNNDGKIDYGEFVEMMQKGNAGVGRRTMRNSLNISMRDA

>ATCPK18

MGLCFSSPKATRRGTGSRNPNPDSPTQGKASEKVSNKNKKNTKKIQLRHQGGIPYGKRIDFGYAKDFDNRYTIGKLLGHGQFGFTYVATDNNNGNRVAVK

RIDKAKMTQPIEVEDVKREVKILQALGGHENVVGFHNAFEDKTYIYIVMELCDGGELLDRILAKKDSRYTEKDAAVVVRQMLKVAAECHLRGLVHRDMKP

ENFLFKSTEEGSSLKATDFGLSDFIKPGVKFQDIVGSAYYVAPEVLKRRSGPESDVWSIGVITYILLCGRRPFWDKTQDGIFNEVMRKKPDFREVPWPTI

SNGAKDFVKKLLVKEPRARLTAAQALSHSWVKEGGEASEVPIDISVLNNMRQFVKFSRLKQIALRALAKTINEDELDDLRDQFDAIDIDKNGSISLEEMR

QALAKDVPWKLKDARVAEILQANDSNTDGLVDFTEFVVAALHVNQLEEHDSEKWQQRSRAAFDKFDIDGDGFITPEELRLQTGLKGSIEPLLEEADVDED

GRISINEFRRLLRSASLKSKNVKSPPGTEHIICHNLLDGICIEDTEERTSAVRFEYVSQVL

>ATCPK26

MGLALFSSDGKLIWKGSTQTGKRRPQEEATMKHSGGNQACYVLGQKTPSIRDLYSLGHKLGQGQFGTTYMCKEISTGREYACKSITKRKLISKEDVEDVR

REIQIMHHLAGYKNIVTIKGAYEDPLYVHIVMELCSGGELFDRIIQRGHYSERKAAELIKIIVGVVEACHSLGVMHRDLKPENFLLVNKDDDFSLKAIDF

GLSVFFKPGQIFEDVVGSPYYVAPEVLLKHYGPEADVWTAGVILYILVSGVPPFWAETQQGIFDAVLKGHIDFDSDPWPLISDSAKNLIRGMLCSRPSER

LTAHQVLRHPWICENGVAPDRALDPAVLSRLKQFSAMNKLKQMALRVIAESLSEEEIAGLKEMFKAMDTDNSGAITFDELKAGLRRYGSTLKDTEIRDLM

EAADIDKSGTIDYGEFIAATIHLNKLEREEHLLSAFRYFDKDGSGYITIDELQHACAEQGMSDVFLEDVIKEVDQDNDGRIDYGEFVAMMQKGIVGRTMR

KSINMSIRNNAVSQ

>ATCPK1

MGNTCVGPSRNGFLQSVSAAMWRPRDGDDSASMSNGDIASEAVSGELRSRLSDEVQNKPPEQVTMPKPGTDVETKDREIRTESKPETLEEISLESKPETK

QETKSETKPESKPDPPAKPKKPKHMKRVSSAGLRTESVLQRKTENFKEFYSLGRKLGQGQFGTTFLCVEKTTGKEFACKSIAKRKLLTDEDVEDVRREIQ

IMHHLAGHPNVISIKGAYEDVVAVHLVMECCAGGELFDRIIQRGHYTERKAAELTRTIVGVVEACHSLGVMHRDLKPENFLFVSKHEDSLLKTIDFGLSM

FFKPDDVFTDVVGSPYYVAPEVLRKRYGPEADVWSAGVIVYILLSGVPPFWAETEQGIFEQVLHGDLDFSSDPWPSISESAKDLVRKMLVRDPKKRLTAH

QVLCHPWVQVDGVAPDKPLDSAVLSRMKQFSAMNKFKKMALRVIAESLSEEEIAGLKEMFNMIDADKSGQITFEELKAGLKRVGANLKESEILDLMQAAD

VDNSGTIDYKEFIAATLHLNKIEREDHLFAAFTYFDKDGSGYITPDELQQACEEFGVEDVRIEELMRDVDQDNDGRIDYNEFVAMMQKGSITGGPVKMGL

EKSFSIALKL

>ATCPK17

MGNCCSHGRDSADNGDALENGASASNAANSTGPTAEASVPQSKHAPPSPPPATKQGPIGPVLGRPMEDVKASYSLGKELGRGQFGVTHLCTQKATGHQFA

CKTIAKRKLVNKEDIEDVRREVQIMHHLTGQPNIVELKGAYEDKHSVHLVMELCAGGELFDRIIAKGHYSERAAASLLRTIVQIVHTCHSMGVIHRDLKP

ENFLLLNKDENSPLKATDFGLSVFYKPGEVFKDIVGSAYYIAPEVLKRKYGPEADIWSIGVMLYILLCGVPPFWAESENGIFNAILRGHVDFSSDPWPSI

SPQAKDLVKKMLNSDPKQRLTAAQVLNHPWIKEDGEAPDVPLDNAVMSRLKQFKAMNNFKKVALRVIAGCLSEEEIMGLKEMFKGMDTDSSGTITLEELR

QGLAKQGTRLSEYEVQQLMEAADADGNGTIDYGEFIAATMHINRLDREEHLYSAFQHFDKDNSGYITMEELEQALREFGMNDGRDIKEIISEVDGDNDGR

INYDEFVAMMRKGNPDPIPKKRRELSFK

>ATCPK7

MGNCCGNPSSATNQSKQGKPKNKNNPFYSNEYATTDRSGAGFKLSVLKDPTGHDISLQYDLGREVGRGEFGITYLCTDKETGEKYACKSISKKKLRTAVD

IEDVRREVEIMKHMPKHPNVVSLKDSFEDDDAVHIVMELCEGGELFDRIVARGHYTERAAAAVMKTIVEVVQICHKQGVMHRDLKPENFLFANKKETSAL

KAIDFGLSVFFKPGEQFNEIVGSPYYMAPEVLRRNYGPEIDVWSAGVILYILLCGVPPFWAETEQGVAQAIIRSVIDFKRDPWPRVSDSAKDLVRKMLEP

DPKKRLTAAQVLEHTWILNAKKAPNVSLGETVKARLKQFSVMNKLKKRALRVIAEHLSVEEAAGIKEAFEMMDVNKRGKINLEELKYGLQKAGQQIADTD

LQILMEATDVDGDGTLNYSEFVAVSVHLKKMANDEHLHKAFNFFDQNQSGYIEIDELREALNDELDNTSSEEVIAAIMQDVDTDKDGRISYEEFVAMMKA

GTDWRKASRQYSRERFNSLSLKLMRDGSLQLEGET

>ATCPK34

MGNCCSHGRDSDDNKEEPRPENGGGGVGAAEASVRASKHPPASPPPATKQGPIGPVLGRPMEDVKSSYTLGKELGRGQFGVTHLCTQKATGLQFACKTIA

KRKLVNKEDIEDVRREVQIMHHLTGQPNIVELKGAYEDKHSVHLVMELCAGGELFDRIIAKGHYSERAAASLLRTIVQIIHTCHSMGVIHRDLKPENFLL

LSKDENSPLKATDFGLSVFYKPGEVFKDIVGSAYYIAPEVLRRKYGPEADIWSIGVMLYILLCGVPPFWAESENGIFNAILSGQVDFSSDPWPVISPQAK

DLVRKMLNSDPKQRLTAAQVLNHPWIKEDGEAPDVPLDNAVMSRLKQFKAMNNFKKVALRVIAGCLSEEEIMGLKEMFKGMDTDNSGTITLEELRQGLAK

QGTRLSEYEVQQLMEAADADGNGTIDYGEFIAATMHINRLDREEHLYSAFQHFDKDNSGYITTEELEQALREFGMNDGRDIKEIISEVDGDNDGRINYEE

FVAMMRKGNPDPNPKKRRELSFK

>ATCPK8

MGNCCASPGSETGSKKGKPKIKSNPFYSEAYTTNGSGTGFKLSVLKDPTGHDISLMYDLGREVGRGEFGITYLCTDIKTGEKYACKSISKKKLRTAVDIE

DVRREVEIMKHMPRHPNIVSLKDAFEDDDAVHIVMELCEGGELFDRIVARGHYTERAAAAVMKTILEVVQICHKHGVMHRDLKPENFLFANKKETSALKA

IDFGLSVFFKPGEGFNEIVGSPYYMAPEVLRRNYGPEVDIWSAGVILYILLCGVPPFWAETEQGVAQAIIRSVIDFKRDPWPRVSETAKDLVRKMLEPDP

KKRLSAAQVLEHSWIQNAKKAPNVSLGETVKARLKQFSVMNKLKKRALRVIAEHLSVEEVAGIKEAFEMMDSKKTGKINLEELKFGLHKLGQQQIPDTDL

QILMEAADVDGDGTLNYGEFVAVSVHLKKMANDEHLHKAFSFFDQNQSDYIEIEELREALNDEVDTNSEEVVAAIMQDVDTDKDGRISYEEFAAMMKAGT

DWRKASRQYSRERFNSLSLKLMREGSLQLEGEN

>ATCPK12

MANKPRTRWVLPYKTKNVEDNYFLGQVLGQGQFGTTFLCTHKQTGQKLACKSIPKRKLLCQEDYDDVLREIQIMHHLSEYPNVVRIESAYEDTKNVHLVM

ELCEGGELFDRIVKRGHYSEREAAKLIKTIVGVVEACHSLGVVHRDLKPENFLFSSSDEDASLKSTDFGLSVFCTPGEAFSELVGSAYYVAPEVLHKHYG

PECDVWSAGVILYILLCGFPPFWAESEIGIFRKILQGKLEFEINPWPSISESAKDLIKKMLESNPKKRLTAHQVLCHPWIVDDKVAPDKPLDCAVVSRLK

KFSAMNKLKKMALRVIAERLSEEEIGGLKELFKMIDTDKSGTITFEELKDSMRRVGSELMESEIQELLRAADVDESGTIDYGEFLAATIHLNKLEREENL

VAAFSFFDKDASGYITIEELQQAWKEFGINDSNLDEMIKDIDQDNDGQIDYGEFVAMMRKGNGTGGGIGRRTMRNSLNFGTTLPDESMNV

>ATCPK28

MGVCFSAIRVTGASSSRRSSQTKSKAAPTPIDTKASTKRRTGSIPCGKRTDFGYSKDFHDHYTIGKLLGHGQFGYTYVAIHRPNGDRVAVKRLDKSKMVL

PIAVEDVKREVQILIALSGHENVVQFHNAFEDDDYVYIVMELCEGGELLDRILSKKGNRYSEKDAAVVVRQMLKVAGECHLHGLVHRDMKPENFLFKSAQ

LDSPLKATDFGLSDFIKPGKRFHDIVGSAYYVAPEVLKRRSGPESDVWSIGVITYILLCGRRPFWDRTEDGIFKEVLRNKPDFSRKPWATISDSAKDFVK

KLLVKDPRARLTAAQALSHAWVREGGNATDIPVDISVLNNLRQFVRYSRLKQFALRALASTLDEAEISDLRDQFDAIDVDKNGVISLEEMRQALAKDLPW

KLKDSRVAEILEAIDSNTDGLVDFTEFVAAALHVHQLEEHDSEKWQLRSRAAFEKFDLDKDGYITPEELRMHTGLRGSIDPLLDEADIDRDGKISLHEFR

RLLRTASISSQRAPSPAGHRNLR

>GhCPK1

MGTCLTKSKDSKPTHNGYGSGPTTTAAVHQQRYQEPVRPAPVQPQFHHIPEKPGTQTPWK

PVAPSPSPKPVAPRVDTILGKPFEDIRMHYTIGKELGKGQFGVTYLCIENSTGKQYACKT

ISKKKLVTRNDKEDMRREIQIMQHLSGQPNIVEFKGAYEDKLSVHLVMELCAGGELFDRI

IAKGHYSERAAASICRAIVNVVHACHFMGVMHRDLKPENFLLSSKGENALLKATDFGLSV

FIEDGKVYKDIVGSAYYVAPEVLLRKYGKEIDIWSAGVILYILLSGVPPFWAETEKRIFD

AILEGEIDFESQPWPSISESAKDLVRQMLTQDPKKRITSTQVLEHPWIREGGSASDKPID

SAVLSRMKQFRRMNKLKQLALKVIAENLSSEEIQGLKQMFANIDTDNSGTITYEELKTGL

ARLGSKLTEAEVQQLMEAADVDGNGSIDYIEFITATMHRHRLERDEHLYKAFQHFDKDNS

GYITRDELEAAMKEYGMGDDDTIKEIISEVDTDNDGKINYEEFRDMMRSGTQHTQLF

>GhCPK2

MGNSCAKSAATEEDENEDNKQDGNEGEAKDPGQSESKEPEESGSVRKQPPEEMKIVREED

KTGKEQESKSTEKAAEMTQRQQSKSHPQRLNSKPSQLAGFNKEGSKTGNKTRKAHNVKRQ

SCAGLQVGSVLQTKTGHLKEYYNLGRKLGQGQFGTTFLCIEKGTGKEYACKSIAKRKLTT

TEDVDDVKREIQIMHHLAGHPNVISIKGSYEDNMAVHVVMELCAGGELFDRIVKRGHYSE

RKAAEIARIIVAVVEACHSMGVMHRDLKPENFLFVNNEEDSPLKAIDFGLSIFFKPGDIL

NDVVGSPYYVAPEVLRKHYGPEADVWSAGVITYILLSGVPPFWGETEQEIFNEVLNGELD

FSSDPWPNISESAKDLVTKMLDRDTKRRIKAHEVLRHPWVQVDGVAPDKPLDSVVLSRMK

QFSAMDKLKKMALRVIAQRLSEEEIAGLKEMFKMIDTDNSGQITYDELKEGLKRFGANLA

ESEFRALMQAADINNSGTIDYEEFVTATLHLNKIEREDNLLAAFSYFDRDSSGYITLDEL

QKACQEFGIQDIHLDEIMREVDQDNDGRIDYNEFVAMMQEGNPKLGKKGK

>GhCPK3

MGSCVARPSKLIGVRKGDYPKSKSSRPYFNSKCSRKSKSLRTSNNKVLKNPLEENILHKY

EIGNELGRGEFGITYQCFELETGEAYACKKISKAKLKTDIDIEDVQREVEILRLLPKHPN

LVSYKDAFEDDEAVYLVMELCRGGELFDRIVAKGHYTERAAAKVIKTILEIVKVCHEHGV

IHRDLKPENFLLADESETAPIKVIDFGLSIFYEPGERFSDIVGSPYYMAPEVLRRNYGKE

IDIWSTGVILYILLCGVPPFWADTEEGIARAIIRGVIDFERDPWPKVSAEVKDLVRSMLD

PNPYTRISLQEVLEHPWIQNLQNAPNFNLGENVGARIKQFSLMGKFKKKVLRVVADNLPN

EQIDVIIEMFNMMDTDENGYLSFEELRDGLQKMGHSVGDPAVRMLMEAADIDGNGTLSCE

EFVIMVVHLKRIGNDEHLAQAFNHFDKNQSGYIEFEELKETLMQDDPGPNNEQLIKDIMQ

DVDKDKDGRISYQEFKAMMLTGMDWKMASRQYSRALINAVSIKILRQSGQLK

>GhCPK4

MNNQSSSIPTTAATKGWVLPYETPRLRDHYVVGKKLGQGQFGTTYECIHKATGTVFACKS

IPKRKLLCREDYDDVWREIKIMHHLSEHPSVVRIEGTYEDSVFVHLVMEICLGGELFDRI

VAKGHYSEREAAKLIKTIVGVVEACHSLGVMHRDLKPENFLFDSPGDDAVLKATDFGLSI

FYKPGQRYADVVGSPFYVAPEVLCKHYGPEIDIWSAGVILYILLSGVPPFWAETESGIFR

QILHGKLDFTSEPWPSISESAKDLIRKMLERHPKSRISAYQVLCHPWIVDDRVAPDKPLD

SAVLSRLKQFSAMNKLKKMALRVIAERLSEEEIGGLKELFKMIDTDSSGTITYQELKDGL

KKVGSELMESEIKSLMEAADIDNNGTIDYGEFIAATLHMNKLEREENIVAAFTFFDKDGS

GYITVDELQQACKEFGLGDVHLEDMIKEIDQDNDGRIDYGEFAAMMRKGDGLGRSRSMRS

NLNKNIADAFGLGVKDLTSISSDSNSCPSSS

>GhCPK5

MGNCNGLPSTGNQFQPLSDSGVGLLDGGINVQPAPPPPRPQQSSTHHHPSPAVGRVLGRP

MEDVRSTYVFSGELGRGQFGITYLVTHKKTKQQFACKSIPKRKLINLEDIEDVRREVQIM

YHLTGHRNIVELKGAYEDHQSVNLIMELCAGGELFNRIIAKGHFSERKAANLCRQIVMML

HNCHSMGVMHRDLKPENFLFLNKDEDSPLKATDFGLSVFFKPGDVFKDLVGSAYYVAPEV

LRRHYGPEADIWSAGVILYVLLSGVPPFYGETEQSIFDSILRGNIDFSSDPWPSISSSAK

DLVRKMLRDDPKERLSASEVLNHQWMREDGDASDKPLDIAVLTRMKQFSAMNKLKKVALK

VIAENLSEEEIIGLKEMFKSMDTDNSGTISFEELKMGLPKLGTKLSESEVRQLMEAADFD

GNGAIDYIEFITATMHMNRTEREEHLYTAFQYFDEDNSGFITMEELEQALRKYNMGDEKT

IKEIIAEVDTDRDGRINYDEFVAMMRKGNPELVASRRCK

>GhCPK6

MGACLSATKVSGGSSGNTTAHHRKTATNAEKESQKPNNQQVRCSQPLKNKQKPKKQSGII

PWGKRTDFGYDKDFDQRYTIGKLLGHGQFGYTYVAIDKVNGDRVAVKKIDKNKMILPIAV

EDVKREVKILKALKGHENVVQFYNAFEDDSYVYIVMELCEGGELLDRILAKKDSRYSEKD

AAVVVRQMLKVAAECHLHGLVHRDMKPENFLFKSTRPDSPLKATDFGLSDFIKPGKRFRD

IVGSAYYVAPEVLKRRSGPESDVWSIGVITYILLCGKRPFWDKTEDGIFKEVLKNKPDFR

RKPWPTISNDAKDFLKKILVKDPRARLTAAQALSHQWVREGGNASDIPVDISVLSNLRQF

VKYSRLKQFALRALASTLNEEEIADLRDQFDAIDVDKNSSISLEEMRQALAKDLPWKLKD

SRVLEILQAIDSNMDGLVDFTEFVAAALHVNQMEEHDCDKWQMQLQAAFEKFDVDRDGYI

TPEELRMHTGLRGSIDPLLEEADIDKDGKISLSEFRRLFRTASMGSRNVSSPSGNRNTQK

L

>GhCPK7

MVSNNKKNQKRSYIKLLGYFMGNCCATPSTTASHEKKEKKGKKKQNPFSLDYGQHDHGNR

GHKLTVLNDPTGREIEPRYELGCELGRGEFGITYLCTDKETGDTFACKSISKKKLRTDVD

IEDVRREVEIMKHLPHHPNIVTLKDTYEDDNSVHLVMELCEGGELFDRIVARGHYTERAA

AAVTKTIVEVVQMCHKHGVMHRDLKPENFLFANKKETAALKSIDFGLSVFFKPGEIFTEI

VGSPYYMAPEVLKRNYGPEVDVWSAGVILYILLCGVPPFWAETEQGVAQAIFRSVIDFKR

DPWPKVSDNAKDLVRKMLNPDPKQRLTAQEVLDHPWLQNAKKAPNVSLGETVKARLKQFS

VMNKLKKRALKVIAEHLSVEEVAGIKEGFQLMDTANRGKINIDELRVGLHKLGHTIPDAD

LQILMEAGDVDKDGYLDYGEFVAISVHLRKMGNDEHLKKAFEFFDRNQSGYIEIEELRDA

LSDEVETNSEEVISAIMHDVDTDKDGRISYDEFAVMMKAGTDWRKASRQYSRERFNNLSL

KLMMDGSLQMNSEPR

>GhCPK8

MGNTCRGSLKGKLHKGDNQPKDHCSSRNNTSSGRSTTTTDYSPSTLNSQQLIAQEFSKET

NQKETHFPVINPTKKDNNNNTMRRGIDHQAYYVLGHKTPNIRDLYTLGRKLGQGQFGTTY

LCTEISTGTEYACKSISKRKLISNEDVEDVRREIQIMHHLAGHKNIVTIKGAYEDTLYVH

IVMELCSGGELFDRIIQRGHYSERKAAELTKIIVGVVEACHSLGVMHRDLKPENFLLVNK

DDDFSLKAIDFGLSVFFKPGQVFTDVVGSPYYVAPEVLLKHYGPEADVWTAGVILYILLS

GVPPFWAETQQGIFDAVLKGHIDFDSDPWPLISDSAKDLIRKMLCSRPSERLTAHEVLCH

PWICENGVAPDRALDPAVLSRLKQFSAMNKLKKMALRVIAESLSEEEIAGLREMFTSMDT

DNSGAITFDELKAGLRRYGSTLKDTEIRDLMDAADVDNSGTIDYGEFIAATVHLNKLERE

EHLVAAFRYFDKDGSGYITVDELQQACAEHNMTDVLLEDIIREVDQDNDGRIDYGEFVAM

MQKGNAGIGRRTMRNSVNISMRDAPGAM

>GhCPK9

MGGCLTKTKGSNPQHNNGYKSGATTTTAAEQPQVTHIPEKPGTQAPWKPVVPTPSAKTAP

KSDTILGKPYEDIRMHYTIGKELGKGQFGVTYLCIENSTGKQYACKTISKRKLITKNDKE

DMRREIQLMQHLSGQPNIVEFKGAYEDKLSVHLVMELCAGGELFDRIIAKGHYSERAAAS

MCRAIVNVVHACHFMGVMHRDLKPENFLLSSKDENALLKATDFGLSVFIEEGKVYRDIVG

SAYYVAPEVLKRKYGKEIDIWSAGVILYILLSGVPPFWAETEKGIFDAILEGEIDFESQP

WPSISDSAKDLVRKMLTPDPKKRITSTQALEHPWIREDGNASDKPLDNAVLSRMKQFRRM

NKLKQLALKVIAENLSTEEIQGLKQMFANIDTDNSGTITYDELKNGLARLGSKLTEAEVK

QLMEAADVDGNGSIDYIEFITATMHRHRLERDEHLYKAFQHFDKDNSGHITRDELEAAMK

EYGMGDDDTIKEIISEVDTDNDGKINYEEFRAMMRSGTQQGQLF

>GhCPK10

MGNACAGPSNLGGNGFFQSVTQAVWRQRPPIQDQLPAANEDNSNKGSDESNKSKGSDDCQ

DSAATENTAPVPVKIPNAEPKNKEGNNANKASGQKPNNLKRLTSTELESVLGRKTGNMKE

LYSLGRKLGQGQFGTTFFCVEKSTAKEFACKSIAKRKLTSTDDLEDVRREVHIMHHLAGH

PNVIQIIGAYEDAVAIYLVMEFCAGGELFDRIIQRGHYTERKAAALARVMVGVVQACHSL

GVMHRDLKPENFLFINKDEDAPLKAIDFGLSMFFKPGEIFNDVVGSPYYVAPEVLQKHYG

PECDVWSAGVIIYILLCGVPPFWDETEQGIFEQVMRGELDFTSEPWPSISNSAKDLVRRM

LIKDPKRRLTAHEVLCHPWVQEDGIAPDKPLDSAVLSRLKQFSAMNKLKKIAIRVIADSL

SEEEIAGLKEMFKMIDADNSGNITLEELKIGLEKVGSKLKDSEINGLMQAADIDNSGTID

YSEFIAAMLHLNKIQKEDHLFAAFNYFDKDGSGYITPDELQKACEQFGLQDVHLEDVIRE

VDQDNDGRIDYSEFVAMMQDTGLTGKHKTFKH

>GhCPK11

MGNCCRSPAAVAREDVKSNFSGHDHGRKDSVSKQKPPITVLNGVSKENIEEKYLVDRELG

RGEFGVTYLCIDRGTRELLACKSISKRKLRTAVDIEDVRREVAIMKHLPKNSSIVSLKEA

CEDDNAVHLVMELCEGGELFDRIVARGHYTERAAAAVTRTIVEVVQLCHKHGVIHRDLKP

ENFLFANKKENSPLKAIDFGLSIFFKPGERFSEIVGSPYYMAPEVLKRNYGPEIDIWSAG

VILYILLCGVPPFWAESEQGVAQAILRGLIDFKRDPWPNISESAKSLVRQMLEPDPKLRL

TAKQVLEHPWLQNAKKAPNVPLGDVVKSRLKQFSMMNRFKRKALRVIAEFLSVEEVEDIK

VMFNKMDTDNDGIVSVEELKAGFKNYGSQLAEPEVQMLIEAVDANGKGTLDYGEFLAVSL

HLQRMANDEHLRKAFSYFDKDGNGFIEPDELRDALMEDGADDCTNVANDIFQEVDTDKDG

RISYDEFAAMMKTGTDWRKASRHYSRGRFNSLSIKLMKDGSLNLGNE

>GhCPK12

MGNVCATSGICSVCASCCGPKPAEEGQKKTDNEGEKSETKEAEAGAAAADGAEVQKDPPE

EMKIVKEEKGQGEETLSQRQKSKVTQRQQSSMTRQQSMSMSTPMQRQQSTLQRQQSKVTQ

RQQSKAPPQRLESKPSQVAVAAATAAVAGKEEAKADAAKPRKPHNVKRQSCAGLKVDAVL

QTKTGNLKEYYNLGKKLGQGQFGTTFLCVEKGTGKEYACKSIAKRKLVTPEDVDDVKREI

QIMHHLAGHPNVVTIKEAYEDSVAVHVVMELCAGGELFDRIVQRGHYSERKAAELARTIV

GVVEACHSMGVMHRDLKPENFLFVNEQEDSPLQAIDFGLSIFFKPGEVLSDVVGSPYYVA

PEVLQKHYGPEADVWSAGVIVYILLSGVPPFWGETEQEIFEEVLHGELDFTSDPWPNISE

SAKDLVKKMLVRDAKKRITAHEVLRHPWVQVDGVAPDKPLDSVVLSRMKQFSAMNKLKKM

ALKVIAQRLSEEEIAGLKEMFKIIDTDNSGQITYDELKDGLKRFGADLDESEFRALMQAA

DVNNNGTIDYEEFVAATLHLNKIEREDNLMAAFSYFDKDGSGYITQDELQKACQEFGIEE

IHLDEMIGEVDQDNDGRIDYNEFVAMMQKGNPDLGKKTLGIKEALPPS

>GhCPK13

MGNTCRGPSKGNLCKGYNTSSHCSSSNNNNNNPPSKPLIPRESGKETGENDNHSASIRAS

EQESIMKLGNDNQTYFVMGHKTPNIRDLYTLGPKLGQGQFGTTYLCTELSTGIEYACKSI

SKTKLVAKEDVDDVRREIQIMYHLAGHKNIVTIKGTYEDSLYVHIVMELCSGGELFDRII

QRGHYTERKAAELTRIIVGVVEACHSLGVMHRDLKPENFLLVNKDDDFSLKAIDFGLSVF

FKPGQVFTDVVGSPYYVAPEVLQKHYGPEADVWTAGVILYILLSGVPPFWGETQQGIFDA

VLKGFIDFDSEPWPLISDSAKDLIRKMLCSRPSERLTAHKVLCHPWICENGVAPDKVLDP

AILSRLKQFSAMNKLKKLALRVIAESLSEEEIAGLREMFKSMDTDNSGAITFDELKAGLR

RYGSTLKDAEIRALMDAADIDNSGTIDYGEFIAATVHLNKLEHEEHLVAAFQYFDKDRSG

YITVDELQQAWAEYNLTDVLFEDIIREVDQDNDGRIDYAAHIMYRLYSKAKKKMVGGKAL

KQTRLWC

>GhCPK14

MGNLCSRSDPAANPDEKGEPGPENELNTSTSMNEDSPNSPPKASPTQSISSKPSNKPNPI

GPVLGRPMEDIKTTYNIGKELGRGQFGVTHLCTNKSTGEQFACKTIAKRKLANKEDIEDV

RREVQIMHHLTGQSNIVELKGAFEDKHSVHLVMELCAGGELFDRIIAKGHYTERAAASLL

RTVVQIVHTCHSMGVIHRDLKPENFLLLNKDEDSPLKATDFGLSVFYKPGEEFKEIVGSA

YYIAPEVLKRKYGPEADIWSIGVMLYIFLSGVPPFWAESENGIFNSILRGHIDFSSDPWP

SISPQAKELVKKMLNSDPKQRLTAVQVLSHPWIKEDGEAPDTPLDNAVISRLKQFKAMNN

FKKVALRVIAGCLSEEEIMGLKEMFKGMDTDNSGTITLEELKQGLSKQGTKLTEYEVQQL

MEAADADGNGTIDYDEFITATVHMNRMDREDHLYHAFQHFDKDNSGYITNEELEQALREH

GMHDANIKEIVSEVDSDNDGRINYDEFVAMMRKGNPEAHTKKRRELSVNIET

>GhCPK16

MGICQSLCRCFSKSHEIPVSSSSDSPPLPYQPLTVSTSGGQNPSFSKAPSSSQTGTILLK

PYVDITSLYDLRKELGRGQFGITYLCIEKATKREYACKSISRRKLTTDKDVGDVRREISI

LQHLTGQPNIVEFKGAYEDARNLHLVMELCSGGELFDRITAKRSYSERQASSICRQIMNV

VHACHFMGVMHRDLKPENFLMVSKDEDSQIKATDFGLSVFIEEGRMYKDLVGSPYYVAPE

VLQRKYGKEIDVWSAGVILYILLSGVPPFWGETDKEIFKAVSEGNLDLKSQPWPTISEGA

KDLIRKMLARDPKKRITAAQALEHPWMKEGGEASDKPIDSAVLSRLKQFRVMNKLKKLAL

KVIAENLSSEEEKKGLQQMFNNIDTDGSGTITLEELRDGLARLGSKLTEPEIKQLMDAAD

VDKSGTIDYIEFVTATMHRHRLDREDNIRKAFNYFDKDSNGFITRDELRQAMTQYGMGDE

ATIDEVIEDVDTDKDGRINYEEFVAMMKRGTQDGDGMAISISTSAAKHEEPIRTSYAGVQ

LEETVDETKQGKLRLDSWISSRIQGISRARVQSSIKSGLVKVNGRVVDKVSHSLRAGDKV

NCVISDLQPLKAEPEDIPLDIVFEDDHVLVVNKPSHMVVHPAPGNANGTLVNGILHHCSL

PTVASSEKEVLFDTEDMSDDEQDIFHGASAGPASVRPGIVHRLDKGTSGLLVVAKDEHSH

AHLSEQFKQHTIQRVYISLTCGVPSASAGRVDIPIGRDSNNRIRMVAVPGLSHHGRARHA

ASRYKVIEVLAGGGSALVQWRLETGRTHQIRAHAKYMGIPLLGDEVYGGTKNMALSLLRP

RTPPYYNDELSRLVSRLERPYLHALVLGFEHPHSGEKMRFSCPPPPDFGEILNHLRKIGI

EKPISKE

>GhCPK15

MNKKIAGSSSRPRKPTGTVLPYQTQRIRDHYFLGKKLGQGQFGTTYLCTHKVTGIRYACK

SIPKRKLVCREDYDDVWREIQIMHHLSENPFVVQIKGTYEDAVFVHLVMELCAGGELFDR

IVAKGHYSEREAAKLIKTIVGVVEACHSLGVMHRDLKPENFLFDTPADDAVLKATDFGLS

VFYKPGQYFSDVVGSPFYVAPEVLLKHYGPEADIWSAAVILYILLSGVPPFWAETDSGIF

RQILHGKVDFESEPWPSISESAKDLLRKMLERDPQKRITAYEVLCHPWIVDDRVAPDKPL

DSAVLSRLKKFSAMNKLKKMALRVIAERLSEEEIGGLKELFKMIDTDNSGTITFQELKDG

LKKVGSELTETEIKDLMEAADIDNSGTIDYGEFLAATLHINKIEREENLVAAFSFFDKDG

SGYITIDELQQACKEFGLGDVHLDEMIKEIDQDNDGRIDYGEFAAMMRTGDGGMGRSRSL

RSSLTFSIADAFGMKDPTQDIK

>GhCPK17

MGSCISAPRKLVGIISKRCEYRYKAKGKAKAKAARFCQDGNTGKCINLRTRVLKESSGYN

ILGRYKMGKELGRGEFGITNECFDIRTGEAYACKKISKAKLRTEIDVEDVRREVEIMKHL

PKHPNIVAFREAFEDKEAVYLVMELCHGGELFDRIVAKGHYTERAAAKVVKTILEIIKVC

HDHGVIHRDLKPENFLLADGGETAPIKAIDFGLSTFYKPGQLFSDIVGSPYYMAPEVLRR

NYGKEVDIWSAGVILYIMLCGVPPFWADTEEGIAQAIIRGKIDFGRDPWPKVSTEAQDIV

KRMLDPNPQCRMAVHQVLEHPWIQNLENGRNVDLGENVCTRIKQFSLMNKFKKEVLRVVA

DNLPNEQIDSITEMFHMMDKDEDGQLSLEELKDGLQKLGHSVDHPEVQMLMQAADMDGNG

TLSCDEFIIMAVHLKRIGNDEHLREAFNVFDKNQSGYIEFEELEQALLHDNLHPHLIQNI

MVEIDKDKDGKISYAEFKTMMLTGMDWKMASRQYSRALLNAVSTKILRQSGQLK

>GhCPK18

MSRTSSGTKPTMVLPYQTPSLTEHYSIGKKLGQGQFGTTYLCTHKPTGQHFACKSIPKRK

LICQEDYDDVWREIQIMHHLSEHPHVVRIRGTYEDQLSVHLVMELCEGGELFDRIVQKGH

YSEREAAKLIKTIVGVVEACHSLGVMHRDLKPENFLFDTVEEDATLKATDFGLSVFYKPG

ESFSDVVGSPYYVAPEVLRKHYGPEADVWSAGVILYILLSGVPPFWAETEMGIFRQILQE

KIDFDSEPWPAISDSAKDLIRKMLDRHPKRRLTAHQVLCHPWIVDDTIAPDKPLDSAVLS

RLKQFSAMNKLKKMALRVIAERLSEEEIGGLKELFKMIDTDNSGTITYEELKDGLKRVGS

ELMESEIKDLMDAADIDNNGTIDYGEFLAATVHLNKLEREENLVGAFSFFDKDGSGYITI

DELQQACKEFGLSDVHLDEMIKEIDQDNDGQIDYGEFAAMMRKGNGGIGRRTMRRTINLG

DAFGVTANGAKELNSSV

>GhCPK19

MGLCQSLGFCLRRSHSHEIPISSSSESSPRPSHLFPKTTPQHFNPSSSKATSSSGIGTIL

LKPYVDVTTIYDLDKELGRGQFGITYLCTEKATGRKYACKSISRRKLRTDRDIEDVRRET

SIMQHLTGQPNIVEFKGAYEDRQNVHLVMELCSGGELFDRIIAKGSYSERQAASICRQVV

NVVNACHFMGVMHRDLKPENFLLVSKDEISPIKATDFGLSVFIEEGRMYKDLVGSAYYVA

PEVLNRKYGKEIDVWSAGVILYILLSGVPPFWGETEKEIFKAVLEGNLDLKSLPWPSITE

GAKDLIRKMLTRDPTKRITAAQALEHPWLKEGGDASDKPIDSAVLSRLKQFRVMNKLKKL

ALKVIAESLSTEEEIKGLQQMFKNIDTDGSGTITLGELRDGLARLGSKLTETEIKQLMDA

ADVDNSGTIDYIEFITATMHRHRLEREENIVKAFQFFDKDNSGFITRDELRQAMTQYGMG

DEATIDEVIEDVDTDKDGRINYEEFVAMMKRGTHDGDGNWQRHMNS

>GhCPK20

MAGEYNFDERTWKNISSSAKHLISNLLQVDPDRRPSAEQLLAHPWVIGDSAKQEQIDAEV

VSRLQSFNARRKLRAAAIASVLSSKVLLRTKRLRSLLGSHDLSKDEIDNLKSNFKKICAN

GDNATLPEFEEVLKAMNMSSLLPLATRIFDLFDSNRDGTVDMREIVCGFSSLKNSKGDHA

LRLCFEMYDTDRSGCITKEELASMLRALPDDCLPPDITEPGKLDEIFDRMDANSDGKVTF

EEFKDAMQRDSSLQDVVLFSLRQQ

>GhCPK21

MGCCSSKNRLTGCSSYKSGKHSIPVQDQKVVVVSQTQVPQAKQRHHNNHPQPPLANKTSQ

VKVQGTVLGKPLEDIRQYYTLGNELGRGQFGVIYLCTENSTGHTYACKSILKRKLTSLQD

KEDIKKEVQIMQHLSGQPNIVEFKGAYEDKDCVNIVMELCAGGELFDRIIAQGHYSERAA

AAICRQVVNVVQNFHFMGVMHRDLKPENFLLSSKDEDAMLKATDFGLSVFIEQGKQYRDI

VGSAYYIAPEVLRRSYGKEIDIWSAGVILYILLCGVPPFWAETEKGIFDAILEGELDFEN

DPWPSISESAKDLVRKMLTMDPNERLTAAQVLEHPWLREGGEASDKPIDSAVLSRLKQFR

AMNQLKKLALKVIAENLSEEEIQGLKAMFKNIDTDESGSITYEELKEGLARLGSKLTEAE

VKQLMEAADVDGNGTIDYIEFISATMHRYRLERDEDLYKAFQYFDKDNSGFITMDELEAA

MKDYRMGDEASIKQIISEVDTDNDGKINYDEFCAMMRGGAPQTAKLF

>GhCPK22

MGNCCTRGSPAAEDANDDKGDAPKQAEGSPPAGGAGSTSMSDAIGNVLGRPMEDIKATYT

IGKELGRGQFGVTHLCTHKTTGEQFACKTIAKRKLSTKEDIEDVKREVQIMHHLTGQPNI

VELKGAYEDKHSVHLVMELCAGGELFDRIIAKGHYSERAAASLLRTIVQIVHTCHSMGVI

HRDLKPENFLLLNNDEDSPLKATDFGLSAFYKPGEEFKDIVGSAYYIAPEVLKRKYGPEA

DIWSVGVMLYILLSGSPPFWAESENGIFNAILRGHIDFSSDPWPRISTQAKDVVRKMLNA

DPKQRLTASQVLSHPWIKEDGEAPDTPLDNAVLNRLKQFKAMNQFKKVALRVIAGCLSEE

EIMGLKEMFKGMDTDNSGTITLEELKQGLAKQGTKLSEYEVKQLMEAADADGNGTIDYDE

FITATMHMNRMDREDHLYHAFQHFDKDNSGYITTEELDQALREYGMHDDQDIKEIISEVD

IDNDGRINYDEFVAMMRKGNPEPNPKKRRELFI

>GhCPK23

MIFDNYFRRISKFNVILLGKKNKAIPYSADEYGVTHGSTAFKLQVLNELTGRDISAQYDL

GREMGRGEFGVTYLCTDPNSGEKFACKSISKKKLRTAVDIEDVRREVQIMKHLPKHPNVV

TLKDTFEDDDAVHIVMELCEGGELFDRIVARGHYTERAAAGVMKTIVEVVQMCHKHGVMH

RDLKPENFLFANKKEASPLKAIDFGLSVFFKPGELFNEIVGSPYYMAPEVLKRNYGPEVD

VWSAGVILYILVCGVPPFWAETEQGVAEAIIRSVIDFKRDPWPKVSDNAKDLVRKMLNPD

PKKRLTAQEVLEHPWLQHAKKVPNVPLGETVKARLKQFSVMNKLKKRALRVIAEHLSVEE

VADIKETFDMVDTKQRGKITLEELKAGLQKLGQQIPDADLQILVGAAGGDGDGTLNYGEF

VAVSVHLRKMANDEHLHKAFAFLDLNKSGYLEKEDLRDALNDEVDPCSEEVINAIMHDVD

TNKDGRISYEEFAAMMKAGTDWRKASRQYSRERFNSLSMKLMLVVAT

>GhCPK24

MGQCYGKVSQTQVNETTSNATTTVTEVIPADPGGQTPLQSSNGAVNYFQSVKNTPARSSS

QSPWPSPYPHGVTASPLPRGVSPSPARASRGSTPRRFFRRPFPPPSPAKHIKASLLKRLG

GKPKEGPIPEDPGTEPEQVLDKSFGYGKNFGAKYELGKEIGRGHFGHTCSARGKKGDLKD

QPLAVKIISKAKMTTAISIEDVRREVKILKALSGHKHLVKFYDACEDANNVYIVMELCEG

GELLDRILARGGRYTEEDAKAILVQILSVVSFCHLQGVVHRDLKPENFLFTSGGENADMK

LIDFGLSDFIRPDERLNDIVGSAYYVAPEVLYRSYSMEADIWSIGVITYILLCGSRPFWA

RTESGIFRSVLRSDPNFDDMPWPSVSPEAKDFVKRLLNKDHRKRMTSVQALAHPWLRDDS

RPIPLDILIYRLLKSYLHASPLKRAALKALSKALTEDELVYLRAQFRLLEPNRDGSVSLE

NFKMALAQNATEAMGESRVPDILNAMGLLAYRKMYFEEFCAAAISTHQLEAVEGWEQIAS

AAFEHFEQEGNRVISIEELARALTKVSQRRHDKNVTPSTRPVLVDLKLGHVKDRKRVPSN

RMEMARAMARNMGGSNQGPLQLMGK

>GhCPK25

MGHCCSKNVSVNNEATSTVNQSQPRPVPASATPSVETNSYAVSPFASPLPAGVAPSPSPA

RTPGRKFRWPLPPPSPAKPIMAAIMRRKGSNQAAPMEGTIPEDGEGAVLDKNFGYGKNLG

AKFELGKEVGRGHFGHTCWAKGKKGELKGKSVAVKIISKAKMTSAISVEDVRREVKILKA

LSGHKNMIKFHDAFEDANNVYIVMELCEGGELLDRILSRGGRYTEGDAKNIIVQILSVVA

FCHLQGVVHRDLKPENFLFTTRDEDAPMKIIDFGLSDFIRPDQRMNDIVGSAYYVAPEVL

HRSYSVEADMWSIGVITYILLCGSRPFWARTESGIFRSVLRADPNFDDSPWPSVSVEAKD

FVKRLLNKDHRKRMTAAQALAHPWLQDNNRVVPLDILIYKLVKSYIRATPFRRAAQKALS

KALPDDALRYLTAQFRLLEPKDGCVSLSNFKTALMKSRTDAMQESRVFDIINVMEPLYYK

KMDFEEFCAAAISTYQLEASEEWESIASTAFEYFEQDGNKVISVEELTLELNLGPSAHSL

LKDWIRVSDGKLSFLGYTKFLHSVTIRASNIRRR

>GhCPK26

MGNCCSRGNPESLTNELGVASPENNGNVSICSRNESFSKASPTQPPASPSEEASPKPTPK

PNPIGTVLCRPMEDVRNTYTIGKELGRGQFGVTYLCTHKVTGEQFACKTIAKRKLVNKED

IEDVRREVQIMHHLTGQPNVVELKGAYEDKHSVHLVMELCAGGELFDRIIAKGHYSERAA

ASLLRIIVQIVHTCHSMGVFHRDLKPENFLLLNQEENSPLKATDFGLSLFYKPGDVFKDI

VGSAYYIAPEVLKRKYGPEADIWSIGVMLYILLSGFPPFWAESENGIFSAILRGEIDFKS

DPWPAISPQAKDLITKMLNLDPKQRLTAQQVLNHPWIKEDGEAPDIPLDNAVLARLKQFK

AMNNFKKVALRVIAGCLSEEEIQGLKEMFKTIDSDNSGTITLEELKQGLAKQGTKLTEYE

VKQLMEAADADSNGTIDYEEFITATMHMNRMDREEHLYRAFQHFDKDNSGYITIEELEQA

LREYGMHDGKDIKEIISEVDNDNDGKINYDEFVAMMRRGNPEANPKKRRDDVMFDNSN

>GhCPK27

MGNCCSCGSSVEDANEKGDTITVEQAKSPSGSKQDSSPRNNTASPADSSKPSKSSPIGPV

LGRRMEDITATYNIGKELGRGQFGVTHLCTCKQTREQFACKTIAKRKLANKEDIEDVRRE

VQIMHHLTGQANIVELKGAYEDNHSVYLVMELCAGGELFDRIIAKGHYTERAAASLLRTI

VQVVHTCHSMGVIHRDLKPENFLLSNKDENSPLKATDFGLSVFYKPGEEFRDIVGSAYYI

APEVLKRKYGPEADIWSIGVMLYILLSGVPPFWAESENGIFNAVLRGHVDFSTAPWPSIS

AQAKDLVKKMLTVDPKQRLTAVQVLKHPWIKVDGEAPDTPLDNAVLSRLKQFKAMNQFKK

VALRVIAGCLSEEEIMGLKEMFKGMDTDNSGTITLEELRQGLAKQGTKLSEYEVKQLMEA

ADADGNGTIDYDEFITATMHMNRMDREEHLYHAFQHFDKDNSGFITIEELEQALREYGMH

DGRDLKEIISEVDIDNDGKINYDEFVAMMRKGNPKRRRDDIFL

>GhCPK28

MGNCCATPGSPVEKNKKGQKKNKANPFYGDEYAVSNGSATTFKLRVLKELTGQDISSQYD

LGRELGRGEFGVTYLCTDVNTGEKYACKSISKKKLRTAVDIEDVRREVEIMKHLPKHTNI

VTLKDTYEDDDAVHIVMELCEGGELFDRIVARGHYTERAAAVVMRTIVEVVQMCHKHGVM

HRDLKPENFLFGNKKENAPLKAIDFGLSVFFKPGERFNEIVGSPYYMAPEVLKRNYGPEV

DVWSAGVILYILLCGVPPFWAETEQGVAQAIIRSVIDFKRDPWPKVSDNAKDLVKKMLNP

DPKQRLTALEVLEHPWLQNAKKAPNVPLGETVKARLKQFSVMNKLKKRALRVIAEHLSVE

EVAGIKEAFDVMDTGKRGKINLEELRMGLQKLGQQIPDADLQILVEAADVDGDGTLNYGE

FVAVSVHLRKMANDEHLHKAFAFFDLNQSGFLEIEDLRDSLNDEVDTSEEVINAIMHDVD

TDKDGRISYEEFVAMMKAGTDWRKASRQYSRERFNSLSLKLMRDGSLQLGN

>GhCPK29

MGLCHGKPIENQQKQSRDISIPAEKDAAPNSNSSKSSNFPFYSPSPLPSLFKTSPAVPSV

NSTPLRFFKRPFPPPSPAKHIKSLLARRHGSIKPNEASIPEGNECEVGLNKSFGFSKHFT

SHYELGEEVGRGHFGYTCSAKAKKGSLKGNDAAVKVIPKSKMTTAIAIEDVRREVKILRA

LTGHKNLVQFYDAYEDDENVYIVMELCKGGELLDRILSRGGKYPEEDAKAVMVQILSVVA

FCHLQGVVHRDLKPENFLFTTKDEGSPLKAIDFGLSDYVKPDERLNDIVGSAYYVAPEVL

HRSYGTEADMWSIGVIAYILLCGSRPFWARTESGIFRAVLKADPSFDEAPWPSLSPDAID

FVKRLLNKDYRKRVTAAQALSHPWLANCHDIKIPSDMIIWRLVKAYIGSSTLRRAALGAL

AKTLTIPQLAYLREQFSLLGPKKSGFILMQNFKMVMLENSTDAMKDSRVLDYVNMISSIQ

YRKLDFEEFCASAISVHQLEGMETWEQHARRAYDLFDKDGNRPIMIEELASELGLSSSVP

VHVVLQDWIRHSDGKLSFLGFVRLLHGVSSRTFQKS

>GhCPK30

MGNCCVTSAAASHENNKKKNKKKGNKKQNPFDNSNGSPKLIVLEEPTGREIEQRYELGRE

LGRGEFGITYLCTDKDTGENFACKSISKKKLRTAVDIEDVRREIKIMKHLPEHPNIVTLK

DTFEDDNAVHLVMELCEGGELFDRIVARGHYTERAAAAVAKTIVEVVQMCHRHGVMHRDL

KPENFLFENKKETAALKAIDFGLSVFFKPGERFTEIVGSPYYMAPEVLKRNYGPEVDVWS

AGVILYILLCGVPPFWAETEQGVAQAILRSVVEFKRDPWPKVSASAKDLVKKMLNPDPKR

RLTAQEVLAHPWLQNAKTAPNVSLGETVKARLKQFSVMNKLKKRALRVIAEHLTVEETAD

IKERFQEMDTGNRGKINIDELRDGLHKLGQIISDADLQVLMDAGDVNRDGYLDYGEFTAI

SIHLRKMGNDNHLKKAFEFFDKDQSGYIEIEELRDELADELETNGEEVITAIMHDVDTDK

DGRISYDEFVAMMKAGTDWRKASRQYSRQRFNNLSLKLMKDGSLLSPDNPI

>GhCPK31

MGNNCFKTISNSICGPSEEEKEKEKEEVPNVTAESESKEKGASDVQNQPPEEIKIVKEGT

EREQEGKSKEETQMQQSKCELQILDSKPAQVSTQVTPQTAINNEEEHKVEPKKTPRRPHN

VKRQSCAGLKIDSVLQTKTGHLKEYYSLGTKVGNGQFGTTFVCVEKGTGNKFACKSIAKR

KLATLDDVEDVRREIQIMHHMSWHPNVVTIKGAYEDPMAVHVVMDLCAGGELFDRIVKRG

HYSERKAAELARVIVGFVEACHSMGVMHRDLKPENFLFVNDEEDSPLKAIDFGLSIFFKP

GDTFSVVVGSPYYVAPEVLNKCYGPEADVWSAGVIIYILLCGVPPFWGETEEEIFDEVLN

GEPDFTSDPWPSISESAKDLLAKMLVRNPKKRITAHEVLRHSWVQADGVAPDSPLDSLVL

GRMKQFSSMNKLKKMALRVIAQRLSQEEIAGLKEMFKMIDTDNSGQITFEELKAGLQSFG

ASLPESEFQALMQAADVNNSGSIDYQEFIAATLHLNMIQNEDNLMAAFSYFDRDGSGYIT

LEEIQKACQEFGIKDIHMDEMMREVDQDNDGRIDYNEFIAMMQKGNPEVGKRDREGKGLS

IGFREALPGS

>GhCPK32

MGCFSSKHKLPDPPSMPTTQPKQVQTQEVSMPQTRRPQQQVQTQKVSVPEAQVPQTRQPQ

AVSVPLKPSPASTRPIQTMEDTVLGKPLEDIKQYYKLGKELGRGQFGITYLCTENSTGNT

YACKSILKRKLRSKQDREDIKKEVQIMQHLSGQPCIVEFRGAYEDRQSVHLVMELCAGGE

LFDRIIAEGHYSERAAAGIFKSVVNVVHICHFMGVIHRDLKPENFLLSSKDAGAMLKATD

FGLSVFIEEGKRYRDIVGSAYYVAPEILRRSYGKEVDIWSAGIILYILLSGVPPFWAETE

KGIFDAISEGKLDFESLPWPSISESAKDLVRKMLTKDPKKRLTSAQVLEHPWMREDGEAS

DKPIGSAVLSRLKQFRAMNKLKKLALKVIAENLSEEEIKGLKVMFTNMDTDKSGTITYEE

LKTGLARLGSKLSEAEVKQLMEAADVDGNGTIDYIEFISATMNRYRLDRDELLYKAFQYF

DKDNSGYITKDELETAMKEYGMVDEASIRAVISEVDTDNDGRINYEEFCTMMRSGTQQTE

KAFLDTINSSIT

>GhCPK33

MGICLSTTKVFGASSNPSPDHHEEKQPASSTTTTNAKKESHKPTVKHQQQQQFKAKPSSR

KHGGNVPCGKRTDFGYRKDFEKRYTTGKLLGHGQFGYTYVAVDNENGDRVAVKKIEKMKM

VLPIAVEDVKREVKILEALKGHGNVVQFYNAFEDDSYVYIVMELCEGGELLDRILAKKDS

RYSEKDAAFVVRQMLKVAAECHLHGLVHRDMKPENFLFKSTKEDSPLKATDFGLSDFIRP

GKRFQDIVGSAYYVAPEVLKRKSGPESDVWNIGVITYILLCGKRPFWDKTEDGIFREVLK

NKPDFRRKPWPTISDSAKDFVKKLLVKDPRARLTAAQALSHPWVREGGDASEIPIDISVL

SNMRQFVKYSRLKQFALRALASTLNEEEIADLRDQFHAIDVDKNGVISLEEMRQALAKDL

PWKLKESRVIEILQAIDINTDGLVDFTEFIAAALHVNQMEEHDSEKWQMRSEAAFQKFDV

DRDGFITPDELRMHTGLKGSIDPLLEEADIDRDGKISLEEFRRLLRTASISARPIPSHRI

TWKL

>GhCPK34

MGSCISTQAKLINSISKRYYDRSGTKGKSKHARFDHSESRKSVSKCVSSTRKVLKNPSGK

NIFDLYEIGKKLGIGEFGVTHQCFDLETGEAFACKKIAKAKLRTEVDLEDVRREVEIMRH

LPKHPNIVTFREAFEDKEAIYLVMELCRGGELFDRILAKGHYSEQAAATIIKTILEIVKV

CHEHGVIHRDLKPENFLFADESESAPIKAIDFGLSIFYESGQRFSDIVGSPYYMAPEVLK

RNYGEEVDVWSIGVILYILLCGVPPFWAETEEGIAHAIIKGEIDFKRDPWPKVSGEAMEL

VKSMLRPNPYNRMTIQEILEHPWIQNPKHCPNVNLGENVRSRIKQFSLMSKFKKKVLRVV

ADNLSEDQTDSIIQMFNMMDTDENGHLSFEELRDGLAKIGHSIDDPDVQMLLESADVDGS

GTLSYEEFITMAVHLKRISDDQLSQAFQYFDKNQSGYIEVEELKEALLQDDPGPNNEKLI

KDIMLDVDEDKDDRISYQEFKAMMLSGMDWKMDSRQYSRVLLNAVSIKILRKSGQLK

>GhCPK35

MGNTCVGPSISKNGFFQSVSAAMWRSRMPDDSVSSSNGGIGSEVAAREPESPLPVLNKPP

EQVTIPKPETKQEAKEEAKPETKPETEPEKPAKPKFPHMKRVSSAGLRTESVLQTRTGNF

KEYFSLGRKLGQGQFGTTFLCLEKSTRREYACKSIAKRKLLTDEDVEDVRREIQIMHHLA

GHPNVISIKGAYEDAVAVHVVMELCAGGELFDRIIQRGHYTERKAAALTRIIVGVVEACH

SLGVMHRDLKPENFLFVNQQEDALLKAIDFGLSIFFKPGERFTDVVGSPYYVAPEVLRKH

YGPEADVWSAGVILYILLSGVPPFWAENEQGIFEEVLHGDLDFDSDPWPSISESAKDLVR

KMLIRDPRKRLTAHEVLCHPWIQIDGVAPDKPLDSAVLSRMKQFSAMNKLKKMALRVIAE

SLSEEEIAGLKEMFKMIDADNSGQITFEELKAGLKRVGANLKESEIYDLMQAADVDNSGT

IDYGEFVAATLHLNKIEKEDHLFAAFSYFDKDGSGYITPDELQKACEEFGIEDVRLEEMI

REVDQDNDGRIDYNEFVAMMQGGNVAGADPIDHVDTGSPKQEARQHENVQLMHIRTDAGE

>GhCPK36

MGNCNRPPIASNRFRPESDTGGTHHNGDNVKPVPGYSPPPKTHYYHSSTIASKNRLATPN

TPPIGRVFGRPMEDVRSTYIVGRELGRGQFGVTYLVTHKETKQHFACKSISRRKLLNRDD

IEDVRREVQIMYHLTGHRNIVELEKACEDRRSVNLIMELCAGGELFDRILAKGHYSEKEA

ANLCRQIVTVVHKCHSMGVMHRDLKPENFLFLSKDEDSPLKATDFGLSTFFKPGEVFKDF

VGTAFYMAPEVLHWRYGPEADIWSAGVILYVLLSGSPPFYGETEKSIFKSILQGNINFLS

DPWPSVSDSAKDLLSKMLRQDPKERLSASEVLNHLWMREDGDASDKPLDVAVLTRMKQFR

AMNKFKKVALKVIAENLSEEEIVGLKEMFKLMDTDNSQTITFDELKAGLCKLGTNVSESE

VRLLMEATDIDGNGTIDYVEFITATMHMNRMEKEKRLYTAFQHFDKDNSGYITMEELKQA

LQKYNTRDEKTINEILAEVDTDKDGRINYDEFVAMMRKSNPELVGNRLHT

>GhCPK37

MGQCYGKVNQSGVHEGTTTTMVVSADRDEATAQSANGAGNVPSVKNTPARSSSQSPWPSP

YPHGVSASPLPPGVSPSPARASRGSTPRRFFRRPFPPPSPAKHIKASLVKRLGGKPKEGP

IPEDRGTEPEQALDKNFGYGKNFGAKYELGKEVGRGHFGHTCSARGKKGELKDQPVAVKI

ISKAKMTTAISIEDVRREVKILKALSGHKHLVKFYDACEDANNVYIVMELCEGGELLDRI

LARGGRYTEDDAKAIVVQILSVVSFCHLQGVVHRDLKPENFLFTSGGEDADMKLIDFGLS

DFIRPDGRLNDIVGSAYYVAPEVLHRSYSLEADIWSIGVITYILLCGSRPFWARTESGIF

RSVLRSDPNFDDLPWPSVTPEAKDFVKRLLNKDYRKRMTAVQALTHPWLRDDSRPVPLDI

LVYKLVKSYLHATPFKRAALKALSKALTEDELVYLRAQFRLLEPSRDGSVSLENFKMALA

RNATEAMGESRVPDILNTMGTLAYRKIYFEEFCAAAISTHQLEAVEGWEQIASTAFEHFE

QEGNRVISIEELARELNVGPSAYSFLKDWIRVSDGKLSLLGYTKFLHGVTLRSSNTRHH

>GhCPK38

MGACLSTTKVIGSSSNAAAHHRKHQPSATTVTANEKKESRKPNNQQGQRQQVRSSQPLKV

KGKPSSTRQTGIIPCGKRTDFGYHKNFDQRYTIGKLLGHGQFGYTYVAIDKANGDRVAVK

KIDKNKMVLPMAVEDVKREVKILEALKGHENVVQFYNAFEDDSYVYIVMELCEGGELLDR

ILAKKDSRYSEKDAAVVVRQMLKVAAECHLHGLVHRDMKPENFLFKSTREDSLLKATDFG

LSDFIRPGKRFHDIVGSAYYVAPEVLKRRSGPESDVWSIGVITYILLCGKRPFWDKTEDG

IFKEVLKKKPDFRRKPWPTISNGAKDFVKKLLVKDPHARLTAAQALSHPWVREGGNASDI

PVDISVLNNLRQFVKYSRLKQFALRALASTLNEEEIADLRDQFDAIDVDKNGSISLEEMR

QALAKDLPWKMKEPRVLEILQAIDSNTDGLVDFKEFVAAALHVNQMEEHDSDKWQMLSQA

AFEKFDVDRDGFITPEELRMHTGLRGSIDPLLEEADIDKDGKISLSEFRRLLRTASISSR

NVPCSSDQRNSRKL

>GhCPK39

MGLCTSKPSPNPSFPPDSRNTPIHPPNKDIHHKSNSVPASPLPDVVNGNHNHNLDQAKGT

EEKESSNSNNEGKKSPFFPFYSPSPAHYLFSKKSPARSSANSTPKRFFRKPFPPPSPAKH

IRAVLARRHGSVKPNESAIPEGSEADGDGAAGATVAGLDKSFGFSKHFGSKYELGEEVGR

GHFGYTCAAKFKKGELKGQQVAVKVIPKAKMTTAIAIEDVRREVKILRALSGHNNLVQFY

DAYEDHDNVYIVMELCEGGELLDRILSRGGKYTEDDAKDVMIQILNVVSFCHLQGVVHRD

LKPENFLFTSKDENSQLKAIDFGLSDFVKPDERLNDIVGSAYYVAPEVLHRSYSTEADVW

SIGVIAYILLCGSRPFWARTESGIFRAVLKADPSFDEAPWPALSSEARDFVKRLLNKDPR

KRLTAAQALSHPWIKNYNDVKVPLDILIFKLMKAYLRSSSLRKAALRVLSKTLTVDELFY

LKEQFALLEPNKNGTISLENIKVALMRNATDAMKECRIPEFLASLSALQYRRMDFDEFCA

AALSVHQLEALDRWEQHARCAYELFEKDGNRAIVIDELASELGLSPSVPVHAVLHDWIRH

TDGKLSFLGFIKLLHGVSSRTFAKAQ

>GhCPK40

MGNCCRSPAAVAREDVKSNFSGRDHARKDSVSKQKPAITVLAGVPKDSIEEKYLVDRKLG

RGEFGVTYLCIDRGTRELLACKSISKRKLRTAVDIDDVRREVAIMKHLPKDSSIVSFKEA

CEDDNAVHLVMELCEGGELFDRIVARGHYTERAAAAVTRTILEVVQLCHKHGVIHRDLKP

ENFLFANKKENSPLKAIDFGLSIFFKPGERFSEIVGSPYYMAPEVLKRNYGPEIDIWSAG

VILYILLCGVPPFWAGKSSEQGVAQAILRGLIDFNHDPWPNISESAKSLVQQMLEPDPKL

RLTAKQVLEHPWLQNAKKAPNVPLGDVVKSRLKQFSIMNRFKRKALRVFAEFLSVEEVEG

IKEIFKRMNTDNDGIVSIEELKAVFGNVGSQLAESEVQMMLIEAVDANGKGTLDYGEFLA

VSLHVQRMANDEHLRRAFSYFDRDGNGFIEPDELRDALMEGEDDCTSVANDIFQEMDTDK

DGRISYDEFAAMMKTGTDWRKASRHYSRGRFNSLSIKLMKDGSLNLGRE

>GhCPK41

MGNCNACVRPDESPESKPNRSDQNQKGKKKARERKPNPFSEKIASPAPIRVLKDVIPLSH

RTRIGDKYILGRELGRGEFGITYLCTDRETREALACKSISKRKLRTAVDIEDVRREVAIM

STLPEHPNIVKLKATYEDNENVHLVMELCEGGELFDRIVARGHYTERAAANVAKTIAEVV

RMCHENGVMHRDLKPENFLFANKKEHAPLKVIDFGLSVFFKPGEKFSEIVGSPYYMAPEV

LKRNYGPEVDVWSAGVILYILLCGVPPFWAETEQGVALAILRGVIDFKREPWPQVSESAK

SLVRQMLEPDPRKRLTAQQVLEHPWLQNTKKAPNVPLGDIVRARLKQFSVMNRFKKKALR

VIAEHLSVEEVEVIKDMFALMDTDNDGKVSYEELRAGLRKVGSQLAEPEIKMLMEVADVD

GNGVLDYGEFVAVTIHLQKMENDEHFRRAFMFFDKDGSGYIELDELRDALADESGETDVD

ALNDIMREVDTDKDGQICYDEFVAMMKAGTDWRKASRQYSRERFKSLSLNLMKDGSLQLH

DAVTGQAVAV

>GhCPK42

MGNTCRGSGQLHQGFSEPEERSSKQSYASSDHSTSDYSRSGLNDQQLAPQVIPKPTTKDA

NLVTTVPPKKENSMKRGFDHHVCHVLGHKTPNIRDLYTIGRKLGQGQFGTTYLCTEISTS

FEYACKSICKRKLLSKEDVEDVRREIQIMHHLAGHKNIVTIKGAYEDTLYVHIVMELCSG

GELFDRIIERGHYTERKAAALTKIIVGVVEACHSLGVMHRDLKPENFLLVNKDDDFSLKA

IDFGLSVFFKPGQIFTDVVGSPYYVAPEVLLKHYGPEADVWTAGVILYILLSGVPPFWAE

TQQGIFDAVLKGDIDFYSDPWPVISDSAKDLIRKMLCSQPSERLTAHEVVCHPWISQNGV

APDRALDPAVLSRLKQFSAMNKLKKMALRVIAESLSEEEIAGLREMFTAMDTDNSGAITF

DELKAGLRRYGSTLKDTEIRDLMNAADVDNSGSIDYGEFIAATVHLNKLEREEHLVAAFK

YFDKDNSGYITVDELQQACAEHNITDVLLEDIIREVDQDNDGRIDYGEFVAMMQKGNSGI

GRRTMRNSLNLSMRDPVGAQ

>GhCPK43

MGLCTSKPSPNPSDSTNASINTRNNDIHRKPNSVSASPLPDGVNSKEDQGKQGEEEKESS

NSNNEGKKSPFFPFYSPSPAQYLFSKKSPARSSTNSTPKRFFRRPFPPPSPAKHIRAVLA

RRDGSVKPNEAAIPEGSEAEAAGATGTGLDKSFGFSKHFGSKYELGDEVGRGHFGYTCTA

KFKKGELKGQQVAVKVIPKAKMTTAIAIEDVRREVKILRALSGHSNLVQFYDAYEDHDNV

YIVMELCEGGELLDRILSRGGKYTEDDAKAVMIQILNVVAFCHLQGVVHRDLKPENFLFT

SKDENSQLKAIDFGLSDFVKPDERLNDIVGSAYYVAPEVLHRSYSTEADVWSIGVIAYIL

LCGSRPFWARTESGIFRAVLKADPSFDEAPWPSLSSEARDFVKRLLNKDPRKRLTAAQAL

SHPWIKKYNDVKVPLDILIFKLMKAYLRSSSLRKAALRALSKTLTVDELFYLKEQFALLE

PNKNGTISLENIKAVLMKNATDAMKDARIPEFLASLNALQYRRMDFDEFCAAALTVHQLE

ALDRWEQHARCAYEIFEKEGNRPIVIEELASELGLSPSVPVHAVLHDWIRHTDGKLSFLG

FVKLLHGVSSRTIAKAQ

>GhCPK44

MGNCNSQPSADSQFRPETNTGGGPNHGINVLPADASAPRPPKPSTHHNLSSSSPAPNQHT

APSTTPPIGRVLGRPMEDVRSTYVFGRELGRGQFGVTYLVTHKETNQQFACKSIATRKLI

NRDDIEDVRREVQIMYHLTGHRNIVELKGAYEDRHSVNLIMELCAGGELFDRIIAKGHYS

EREAANLCRQIVTVVHNCHSMGVMHRDLKPENFLLLSKDEDSPLKATDFGLSVFFKPGDI

FKDLVGSAYYVAPEVLRRRYGHEADIWSAGVILYILLSGVPPFYGETEQSIFDSILRGNI

DFASDPWPFVSSSAKDLVRKMLRHDPKDRLSAVEVLNHPWIREDGDASDKPLDVAVLTRM

KQFRAMNKLKKVALKVIAENLSEEEIIGLKEMFKSMDTDNSGTITFEELKAGLPKLGTKL

SENEVRQLMEAADVDGNGAIDYIEFITATMHMNRMEREDHLYTAFQYFDKDNSGYITMEE

LEQALKKYNMGDEKTIKEIIAEVDTDRDGRINYDEFVAMMRKGNPELANNRRRK

>GhCPK45

MGNTCVGPSISKNGFFQSVSAVMWPNRSPEGSVSHRKTGNEVASKEPESALPVQSRPPEQ

MTMPKSETRQETKSKKPKKPKPPHVKRVSSAGLRTESVLQTKTGNFKEYYSLGKKLGQGQ

FGMTFLCVEKSTGKEYACKSIAKRKLLTDEDVEDVRREIQIMHHLAGHPNVISIKGAYED

AMAVHVVMELCAGGELFDRIIQRGHYTERKAAALTRTIVGVVESCHSLGVMHRDLKPENF

LFVNQQEDSLLKTIDFGLSMFFKPGETFTDVVGSPYYVAPEVLQKHYGPEADVWSAGVIL

YILLSGVPPFWAESEQGIFDQVLHGDLDFESDPWPNISESAKDLVRRMLIRDPKKRLTAH

AVLCHPWIQVDGIAPNKPLDFAVLSRLKQFSAMNKLKKMVLRVIAENLSEEEIAGLKEMF

KMIDTDNSGQITFEELKAGLKRVGANLKESEIYDLMQAADVDNSGTIDYGEFVAATLHLN

KIEREDHLFAAFSYFDKDGSGYITPDELQQACEEFGIEDVRLEEMIHEVDQDNDGRIDYN

EFVAMMQKGHVTTNAGSAGAGKEGLQHSFGIGFREAFKL

>GhCPK46

MGYNTTTGTNLAPSISPRANTMRRAFDHQAHFVLGRKTPNIHELYTFGRKLGQGQFGITY

LCTDISTGIEYACKSISKRKLIRKEDVEDVRREIQIMHHLAGHKNIVSIKGAYEDTLYVY

IVMEFCSGGELFDRIIQRGHYSERKAAELTKIIVGVIEVCHSLGVMHRDLKPENFLLVNK

DDDFSLKAIDFGLSAFFKPGEIFSDVVGSPYYVAPEVLLKHYGPEADIWSAGVILYILLC

GLPPFWAETIQGIFAAVLKGHIDFSSYPWPLISDSAKDLIRKMLCIHPSERLTAHEVLCH

PWICKNGVAPDKALDPAILSRLKQFSAMNKLKKMALRVIAESLSEEEIAGLREIFTAMDT

GKSGAITFDELKAGLQRYGATLKDTEIQDLMNAADVSNSGTIDYGEFIAATIHLNKLERK

EHLVAAFQYFDKDKSGYITIGELKQACAELNMKNVLLEDIIQEVDQDNDGRIDYAEFVAM

MQKGNAGVGRRPMRNSLNMSIRDVPGSQ

>GhCPK47

MRRAIDHQAYYVLGHKTPNILELYTLGRKLGQGQFGVTYLCTEISTGIEYACKSISKRKL

ICKEDVEDVRREIQIMHHLAGHKNIVTIKGAYEDPLFVHIVMELCSGGELFDRIIQRGHY

TERKAAELTKIIVGVVEACHSLGVMHRDLKPENFLLVNKDDDFSLKAIDFGLSVFFKPGE

VFTDVVGSPYYVAPEVLLKCYGPEADVWTAGVILYILLTGVPPFWAETQQGIFDAVLKGY

VDFDSDPWPLISDSAKDLIQKMLCSQPSERLTAHEVLCHPWICENGVAPDKALDPAVLSR

LKQFSAMNKLKKMALRVIAESLSEEEIAGLREMFTAMDTDNSGAITFDELKAGLRSYGST

LKDTEIRDLMDAADVDNSGTIDYGEFIAATVHLNKLEREEHLVAAFRYFDKDNSGYITVD

ELQQACTEHNMTDVLLEDIIREVDQDNDGRIDYGEFVAMMQKGNAGVGRRTMRNSLNMSM

RDVPAAL

>GhCPK48

MGGCLTKNKDPMPQHNGYRSGATTTAAVHQQRYQDPVRPAPIQPQVYHIPQKPGTQTPWK

PAVPAPSPKPAPNIDTILGKPFEDIRMHYTIGKELGKGQFGVTYHCTENSTGKQYACKTI

SKRKLVRKNDKEDMKREIQIMQHLSGQPNIVEFKGAYEDKQSVHLVMELCAGGELFDRII

AKGHYSERAAASICRAIVNVVHACHFMGVMHRDLKPENFLLSSKGENALLKATDFGLSVF

IEEGKVYQDIVGSAFYVAPEVLLRKYGKEIDIWSAGVILYILLSGVPPFWAETEKGIFDA

ILEGEIDFESQPWPSISDSAKDLVCRMLTQDPKKRITSTQVLEHPWIREGGNASDKPLDS

AVLSRMKQFRRMNKLKQLALKVIAENLSTEEIQGLKQMFANIDTDNSGTITYDELKTGLA

RLGSKLTETEVQQLMEAADVDGNGTIDYIEFITATMHRHRLERDEDLYKAFQHFDKDNSG

HITRDELEAAMKEYGMGDDDTIKEIISEVDTDNDGKINYEEFRDMMRILVLARALN

>GhCPK49

MGNCCTRGDGSDKLEKAAAGYGNSDADPTVTSQQTSYRTAPSSQGASTVGKQSKPAPMGP

VLGRPMEDVKATYTIGKELGCGQFGITHLCTHKTTGEQFACKTIAKRKLSSKEDVEDVRR

EVQIMHHLTGQPNIVELKGAYEDKHAVHLVMELCGGGELFDRIIAKGHYTERAAASLLRT

IMQIVHTFHSMGVIHRDLKPENFLLLGKEENSPLKVTDFGLSVFFKPDEIFKDIVGSAYY

IAPEVLKRKYGPEADIWSVGVMLYILLSGVPPFWAESENGIFNAIIKSHVDFSGKPWPSI

SHQAKDLVKRMLNPDPKRRLTAAQVLSHPWIKEDGEAPDTPLDNAVLSRLKQFKAMNQFK

KVALKVIAGCLSEEEIRGLKEMFKAMDTDNSGTITLEELRQGLAKQGTKLSEYEVKQLME

AADADGNGTIDYDEFITATMHMNRMDREEHLYHAFQHFDKDNSGYITTEELEQALREYGI

NDSTDIKEILSEVDADNDGRINYDEFVAMMKKGNPEPNPKKRRDVVV

>GhCPK50

MGNSCAKSAATEEDENEDNKQDGNEGEAKDPGQSESKEPEESGSVRKQPPEEMKIVREED

KTGKEQESKSTEKAAEMTQRQQSKSHPQRLNSKPSQLAGFNKEGSKTGNKTRKAHNVKRQ

SCAGLQVGSVLQTKTGHLKEYYNLGRKLGQGQFGTTFLCIEKGTGKEYACKSIAKRKLTT

TEDVDDVKREIQIMHHLAGHPNVISIKGSYEDNMAVHVVMELCAGGELFDRIVKRGHYSE

RKAAEIARIIVAVVEACHSMGVMHRDLKPENFLFVNNEEDSPLKAIDFGLSIFFKPGDIL

NDVVGSPYYVAPEVLRKHYGPEADVWSAGVITYILLSGVPPFWGETEQEIFNEVLNGELD

FSSDPWPNISESAKDLVTKMLDRDTKRRIKAHEVLRHPWVQVDGVAPDKPLDSVVLSRMK

QFSAMDKLKKMALRVIAQRLSEEEIAGLKEMFKMIDADNSGQITYDELKEGLKRFGANLA

ESEFRALMQAADINNSGTIDYEEFVTATLHLNKIEREDNLLAAFSYFDRDSSGYITLDEL

QKACQEFGIQDIHLDEIMREVDQDNDGRIDYNEFVAMMQEGNPKLGKKGKE

>GhCPK51

MGSCLTKSKDSKPKHNGYGSGPTTTAAVHQQRYQEPVRPAPVQSQFHHIPEKPGTQTSWK

PVAPSPSPKPVAPRVDTILGKPFEDIRVHYTIGKELGKGQFGVTYLCIENSTGKQYACKT

ISKKKLVTRNDKEDMRREIQIMQHLSGQPNIVEFKGAYEDKLSVHLVMELCAGGELFDRI

IAKGHYSERAAASICRAIVNVVHACHFMGVMHRDLKPENFLLSSKGENALLKATDFGLSV

FFEDGKVYKDIVGSAYYVAPEVLLRKYGKEIDIWSAGVILYILLSGVPPFWAETEKGIFD

AILEGEIDFESQPWPSISESAKDLVRRMLTQDPKKRITSTQVLEHPWIREGGSASDKPID

SAVLSRMKQFRRMNKLKQLALKVIAENLSSEEIQGLKQMFANIDTDNSGTITYEELKTGL

ARLGSKLTEAEVQQLMEAADVDGNGSIDYIEFITATMHRHRLERDEHLYKAFQHFDKDNS

GYITRDELEAAMKEYGMGDDDTIKEIISEVDTDNDGKINYEEFRDMMRSGTQHTQLF

>GhCPK52

MHAFYILRKPTCQDFLKIDAGITVGEILQKGDTDMADSPMLKPRKPLKNSPNQSRVRSTY

ESLLIVAKGHYTERAAAKVIKTILEIVKVCHEHGVIHRDLKPENFLLADESETAPIKVID

FGLSIFYEPGERFSDIVGSPYYMAPEVLRRNYGKEIDIWSTGVILYILLCGVPPFWDDTE

EGIARAIIRGVIDFERDPWPKVSAEVKDLVRSMLDPNPYTRISLQEVLEHPWIQNLQNAP

NFNLGENVGARIKQFSLMSKFKKKVLRVVADNLPNEQIDVIIEMFNMMDTDENGYLSFEE

LRDGLQKIGHSVGDPDVRMLMEAADIDGNGTLSCEEFVIMVVHLKRIGNDEHLAQAFNHF

DKNQSGYIEFEELKETLMQDDPGPNNEQLIKDIMQDVDKDKDGRISYQEFKAMMLTGMDW

KMASRQYSRALINAVSIKILRQSGQLK

>GhCPK53

MNNQSSSIPTTTATKGWVLPYETPRLRDHYVVGKKLGQGQFGTTYECIHKATGTVFACKS

IPKRKLLCREDYDDVWREIKIMHHLSEHPSVVRIEGTYEDSVFVHLVMEICLGGELFDRI

VAKGHYSEREAAKLIKTIVGVVEACHSLGVMHRDLKPENFLFDSPGDDAVLKATDFGLSI

FYKPGQRYADVVGSPFYVAPEVLCKHYGPEIDIWSAGVILYILLSGVPPFWAETESGIFR

QILHGKLDFTSEPWPSISESAKDLIRKMLERHPKSRISAYQVLCHPWIVDDRVAPDKPLD

SAVLSRLKQFSAMNKLKKMALRVIAERLSEEEIGGLKELFKMIDTDSSGTITYQELKDGL

KKVGSELMESEIKSLMEAADIDNNGTIDYGEFIAATLHMNKLEREENIVAAFTFFDKDGS

GYITVDELQQACKEFGLGDVHLEDMIKEIDQDNDGRIDYGEFAAMMRKGDGLGRSISMRS

NLNKNIADAFGLGVKDLTSISSSSNSNS

>GhCPK54

MGNTCRGSLKGKLHKGDNQPKDHCSSRNNTSSGRSTTTTDYSPSTLNSQQLIAQEFSKET

NQKETHLPVINPTKKDNNNNTMRRGIDHQAYYVLGHKTTNIRDLYTLGRKLGQGQFGTTY

LCTEISTGTEYACKSISKRKLISNEDVEDVRREIQIMHHLAGHKNIVTIKGAYEDTLYVH

IVMELCSGGELFDRIIQRGHYSERKAAELTKIIVGVVEACHSLGVMHRDLKPENFLLVNK

DDDFSLKAIDFGLSVFFKPGQVFTDVVGSPYYVAPEVLLKHYGPEADVWTAGVILYILLS

GVPPFWAETQQGIFDAVLKGHIDFDSDPWPLISDSAKDLIRKMLCSRPSERLTAHEVLCH

PWICENGVAPDRALDPAVLSRLKQFSAMNKLKKMALRVIAESLSEEEIAGLREMFTSMDT

DNSGAITFDELKAGLRRYGSTLKDTEIRDLMDAADVDNSGTIDYGEFIAATVHLNKLERE

EHLVAAFRYFDKDGSGYITVDELQQACAEHNMTDVLLEDIIREVDQDNDGRIDYGEFVAM

MQKGNAGIGRRTMRNSVNISMRDAPGAM

>GhCPK55

MGACLSATKVSGGSSGNTTAHHRKTATNAEKESQKPNNQQVRCSQPLKNKQKPKKQSGII

PWGKRTDFGYDKDFDQRYTIGKLLGHGQFGYTYVAIDKVNGDRVAVKKIDKNKMILPIAV

EDVKREVKILKALKGHENVVQFYNAFEDDSYVYIVMELCEGGELLDRILAKKDSRYSEKD

AAVVVRQMLKVAAECHLHGLVHRDMKPENFLFKSTRPDSPLKATDFGLSDFIKPGKRFRD

IVGSAYYVAPEVLKRRSGPESDVWSIGVITYILLCGKRPFWDKTEDGIFKEVLKNKPDFR

RKPWPTISNDAKDFLKKMLVKDPRARLTAAQALSHQWVREGGNASDIPVDISVLSNLRQF

VKYSRLKQFALRALASTLNEEEIADLRDQFDAIDVDKNGSISLEEMRQALAKDLPWKLKD

SRVLEILQAIDSNTDGLVDFTEFVAAALHVNQMEEHDCDKWQMRLQAAFEKFDVDRDGYI

TPEELRMHTGLRGSIDPLLEEADIDKDGKISLSEFRRLLRTASMGSRNVSSPSGNRNTQK

L

>GhCPK56

MGNCCATPSTTASHEKKEKKGKKKQNPFSLDYGQHHHGNGGHKLTVLNDPTGGEIEQRYE

LGRELGRGEFGITFLCTDKETGDTFACKSISKKKLRTAVDIEDVRREVEIMKHLPHHPNI

VTLKDTYEDDNAVHLVMELCEGGELFDRIVARGHYTERAAAAVTKTIVEVVQVCHKHGVM

HRDLKPENFLFANKKETAALKSIDFGLSVFFKPGEIFTEIVGSPYYMAPEVLKRNYGREV

DVWSAGVILYILLCGVPPFWAETEQGVAQAIIRSVIDFKRDPWPKVSENAKDLVRKMLNP

DPKRRLTAQEVLDHPWLQNAKKAPNVSLGETVKARLKQFSVMNKLKKRALKVIAEHLSVE

EVAGIKEGFQLMDTANRGKINIDELRVGLHKLGHTIPDADLQILMEAGDVDKDGYLDYGE

FVAISVHLRKMGNDEHLKKAFEFFDRNQSGYIEIEELRDALTDEVETNSEEVISAIMHDV

DTDKDGRISYDEFAVMMKAGTDWRKASRQYSRERFNNLSLKLMKDGSLQMNNEPRRPNTI

DKVLLMKNGC

>GhCPK57

MGNCNGLPSTGNQFQPLSDSGVGLLDGGINIQPAPPPPRPQQSSTHHHHPSPAVGRVLGR

PMEDVRSTYVFSGELGRGQFGITYLVTHKKTKQQFACKSIPKRKLINREISKMSAEKNIV

ELKGAYEDHQSVNLIMELCAGGELFNRIIAKGHFSERKAANLCRQIVMMLHNCHSMGVMH

RDLKPENFLFLNKDEDSPLKPTDFGLSVFFKPGDVFKDLVGSAYYVAPEVLRRRYGPEAD

IWSAGVTLYVLLSGVPPFYGETEQSIFDSILRGNIDFSSDPWPSISSSAKDLVRKMLRDD

PKERLSASEVLNHQWMREDGDASDKPLDIAVLTRMKQFSAMNKLKKVALKVIAENLSEEE

IIGLKEMFKSMDTDNSGTICFEELKTGLPKLGTKLSESEVRQLMEAADFDGNGAIDYIEF

ITATMHMNRTEREDHLYTAFQYFDENNSGFITMEELEQALRKYNMGDEKTIKEIIAEVDT

DRDGRINYDEFVAMMRKGNPELVGSRRRKHAKFSLLPNHQTADEELSTYHNPRYSYRNPQ

SLTVKRFAERHKKAKTALLLLVLFGTCLLICVGFLTPAISIRSAIEGVKVRSSELHYGVV

LIIACILLVGLFVLQHRGTYRVAFMFAPIVILWSLSIAAIGVYNILKWNPRVYKALSPYY

IYTFFRDTRYDGWISIGGVLLCVTGAEAMFADLGQYTAASIRLSFFCIIYPCLVLQYMGQ

AAFLSKNFAAVSTSFYASIPDSLFWPVLVMATLAAIVASQSVISATFSIVKQSYAIGCFP

RVKVVHKSKWFRGQIYIPEINWVLLVLCLAVTVGFRDINHLGNAYGLTYMSAMFVTTWLT

ALIINFVWGQSLVLALLFALFFGSIEIIFLSSSFIRISKGGWVPLVLSVIFMLIMFVWHY

GSRKKYLYDFHNKVPMKQILTLGPSLGIVRVPGIGLIFTALVSGVPATFTRFLTNLPTFY

QVAVFVCEKTVLVPYVPQKERYLIGRIGPKSFRMYRCIVRNGYKDVQKNEDDFENDLIMS

IAEFIQLEAEGSGTLEGSVDGRMAVVRTSEKFGKRLEISELERNGEASSSMPPTILNSSK

SHILQYLQSTYEMESPGSTLRRRVRFKLQPDMNYRDPNVKDELLELVEAKQSGVAYVLGH

SHIKAKMNAPCLKRFMIHVAYLFLRKNCRAPAVVLNIPQTCLIEVGMNYYL

>GhCPK58

MEGLGGMCKEKWKGESFSDVVGSPYYVAPEVLRKHYGPEADVWSAGVILYILLSGVPPFW

AETEMGIFRQILQGKIDFDSEPWPAISDSAKDLIRKMLDRHPKRRLTAHQVLCHPWIVDD

TIAPDKPLDSAVLSRLKQFSAMNKLKKMALRVIAERLSEEEIGGLKELFKMIDTDNSGTI

TYEELKDGLKRVGSELMESEIKDLMDAADIDNNGTIDYGEFLAATVHLNKLEREENLVGA

FSFFDKDGSGYITIDELQQACKEFGLSDVHLDEMIKEIDQDNDGQIDYGEFAAMMRKGNG

GIGRRTMRRTINLGDAFGVKSNGAKELNSSV

>GhCPK59

MGNVCATLGICSVCASCCGPKPAEEDQKKTDNEGEKSETKEAEAGAAAADGAEVQKDPPE

EMKIVKEEKGPGEETLSQRQKSKVTQRQQSSMTRQQSMSMSTPMQRQQSTLQRQQSKVTQ

RQQSKAPPQRLESKPSQVAVAAATAAVAGKEEAKADAAKPRKPHNVKRQSCAGLKVDAVL

QTKTGNLKEYYNLGKKLGQGQFGTTFLCVEKGTGKEYACKSIAKRKLVTPEDVDDVKREI

QIMHHLAGHPNVVTIKEAYEDSVAVHVVMELCAGGELFDRIVQRGHYSERKAAELARTIV

GVVEACHSMGVMHRDLKPENFLFVNEQEDSPLQAIDFGLSIFFKPGEVLSDVVGSPYYVA

PEVLQKHYGPEADVWSAGVIVYILLSGVPPFWGETEQEIFEEVLHGELDFTSDPWPNISE

SAKDLVKKMLVRDAKKRITAHEVLRHPWVQVDGVAPDKPLDSVVLSRLKQFSAMNKLKKM

ALKVIAQRLSEEEIAGLKEMFKMIDTDNSGQITYDELKDGLKRFGADLDESEFRALMQAA

DVNNNGTIDYEEFVAATLHLNKIEREDNLMAAFSYFDKDGSGYITQDELQKACQEFGIEE

IHLDEMIGEVDQDNDGRIDYNEFVAMMQKGNPDLGKKTLGIREALPPS

>GhCPK60

MGNACAGPSNLGGNGFFQSVTQAVWRQRPPNQDQLPAANEDNSNKGSDESNKSKGSDDCQ

DSAATENIAPEPVKIPNAEPMNKEGNNAKKASGQKPNNLKRLTSTELESVLGRKTGNMKE

LYSLGRKLGQGQFGTTFFCVEKSTGKEFACKSIAKRKLTSTDDLEDVRREVHIMHHLAGH

PNVIQIIGAYEDAVAIYLVMEFCAGGELFDRIIQRGHYTERRAAALARVMVGVVQACHSL

GVMHRDLKPENFLFINKDEDAPLKAIDFGLSMFFKPGEIFNDVVGSPYYVAPEVLQKHYG

PECDVWSAGVIIYILLCGVPPFWDETEQGIFEQVMRGELDFTSEPWPSISNSAKDLVRRM

LIKDPKKRLTAHEVLCHPWVQEDGIAPDKPLDSAVLSRLKQFSAMNKLKKIAIRVIADSL

SEEEIAGLKEMFKMIDADNSGNITLEELKIGLEKVGSKLKDSEINGLMQAADIDNSGTID

YSEFIAAMLHLNKIQKEDHLFAAFNYFDKDGSGYITPDELQKACEQFGLQDVHLEDVIRE

VDQDNDGRIDYSEFVAMMQDTGLTGKHKTFKH

>GhCPK61

MGNCCRSPAAVAREDVKSNFSGHDHGRKDSVSKQKPPITVLNGVPKENIEEKYLVDRELG

RGEFGVTYLCIDRGTRELLACKSISKRKLRIAVDIEDVRREVAIMKHLPKNSSIVSLKEA

CEDDNAVHLVMELCEGGELFDRIVARGHYTERAAAAVTRTIVEVVQLCHKHGVIHRDLKP

ENFLFANKKENSPLKAIDFGLSIFFKPGERFSEIVGSPYYMAPEVLKRNYGPEIDIWSAG

VILYILLCGVPPFWAESEQGVAQAILRGLIDFKRDPWPNISESAKNLVRQMLEPDPKLRL

TAKQVLEHPWLQNAKKAPNVPLGDVVKSRLKQFSMMNRFKRKALRVIAEFLSVEEVEDIK

VMFNKMDTDNDGIVSVEELKAGFKNYGSQLAEPEVQMLIEAVDANGKGTLDYGEFLAVSL

HLQRMANDEHLRKAFSYFDKDGNGFIEPDELRDALMEDGADDCTNVANDIFQEVDTDKDG

RISYDEFAAMMKTGTDWRKASRHYSRGRFNSLSIKLMKDGSLNLGNE

>GhCPK62

MGNTCRGPSKGNLYKGYNTSSHRSSSNNNNNNPPSKPLIPRESGEETGENDNHSASISAS

EQESIMKLGNDNQTYFVMGHKTPNIRDLYTLGPKLGQGQFGTTYLCTELSTGIEYACKSI

SKRKLVAKEDVDDVRREIQIMYHLAGHNNIVTIKGTYEDSLYVHIVMELCSGGELFDRII

QRGHYTERKAAELTRIIVGVVEACHSLGVMHRDLKPENFLLVNKDDDFSLKAIDFGLSVF

FKPGQVFTDVVGSPYYVAPEVLQKHYGPEADVWTAGVILYILLSGIPPFWGETQQGIFDA

VLKGFIDFDSEPWPLISDSAKDLIRKMLCSQPSERLTAHKVLCHPWICENGVAPDKALDP

AILSRLKQFSAMNKLKKLALRVIAESLSEEEIAGLREMFKSMDTDNSGAITFDELKAGLR

RYGSTLKDAEIRALMDAADIDNSGTIDYGEFIAATVHLNKLEHEEHLVAAFQYFDKDRSG

YITVDELQQAWAEYNLTDVFFEDIIREVDQDNDGRIDYGEFVDMMKKGNGWIGRQTMRNS

LNISMRNSHDSQ

>GhCPK63

MGNLCSRSDPAANPDEKGEPGPENELNTSTSMNEDSPNSPPKASPTQSISSKPSNKPNPI

GPVLGRPMEDIKTTYNIGKELGRGQFGVTHLCTNKSTGEQFACKTIAKRKLANKEDIEDV

RREVQIMHHLTGQSNIVELKGAFEDKHSVHLVMELCAGGELFDRIIAKGHYTERAAASLL

RTVVQIVHTCHSMGVIHRDLKPENFLLLNKDEDSPLKATDFGLSVFYKPGEEFKEIVGSA

YYIAPEVLKRKYGPEADIWSIGVMLYIFLSGVPPFWAESENGIFNSILRGHIDFSSDPWP

SISPQAKELVKKMLNSDPKQRLTAVQVLSHPWIKEDGEAPDTPLDNAVICRLKQFKAMNN

FKKVALRVIAGCLSEEEIMGLKEMFKGMDTDNSGTITLEELKQGLAKQGTKLTEYEVQQL

MEAADADGNGTIDYDEFITATVHMNRMDREDHLYHAFQHFDKDNSGYITTEELEQALREH

GMHDANIKEIVSEVDSDNDGRINYDEFVAMMRKGNPEAHTKKRRELSVNIET

>GhCPK64

MGICQSLCRCFSKSHEIPVSSSSDSPPRPYQPLTVSTSGGQNPSFPKAPSSSQAGTILLK

PYVDITSLYDLRKELGRGQFGITYLCIEKATKREYACKSISRRKLTTDKDVDDVRREISI

LQHLTGQPNIVEFKGAYEDAWNLHLVMELCSGGELFDRITAKRSYSERQASSICRQIMNV

VHACHFMGVMHRDLKPENFLMVSKDEDSQIKATDFGLSVFIEEGRMYKDLVGSPYYVAPE

VLQRKYGKEIDVWSAGVILYILLSGVPPFWGETEKEIFKAVSEGNLDLKSQPWPTISEGA

KDLIRKMLARDPKKRITAAQALEHPWMKEGGEASDKPIDSAVLSRLKQFRVMNKLKKLAL

KVIAENLSSEEEKKGLQQMFNNIDTDGSGTITLEELRDGLARLGSKLTEPEIKQLMDAAD

VDKSGTIDYIEFVTATMHRHRLDREDNIRKAFNFFDKDSNGFITRDELRQAMTQYGMGDE

ATIDEVIEDVDTDKDGRINYEEFVAMMKRGTQDGDGMATSISTSATKHEEPIRTSYAGVQ

LEETVDETKQGKLRLDSWISSRIQGISRARVQSSIKSGLVKVNGRVVDKVSHSLRAGDKV

DCVISDLQPLKAEPEDIPLDIVFEDDHVLVVNKPSHMVVHPAPGNANGTLVNGILHHCSL

PTVASSEKEVLFDTQDMSDDEQDIFHGASAGAASVRPGIVHRLDKGTSGLLVVAKDEYSH

AHLSEQFKQHTIQRVYISLTCGVPSASSGRVDIPIGRDSNNRIRMVAVPGLSHHGQARHA

ASRYKVIEVLAGGGSALVQWRLETGRTHQIRAHAKYMGIPLLGDEVYGGTKNMALSLLRP

RTPPYYNDELSRLVSRLERPYLHALVLGFEHPHSGEKMRFSCPPPPDFGEILNHLRKIGI

EKPISKE

>GhCPK65

MNKKIAGSSSRPRKPTGTVLPYQTQRIRDHYFLGKKLGQGQFGTTYLCTDKVTGIRYACK

SIPKRKLVCREDYDDVWREIQIMHHLSENPFVVQIKGTYEDAVFVHLVMELCAGGELFDR

IVAKGHYSEREAAKLIKTIVGVVEACHSLGVMHRDLKPENFLFDTPADDAVLKATDFGLS

VFYKPGQYFSDVVGSPFYVAPEVLLKHYGPEADIWSAAVILYILLSGVPPFWAETDSGIF

RQILHGKVDFESEPWPSISESAKDLLRKMLERDPQKRITAYEVLCHPWIVDDRVAPDKPL

DSAVLSRLKKFSAMNKLKKMALRVIAERLSEEEIGGLKELFKMIDTDNSGTITFQELKDG

LKKVGSELTETEIKALMEAADIDNSGTIDYGEFLAATLHINKIEREENLVAAFSFFDKDG

SGYITIDELQQACKEFGLGDVHLDEMIKEIDQDNDGRIDYGEFAAMMRTGDGGMGRSRSL

RSSLTFSIADAFGMKDPTQDIK

>GhCPK66

MGSCISAPRKLVGIISKRCEYRYKAKGKAKAKAARFCQDGNTGKCINLRTRVLKESSGYN

ILGRYKMGKELGRGEFGITNECFDIRTGEAYACKKISKAKLRTEIDVEDVRREVEIMKHL

PKHPNIVAFREAFEDKEAVYLVMELCHGGELFDRIVAKGHYTERAAAKVVKTILEIIKVC

HDHGVIHRDLKPENFLLADGGETAPIKAIDFGLSTFYKPGQLFSDIVGSPYYMAPEVLRR

NYGKEVDIWSAGVILYIMLCGVPPFWADTEEGIAQAIIRGKIDFGRDPWPKVSTEAKDIV

KRMLDPNPQSRMAVHQVLEHPWIQNLENGRNVDLGENVCTRIKQFSLMNKFKKEVLRVVA

DNLPNEQIDSITEMFHMMDNDEDGQLSLEELKDGLQKLGHSVYHPEVQMLMQAADMDGNG

TLSCDEFIIMAVHLKRIGNDEHLREAFNVFDKNQSGYIEFEELEQALLHDNLHPHLIQNI

MVEIDKDKDGKISYAEFKTMMLTGMDWKMASRQYSRALLNAVSTKILRQSGQLK

>GhCPK67

MGGCLTKTKGSNPQHNNGYKSGATTTTAAEQPQATHIPEKPGTQAPWKPVVPTPSAKPAP

KSDTILGKPYEDIRLHYTIGKELGKGQFGVTYLCIENSTGKQYACKTISKRKLITKNDKE

DMRREIQIMQHLSGQPNIVEFKGAYEDKLSVHLVMELCAGGELFDRIIAKGHYSERAAAS

MGRAIVNVVHACHFMGVMHRDLKPENFLLSSKGENALLKATDFGLSVFIEEGKVYRDIVG

SAYYVAPEVLKRKYGKEIDIWSAGVILYILLSGVPPFWAETEKGIFDAIVEGEIDFESQP

WPSISDSAKDLVRKMLTQDPKKRITSTQALEHPWIREDGNASDKPLDNAVLSRMKQFRRM

NKLKQLALKVIAENLSTEEIQGLKQMFANIDTDNSGTITYDELKNGLARLGSKLTEAEVK

QLMEAADVDGNGTIDYIEFITATMHRHRLERDEHLYKAFQHFDKDNSGHITRDELEAAMK

EYGMGDDDTIKEIISEVDTDNDGKINYEEFRAMMRSGTQQGQLF

>GhCPK68

MGQDKAKLVEEYEILDILGRGGFSVVRKGIKRKNGSDHEKTQVAIKTLKRFGTTPSPARV

EKTIASMAALLPTRNQVSISDALLTNEILVMRKIVENVSPHPNVIDLYDVYEDQAGVHLV

LELCSGGELFDRIVAETRYSEAGAAAVVRQIAGGLAAIHKANIVHRDLKPENCLFLNKNK

DSTLKIMDFGLSSVEEFTDPVIGLFGSIDYVSPEALSQGQITAKSDMWSLGVILFILLSG

YPPFIAQSNRQKQQMIMAGEYNFDERTWKNISSSAKHLISNLLQVDPDRRPSAEQLLAHP

WVIGDSAKQEQIDAEVVSRLQSFNARRKLRAAAIASVLSSKVLLRTKRLRSLLGSHDLSK

DEIDNLKSNFKKICANGDNATLPEFEEVLKAMNMSSLLPLATRIFDLFDSNRDGTVDMRE

IVCGFSSLKNSKGDDALRLCFEMYDTDRSGCITKEELASMLRALPDDCLPPDITEPGKLD

EIFDRMDANSDGKVTFEEFKDAMQRDSSLQDVVLSSLRQQ

>GhCPK69

MGCCSSKNRLTGCSSYKSGKHSTPVQDQKVVVVSQTQVPQAKQRHHNNHQQPPSANKTSQ

VKVKDTVLGKPLEDIRQYYTLGDELGRGQFGVIYLCTENSTGHTYACKSILKRKLTSLQD

KEDIKKEVQIMQHLSGQPNIVEFKGAYEDKDCVHIVMELCAGGELFDRIIAQGHYSERAA

AAICRQVVNVVQNFHFMGVMHRDLKPENFLLSTKDEDAMLKATDFGLSVFIEQGKQYRDI

VGSAYYIAPEVLRRSYGKEIDIWSAGVILYILLCGVPPFWAETEKGIFDAILEGELSFEN

DPWPSISESAKDLVRKMLTMDPNERLTAAQVLEHPWLREGGEASDKPIDSAVLSRLKQFR

AMNQLKKLALKVIAENLSAEEIQGLKAMFKNIDTDESGSITYEELKEGLARLGSKLTEAE

VKQLMEAADVDGNGTIDYIEFISATMHRYRLERDEDLYKAFQYFDKDNSGFITMDELEAA

MKDYRMGDEASIKQIISEVDTDNDGKINYDEFCAMMRGGAPQTAKLF

>GhCPK70

MGNCCSRGSPAAEDANDDKGDAPKQAEGSPSSGGAGSNSMSDSIGNVLGRPMEDIKATYT

IGKELGRGQFGVTHLCTHKTTGEQFACKTITKRKLSTKEDIEDVKREVQIMHHLTGQPNI

VELKGAYEDKHSVHLVMELCAGGELFDRIIAKGHYSERAAASLLRTIVQIVHTCHSMGVI

HRDLKPENFLLLNNDEDSPLKATDFGLSAFYKPGEEFKDIVGSAYYIAPEVLKRKYGPEA

DIWSVGVMLYILLSGSPPFWAESENGIFNAILRGHIDFSSDPWPRISTQAKDVVRKMLNA

DPKQRLTASQVLSHPWIKEDGEAPDTPLDNAVLNRLKQFKAMNQFKKVALRVIAGCLSEE

EIMGLKEMFKGMDTDNSGTITLEELKQGLAKQGTKLSEYEVKQLMEAADADGNGTIDYDE

FITATMHMNRMDREDHLYHAFQHFDKDNSGYITTEELDQALREYGMHDDQDIKEIISEVD

IDNDGRINYDEFVAMMRKGNPEPNPKKRRELFI

>GhCPK71

MGNCCATTGPLVDTNSNKKKGKKNKAIPYSADEYGVTHGSTTFKLKVLNELTGRDISAQY

DLGREMGRGEFGVTYLCTDPNSTEKFACKSISKKKLRTAVDIEDVRREVQIMKHLPKHPN

VVTLKDTFEDDDAVHIVMELCEGGELFDRIVARGHYTERAAAGVMKTIVEVVQMCHKHGV

MHRDLKPENFLFANKKEASPLKAIDFGLSVFFKPGIAHQLLGCFSISFSILCTNIARMLF

VTGERFNEIVGSPYYMAPEVLKRNYGPEVDVWSAGVILYILICGVPPFWAETEQGVAEAI

IRSVIDFKRDPWPKVSDNAKDLVRKMLNPDPKKRLTAQEVLEHPWLQHAKKAPNVPLGET

VKARLKQFSVMNKLKKRALRVIAEHLSVEEVADIKETFDMMDTKQRGKITLEELKAGLQK

LGQQIPDADLQILVGAAGGDGDGSLNYGEFVAVSVHLRKMANDEHLHKAFAFLDLNKSGY

LEKEDLRDALNDEVDPCSEEVINAIMHDVDTNKDGRISYEEFAAMMKAGTDWRKASRQYS

RERFNSLSMKLMQVVAT

>GhCPK72

MGQCYGKVSQTQVNETATSHATTTVTEVVPADPGGQTPLQSSNGAVNYVQSVKNTPARSS

SQSPWPSPYPYGVTASPLPRGVSPSPARASRGSTPRRFFRRPFPPPSPAKHIKASLLKRL

GGKPKEGTIPEDPGTEPEQALDKSFGYGKNFGAKYELGKEIGRGHFGHTCSARGKKGDLK

DQPLAVKIISKAKMTTAISIEDVRREVKILKALSGHKHLVKFYDACEDANNVYIVMELCE

GGELLDRILARGGRYTEEDAKAILVQILSVVSFCHLQGVVHRDLKPENFLFTSGGENADM

KLIDFGLSDFIRPDERLNDIVGSAYYVAPEVLHRSYSMEADIWSIGVITYILLCGSRPFW

ARTESGIFRSVLRSDPNFDDMPWPSVSPEAKDFVKRLLNKDYRKRMTSVQALAHPWLRDD

SHPIPLDILIYRLLKSYLHASPFKRAALKALSKALTEDELVYLRAQFRLLEPNRDGSVSL

ENFKMALAQNATEAMGESRVPDILNAMGLLAYRKMYFEEFCAAAVSTHQLEAVEGWEQIA

SAAFEHFEQEGNRVISIEELARELNVGPSAYSFLKDWIRISDGKLNLLGYKKILHGVTFR

SSNTRHH

>GhCPK73

MGNCCSRGNPESLTNELGVASPENNGNVSICSRNESFSKASPTQPPASPSEEASPKPTPK

PNPIGTVLCRPMEDVRNTYTIGKELGRGQFGVTYLCTHKVTGEQFACKTIAKRKLVNKED

IEDVRREVQIMHHLTGQPNVVELKGAYEDKHSVHLVMELCAGGELFDRIIAKGHYSERAA

ASLLRIIVQIVHTCHSMGVFHRDLKPENFLLLNKEENSPLKATDFGLSLFYKPGDVFKDI

VGSAYYIAPEVLKRKYGPEADIWSIGVMLYILLCGFPPFWAESENGIFSAILRGEIDFES

DPWPAISPQAKDLVTKMLNLDPKQRLTAQQVLNHPWIKEDGEAPDIPLDNAVLARLKQFK

AMNNFKKVALRVIAGCLSEEEIQGLKEMFKTIDSDNSGTITLEELKQGLAKQGTKLTEYE

VKQLMEAADADSNGTIDYEEFITATMHMNRMDREEHLYRAFQHFDKDNSGYITIEELEQA

IREYGMHDGKDIKEIISEVDNDNDGKINYDEFVAMMRRGNPEANPKKRRDDVMFDNSD

>GhCPK74

MGHCCSKNVSVNNESTSTVNQSQPLPVPASATPSVETNSYAVSPFASPLPAGVAPSPSPA

RTPGRKFRWPLPPPSPAKPIMAAIMRRKGSNKAAPTEGTIPEDGEGAVLDKNFGYGKNLG

AKFELGKEVGRGHFGHTCWAKGKKGELKGKSVAVKIISKAKMTSAISVEDVRREVKILKA

LSCHKNMIKFHDAFEDANNVYIVMELCEGGELLDRILSRGGRYTEGDAKNIIVQILSVVA

FCHLQGVVHRDLKPENFLFTTRDEDAPMKIIDFGLSDFIRPDQRLNDIVGSAYYVAPEVL

HRSYSVEADMWSIGVITYILLCGSRPFWARTESGIFRSVLRADPNFDDSPWPSVSLEAKD

FVKRLLNKDHRKRMTAAQALAHPWLQDDNRVVPLDILIYKLVKSYIRATPFRRAAQKALS

KALPDDALLYLTGQFKLLEPKDGCVSLNNFKTALMKNRTDAMQESRVFDIINVMEPLYYK

KMDFEEFCAAAISTYQLEASEEWESIASTAFEYFEQDGNKVISVEELALELNLGPSAHSL

LKDWIRVSDGKLSFLGYTKFLHGVTIRASNVRRR

>GhCPK75

MDRVVGGKFKIGRKIGAGSFGELYLGVNTETGEEIAIKMEPAKTRHPQLHYESKLYMLLQ

GGTGIPQLKWFGVEGDYNVMVIELLGPSLEDLYNYCNRKLSLKSVLMLADQLINRVEFMH

SRGFLHRDIKPDNFLMGLGRKANQVYIIDYGLAKKYRDLQSHKHIPYRENKNLTGTARYA

SVNTHLGVEQSRRDDLESLGYVLIYFLRGSLPWQGLKAGTKKQKYDKISEKKMVTPVEVL

CQSYPSEFVSYFHYCRSLRFEDKPDYSYLKRLFRELFIREGYQFDFVYDWTILKYPQLTG

SSRQRLSSGKAALNSGASAERTDKLSGRDESNEINLQVPLRYLLGKMHQVVDILVILQNR

RYQRRHCQQKMRIMILEGDEVLPPILAACPREQLPQAAGRQPLLWGPAIDQVGFFQVVVA

SQEARDSILGLSQDHLSPKLQLRKLSVKIRIKASSTIQGLQMIESDECSIRWFWLCEEKK

METVAIGDIACLDAELLQLQEMSPLALKSYPDFTQKLFEQWLSLPATHKLVTSLINDAKA

GNPLNVPGSTSSGSTATSNTLPSMFPAGSAPPLSPRSTYGSPRIAKQRAGPSNLGSPLKV

VSEPVKELIPQFYFEKGRLPPKELKEQCISQISQFFCGHPDGLQLPEFKLVTKEICKLPT

SFSTSLFRKVDVNNTGFVTRDAFIDYWINGSMLTMDIATQIFTILKQPDLKYLTQDDFKP

LLQELLATHPGLEFLQSTPEFQERYAETVIYRIFYYINRAGNGCLTLRELKRGNLVHAML

HADEEDDINKVLRYFSYEHFYVIYCKFWELDTDHDFLIDKENLIRYSNHALTYRIVDRIF

SQVPRKFTSKVEGKMGYEDFVYFILAEEDKSSEPSLEYWLKCIDLDGNGVLTRNEMQFFY

EEQLHRMECMAQEPVFFEDILCQIMDMIKPEDDSCITLRDLKGSKLSGNAFNILFNLNKF

MAFESRDPFLIRQERENPTLTEWDRFAHREYIRLSMEEDVEDASNGSAEVWDESLEAPF

>GhCPK76

MGNCCSCGSSAEDANEKGDTITAEEAKSPNGSKQDSPSRNNTASPADSSKPSKSSPIGPV

LGRQMEDIKATYNIGKELGRGQFGVTHLCTCKQTGEQFACKTIAKRKLANKDDIEDVRRE

VQIMHHLTGQANIVELKGAYEDNHSVYLVMELCAGGELFDRIIAKGHYTERAAASLLRTI

VQIVHTCHSMGVIHRDLKPENFLLLNQDENSPLKATDFGLSVFYKPGEEFRDIVGSAYYI

APEVLKRRYGPEADIWSIGVMLYILLSGVPPFWAESENGIFNAVLRGHVDFSTAPWPSIS

AQAKDLVKKMLTVDPKQRLTAVQVLKHPWIKVDGEAPDTPLDNAVLSRLKQFKAMNQFKK

VALRVIAGCLSEEEIMGLKEMFKGMDTDNSGTITLEELRQGLAKQGTKLSEYEVKQLMEA

ADADGNGTIDYDEFITATMHMNRMDREEHLYHAFQHFDNDNSGFITTEELEQALREYGMH

DGRDLKEIISEVDIDNDGKINYDEFVAMMRKGNPKRRRDVDYIL

>GhCPK77

MGNCCATPGSPVEKNKKGQKKNKANPFYGDEYAVSNGSATTFKLRVLKELTGQDISSQYD

LGRELGRGEFGVTYLCTDVNTGEKYACKSISKKKLRTAVDIEDVRREVEIMKHLPKHTNI

VTLKDTYEDDDAVHIVMELCEGGELFDRIVARGHYTERAAAVVMRTIVEVVQMCHKHGVM

HRDLKPENFLFGNKKENAPLKAIDFGLSVFFKPGERFNEIVGSPYYMAPEVLKRNYGPEV

DVWSAGVILYILLCGVPPFWAETEQGVAQAIIRSVIDFKRDPWPKVSDNAKDLVKKMLNP

DPKQRLTAQEVLEHPWLQNAKKAPNVPLGETVKARLKQFSVMNKLKKRALRVIAEHLSVE

EVAGIKEAFDVMDTGKRGKINLEELRMGLQKLGQQIPDADLQILVEAADVDGDGTLNYGE

FVAVSVHLRKMANDEHLHKAFAFFDLNQSGFLEIEDLRDSLNDEVDTSEEVINAIMLDVD

TDKDGRISYEEFVAMMKAGTDWRKASRQYSRERFNSLSLKLMRDGSLQLGN

>GhCPK78

MGLCHGKPIENQQKQSRDISIPAEKDAAPNSNSSKSSNFPFYSPSPLPSLFKTSPAVPSV

NSTPLRFFKRPFPPPSPAKHIKSLLARRHGSIKPNEASIPEGSECEVGLNKSFGFSKHFT

SHYELGEEVGRGHFGYTCSAKAKKGSLKGNDAAVKVIPKSKMTTAIAIEDVRREVKILRA

LTGHKNLVQFYDAYEDDENVYIVMELCKGGELLDRILSRGGKYPEEDAKAVMVQILSVVA

FCHLQGVVHRDLKPENFLFTTKDEGSPLKAIDFGLSDYVKPDERLNDIVGSAYYVAPEVL

HRSYGTEADMWSIGVIAYILLCGSRPFWARTESGIFRAVLKADPSFDEAPWPSLSPDSID

FVKRLLNKDYRKRLTAAQALSHPWLANFHDIKIPSDMIICRLVKAYIGSSTLRRAALGAL

AKTLTIPQLAYLREQFSLLGPNKSGFILMQNFKTVMLKNSTDAMKDSRVLDYVNMISSIQ

YRKLDFEEFCASAVSVHQLEGMETWEQHARRAYYLFDKDGNRPIMIEELASELGLSSSVP

VHVVLQDWIRHSDGKLSFLGFVRLLHGVSSRTFQKS

>GhCPK79

MGNCCVTSAAASHENNKKKNKKKGNKKQNPFDNSNGSPKLIVLEEPTGREIEQRYELGRE

LGRGEFGITYLCTDKDTGENFACKSISKKKLRTAVDIEDVRREIKIMKHLPEHPNIVTLK

DTFEDDNAVHLVMELCEGGELFDRIVARGHYTERAAAAVAKTIVEVVQMCHKHGVMHRDL

KPENFLFENKKETAALKAIDFGLSVFFKPGERFTEIVGSPYYMAPEVLKRNYGPEVDVWS

AGVILYILLCGVPPFWAETEQGVAQAILRSVVEFKRDPWPKVSASAKDLVKKMLNPDPKR

RLTAQEVLAHPWLQNAKTAPNVSLGETVKARLKQFSVMNKLKKRALRVIAEHLTVEETAD

IKERFQEMDTGNRGKINIDELRDGLHKLGQIISDADLQVIMDAGDVNRDGYLDYGEFIAI

SIHLRKMGNDNHLKKAFEFFDKDQSSYIEIEELRDELADELETNGEEVITAIMHDVDTDK

DGRISYDEFVAMMKAGTDWRKASRQYSRQRFNNLSLKLMKDGSLLSPDNPI

>GhCPK80

MGNNCFKTISNSICGPSEEEKEKEKEEVPNVTAESESKEKGASDVQNQPPEEIKIVKEGT

EREQEGKSKEETQMQQSKCELQRLDSKPAQVSTQVTPQTAIKNEEEHKVEPKKPPRRPHN

VKRQSCAGLKIDSVLQTKTGHLKEYYSLGTKVGNGQFGTTFVCVEKGTGNKFACKSIAKR

KLATLDDVEDVRREIQIMHHMSWHPNVVTIKGAYEDPMAVHVVMDLCAGGELFDRIVKRG

HYSERKAAELARVIVGFVEACHSMGVMHRDLKPENFLFVNDEEDSPLKAIDFGLSIFFKP

GDTFSVVVGSPYYVAPEVLNKCYGPEADVWSAGVIIYILLCGVPPFWGETEEEIFDEVLN

GEPDFTSDPWPSISESAKDLLAKMLVRNPKKRITAHEVLRHSWVQADGVAPDSPLDSLVL

GRMKQFSSMNKLKKMALRVIAQRLSQEEIAGLKEMFKMIDTDNSGQITFEELKAGLQSFG

ASLPESEFQALMQAADVNNSGSIDYQEFIAATLHLNMIQNEDNVMAAFSYFDRDGSGYIT

LDELQKACQEFGIKDIRMDEMMREVDQDNDGRIDYNEFVAMMQKGNPEVGKRGREGKGLS

IGFREALPGS

>GhCPK81

MGCFSSKHKPSDPPSMPTTQPKQVQTQEVSMPQTRQPQQVQTQKVSVPEAQVPQTRQPQA

VSVPLKPSPASTRPIQTMEDTVLGKPLEDIKQYYKLGKELGRGQFGITYLCTENSTGNTY

ACKSILKRKLRSKQDREDIKKEVQIMQHLSGQPCIVEFRGAYEDRQSVHLVMELCAGGEL

FDRIIAEGHYSERAAAGIFKSVVNVVHICHFMGVIHRDLKPENFLLSSKDAGAMLKATDF

GLSVFIEEGKRYRDIVGSAYYVAPEILRRSYGKEVDIWSAGIILYILLSGVPPFWAETEK

GIFDAISEGKLDFESQPWPSISETAKDLVRKMLTKDPKKRFTSAQVLEHPWMREDGEASD

KPIGSAVLSRLKQFRAMNKLKKLALKVIAENLSEEEIKGLKVMFTNMDTDKSGTITYEEL

KTGLARLGSKLSEAEVKQLMEAADVDGNGTIDYIEFISATMNRYRLDRDELLYKAFQYFD

KDNSGYITKDELETAMKEYGMGDEASIRAVISEVDTDNDGRINYEEFCTMMRGGTQQTEK

AFLDTINSSIT

>GhCPK82

MGICLSTTKVFGTSSNPSPDHHEEKQPASSTTTTNAKKESHKPTVKHQQQLQFKAKPSSR

KQGGNVPCGKRTDFGYRKDFEKRYTTGKLLGHGQFGYTYVAIGNENGDRVAVKKIEKMKM

VLPIAVEDVKREVKILEALKGHENVVQFYNAFEDDSYVYIVMELCEGGELLDRILAKKDS

RYSEKDAAVVVRQMLKVAAECHLRGLVHRDMKPENFLFKSTKEDSPLKATDFGLSDFIRP

GKRFQDFVGSAYYVAPEVLKRKSGPESDVWSIGVITYILLCGKRPFWDKTEDGIFREVLK

NKPDFRRKPWPTISDSAKDFVKKLLVKDPRARLTAAQALSHPWVREGGDASEIPIDISVL

SNMRQFVKYSRLKQFALRALASTLNEEEIADLRDQFHAIDVDKNGVISLEEMRQALAKDL

PWKLKESRVLEILQAIDINTDGLVDFTEFIAAALHVNQMEEHDSEKWQMRSEAAFQKFDV

DRDGFITPDELRMHTGLKGSIDPLLEEADIDRDGKISLEEFRRLLRTASISVRPNPSHRT

TWKL

>GhCPK83

MGSCISTQAKLIDSLSKRYYDRSGTKGKPKHARFDHSESRKSVSKCVSSTRKVLKNQSGK

NIFDLYEIGKKLGIGEFGITHQCFDLETGETFACKKIAKAKLRTEVDLEDVRREVEIMRH

LPKHPNIVTFREAFEDKEAIYLVMELCRGGELFDRILAKGHYSEQAAATIIKTILEIVKV

CHEHGVIHRDLKPENFLFADESESAPIKAIDFGLSIFYESGQRFSDIVGSPYYMAPEVLK

RNYGEEVDVWSIGVILYILLCGVPPFWAETEEGIAHAIIKGEIDFKRDPWPKVSGEAMEL

VKSMLRPNPYNRMTIQEILEHPWIQNPKHCPNVNLGENVRSRIKQFSLMSKFKKKVLRVV

ADNLSEDQTDSIIQMFNMMDTDENGHLSFEELRDGLAKIGHSIDDPDVQMLLESADVDGS

GTLSYDEFTTMAVHLKRISDDQLSQAFQYFDKNQSGYIEVEELKEALLQDDPGPSNEQSI

KDIMLDVDEDKAKPH

>GhCPK84

MGNTCVGPSISKNGFFQSVSAAMWRSRMPDDSVSSSNGGTGSEVAAREPESPLPVLNKPP

EQVTIPKPEAKQETKPETKPETEPEKPAKPKFPHMKRVSSAGLRTESVLQTRTGNFKEYF

SLGRKLGQGQFGTTFLCVEKSTGREYACKSIAKRKLLTDEDVEDVRREIQIMHHLAGHPN

VISIKGAYEDAVAVHVVMELCAGGELFDRIIQRGHYTERKAAALTRTIVGVVEACHSLGV

MHRDLKPENFLFVNQQEDALLKAIDFGLSIFFKPGSERFTDVVGSPYYVAPEVLRKHYGP

EADVWSAGVILYILLSGVPPFWAENEQGIFEEVLHGDLDFDSDPWPSISESAKDLVRKML

IRDPRKRLTAHEVLCHPWIQIDGVAPDKPLDSAVLSRMKQFSAMNKLKKIALRVIAESLS

EEEIAGLKEMFKMIDADNSGQITFEELEVGLKRVGANLKESEIYDLMQAADVDNSGTIDY

GEFVAATLHLNKIEKEDHLFAAFSYFDKDGSGYITPDELQKACEEFGIEDVRLEEMIREV

DQDNDGRIDYNEFVAMMQGGNVAGAGKKGLQNSSSIKFREALKF

>GhCPK85

MGNCNRPPIASNRFRPEPDPGGTHHNDDNVKPVPGYSPPPKTHYYHSSTIASKNRLATPN

TPPIGRVFGRTMEDVRSTYVVGRELGRGQFGVTYLVTHKETKQHFACKSISRRKLLTRDD

IEDVRREVQIMYHLTGHRNIVELETACEDRRSVNLIMELCAGGELLDRILAKGHYSEKEA

ANLCRQIVTVIHNCHSMGVMHRDLKPENFLFLSKDEDSPLKATDFGLSTFFKPGEVFKDF

VGTAFYMAPEVLHWRYGPEADIWSAGVILYVLLSGSPPFYGETEKSIFKSILQGNINFLS

DPWPSVSDSAKDLLSKMLRQDPKERLSASEVLDHLWLREDGDASDKPLDVAVLTRMKQFR

AMNKFKKVALKVIAENLSEEEIVGLKEMFKLMDTDNSRTITFDELKAGLCKLGTNVSESE

VRLLMEATDIDGNGTIDYVEFITATMHMNRMEKEKRLYTAFQHFDKDNSGYITMEELKQA

LQKYNTRDEKTINEILAEVDTDKDGRINYDEFVAMMRKGNPELVGNRLHT

>GhCPK86

MGNCNACVRPDESPESKPNRSDQNQKGKKKARERKPNPFSEKVASPAPIRVLKDVIPLSH

RTRIGDKYILGRELGRGEFGITYLCTDRETREALACKSISKRKLRTAVDIEDVRREVAIM

STLPEHPNIVKLKATYEDNENVHLVMELCEGGELFDRIVARGHYTERAAANVAKTIAEVV

RMCHENGVMHRDLKPENFLFANKKEHAPLRVIDFGLSVFFKPGEKFSEIVGSPYYMAPEV

LKRNYGPEVDVWSAGVILYILLCGVPPFWAETEQGVALAILRGVIDFKREPWPQVSESAK

SLVRQMLEPDPRKRLTAQQVLEHPWLQNTKKAPNVPLGDIVRARLKQFSVMNRFKKKALR

VIAEHLSVEEVEVIKDMFALMDTDNDGKVSYEELKAGLRKVGSQLAEPEIKMLMEVADVD

GNGVLDYGEFVAVTIHLQKMENDEHFRRAFMFFDKDGSGYIELDELRDALADESGETDVD

ALNDIMREVDTDKDGQICYDEFVAMMKAGTDWRKASRQYSRERFKSLSLNLMKDGSLQLH

DAVTGQAVAV

>GhCPK87

MGQCYGKVNQSGVHEGTTTTMVVSADRDETTAQSANGAGNVPSVKNTPARSSSQSPWPSP

YPHGVSASPLPPGVSPSPARASRGSTPRRFFRRPFPPPSPAKHIKASLVKRLGGKPKEGP

IPEDRGTEPEQALDKNFGYGKNFGAKYELGKEVGRGHFGHTCSARGKKGELKDQPVAVKI

ISKAKMTTAISIEDVRREVKILKALSGHKHLVKFYDACEDANNVYIVMELCEGGELLDRI

LARGGRYTEDDAKAIVVQILSVVSFCHLQGVVHRDLKPENFLFTSGGEDADMKLIDFGLS

DFIRPDGRLNDIVGSAYYVAPEVLHRSYSLEADIWSIGVITYILLCGSRPFWARTESGIF

RSVLRSDPNFDDLPWPSVTPEAKDFVKRLLNKDYRKRMTAVQALTHPWLRDDSRPVPLDI

LVYKLVKSYLHATPFKRAALKALSKALTEDELVYLRAQFRLLEPSRDGSVSLENFKMALA

RNATEAMGESRVPDILNTMGTLAYRKMYFEEFCAAAISTHQLEAVEGWEQIASTAFEHFE

QEGNRVISIEELARELNVGPSAYSFLKDWIRVSDGKLSLLGYTKFLHGVALRSSNTRHH

>GhCPK88

MGACLSTTKVIGSSSNAAAHHRKHQPSATTVTVNEKKESRKPNNQQGQRQQVRSSQPLKV

KGKPSSTRQTGIIPCGKRTDFGYHKDFDQRYTIGKLLGHGQFGYTYVAIDKANGDRVAVK

KIDKNKMVLPMAVEDVKREVKILEALKGHENVVQFYNAFEDDSYVYIVMELCEGGELLDR

ILAKKDSRYSEKDAAVVVRQMLKVAAECHLHGLVHRDMKPENFLFKSTREDSLLKATDFG

LSDFIRPGKRFHDIVGSAYYVAPEVLKRRSGPESDVWSIGVITYILLCGKRPFWDKTEDG

IFKEVLKKKPDFRRKPWPTISNGAKDFVKKLLVKDPHARLTAAQALSHRWVREGGNASDI

PVDISVLNNLRQFVKYSRLKQFALRALASTLNEEEIADLRDQFDAIDVDKNGSISLEEMR

QALAKDLPWKMKEPRVLEILQAIDSNTDGLVDFKEFVAAALHVNQMEEHDSDKWQMLSQA

AFEKFDVDRDGFITPEELRMHTGLRGSIDPLLEEADIDKDGKISLSEFRRLLRTASISSR

NVPCSSDQRNSRKL

>GhCPK89

MGLCTSKPSPNPSFPPDSRNTPIHPRNKDIHHNSNSVPASPLPDVVNGNRNHNLDQAKGT

EEKESSNSNNEGKKSPFFPFYSPSPAHYLFSKKSPARSSANSTPKRFFRKPFPPPSPAKH

IRAVLARRHGSVKPNESAIPEGTEADGDGAAGATGAGLDKSFGFSKHFGSKYELGEEVGR

GHFGYTCAAKFKKGELKGQQVAVKVIPKAKMTTAIAIEDVRREVKILRALSGHNNLVQFY

DAYEDHDNVYIVMELCEGGELLDRILSRGGKYTEDDAKDVMIQILNVVSFCHLQGVVHRD

LKPENFLFTSKDENSQLKAIDFGLSDFVKPDERLNDIVGSAYYVAPEVLHRSYSTEADVW

STGVIAYILLCGSRPFWARTESGIFRAVLKADPSFDEAPWPALSSEARDFVKRLLNKDPR

KRLTAAQALSHPWIKNYNDVKVPLDILIFKLLKAYLRSSSLRKAALRALSKTLTVDELFY

LKEQFALLEPNKNGTISLENIKVALMKNATDAMKESRIPEFLASLSALQYRRMDFDEFCA

AALSVHQLEALDRWEQHARCAYELFEKDGNRAIVIDELASELGLSPSVPVHAVLHDWIRH

TDGKLSFLGFIKLLHGVSSRTFAKAQ

>GhCPK90

MGNCCRSPAAVAREDVKSNFSGRDHARKDSVSKQKPAITVLAGVPKDSIEEKYLVDRELG

RGEFGVTYLCIDRGTRELLACKSISKRKLRTAVDIDDVRREVAIMKHLPKDSSIVSFKEA

CEDDNAVHLVMELCEGGELFDRIVARGHYTERAAAAVTRTIMEVVQLCHKHGVIHRDLKP

ENFLFANKKEKSPLKAIDFGLSIFFKPGERFSEIVGSPYYMAPEVLKRNYGPEIDIWSAG

VILYILLCGVPPFWAESEQGVAQAILRGLIDFNHDPWPNISESAKSLVQQMLEPDPKLRL

TAKQVLEHPWLQNAKKAPNVPLGDVVKSRLKQFSIMNRFKRKALRVIAEFLSVEEVEGIK

EMFKKMDTDNDGIVSIEELKAVFGNVGSQLAESEVQMMLIEAIDANGKGTLDYGEFLAVS

LHVQRMANDEHLRKAFSYFDRDGNGFIEPDELRDALMEGADDCTSVANDIFQEVDTDKDG

RITYDEFAAMMKTGTDWRKASRHYSRGRFNSLSIKLMKDGSLNLGRE

>GhCPK91

MRLHYCMRLHYYIFLMGNTCRGSGQLHQGFSEPEERSSKQSYASSDHSTSDYSRSGLNDQ

QLAPQVIPKSTTKDANLVTTVPPKKENSMKRGFDHHVCHVLGHKTPNIRDLYTIGRKLGQ

GQFGTTYLCTEISTSFEYACKSICKRKLLSKEDVEDVRREIQIMHHLAGHKNIVAIKGAY

EDTLYVHIVMELCSGGELFDRIIERGHYTERKAAALTKIIVGVVEACHSLGVMHRDLKPE

NFLLVNKDDDFSLKAIDFGLSVFFKPGQIFTDVVGSPYYVAPEVLLKHYGPEADVWTAGV

ILYILLSGVPPFWAETQQGIFDAVLKGNIDFDSDPWPVISDSAKDLIRKMLCSQPSERLT

AHEVVCHPWISQNGVAPDRALDPAVLSRLKQFSAMNKLKKMALRVIAESLSEEEIAGLRE

MFTAMDTDNSGAITFDELKAGLRRYGSTLKDTEIRDLMNAADVDNSGSIDYGEFIAATVH

LNKLEREEHLVAAFKYFDKDNSGYITVDELQQACAEHNITDVLLEDIIREVDQDNDGRID

YGEFVAMMQKDNSGIGRRTMRNSLNLSMRDPVGAQ

>GhCPK92

MGLCTSKPSPNPSDSTNASINTRNNDIYRKPNSVSASPLPDGVNSKEDQGKQGEEEKESS

NPNNEGKKSPFFPFYSPSPAHYLFSKKSPARSSTNSTPKRFFRRPFPPPSPAKHIRAVLA

RRHGSVKPNEAAIPEGSDAEAAGATGTGLDKSFGFSKHFGSKYELGDEVGRGHFGYTCTA

KFKKGELKGQQVAVKVIPKAKMTTAIAIEDVRREVKILRALSGHSNLVQFYDAYEDHDNV

YIVMELCEGGELLDRILSRGGKYTEDDAKAVMIQILNVVAFCHLQGVVHRDLKPENFLFT

SKDENSQLKAIDFGLSDFVKPDERLNDIVGSAYYVAPEVLHRSYSTEADVWSIGVIAYIL

LCGSRPFWARTESGIFGAVLKADPSFDEAPWPSLSSEARDFVKRLLNKDPRKRLTAAQAL

SHPWIKKYNDVKVPLDILIFKLVKAYLRSSSLRKAALRALSKTLTVDELFYLKEQFALLE

PNKNGTISLENIKAALMKNATDAMKDARIPEFLASLNALQYRRMDFDEFCAAALTVHQLE

ALDRWEQHARCAYEIFEKEGNRPIVIEELASELGLSPSVPVHAVLHDWIRHTDGKLSFLG

FVKLLHGVSS

>GhCPK93

MGNCNSQPSADSQFRPETNTGGGPNHGINVLPADASAPRPPKPSTHHNLSSSSAAPNHHT

APSATPPIGRVLGRPMEDVRSTYVFGRELGRGQFGVTYLVTHKETNQQFACKSIATRKLI

NRDDIEDVRREVQIMYHLTGHRNIVELKGAYEDRHSVNLIMELCAGGELFDRIIAKGHYS

EREAANLCRQIVTVVHNCHSMGVMHRDLKPENFLLLSKDEDSPLKATDFGLSVFFKPGDI

FKDLVGSAYYVAPEVLRRRYGHEADIWSAGVILYILLSGVPPFYGETEQSIFDSILRGNI

DFASDPWPFVSNSAKDLVRKMLRHDPKDRLSAVEVLNHPWIREDGDASDKPLDVAVLTRM

KQFRAMNKLKKVALKVIAENLSEEEIIGLKEMFKSMDTDNSGTITFEELKAGLPKLGTKL

SEHEVRQLMEAADVDGNGAIDYIEFITATMHMNRMEREDHLYTAFQYFDKDNSGYITMEE

LEQALKKYNMGDEKTIKEIIAEVDTDRDGRINYDEFVAMMRKGNPELANNRRRK

>GhCPK94

MGNTCVGPSISKNGFFQSVSAVMWPNRSPEGSVSHRETGNEVASKEPESALPVQSRPPEQ

ITMPKSETKQETKSKKPKKPKPPHVKRVSSAGLRTESVLQTKTGNFKEYYSLGKKLGQGQ

FGTTFLCVEKSTGKEYACKSIAKRKLLTDEDVEDVRREIQIMHHLAGHPNVISIKGAYED

AMAVHVVMELCAGGELFDRIIQRGHYTERKAAALTRTIVGVVESCHSLGVMHRDLKPENF

LFINQQEDSLLKTIDFGLSMFFKPGETFMDVVGSPYYVAPEVLRKHYGPEADVWSAGVIL

YILLSGVPPFWAESEDGIFDQVLHGDLDFESDPWPNISESAKDLVRRMLIRDPKKRLTAH

AVLCHPWIQVDGVAPNKPLDFAVLSRLKQFSAMNKLKKMVLRVIAENLSEEEIAGLKEMF

KMIDTDNSGQITFEELKAGLKRVGANLKESEIYDLMQARHNDYGEFVAATLHLNKIERED

HLFAAFSYFDKDGSGYITPDELQQACEEFGIEDVRLEEMIHEVDQDNDGRIDYNEFVAMM

QKGHVATNAGSAGAGKEGLQHSFGIGFREALKL

>GhCPK95

MGNTCLGSFKGNNHFQGYNTTTGANLAPSISPTANTMRRAFDHQAHFVLGRKTPNIHELY

TFGRKLGQGQFGITYLCTEISTGIEYACKSISKRKLIRKEDVEDARREIQIMHHLAGHKN

IVSIKGAYEDTLYVYIVMEFCSGGELFDRIIQRGHYSERKAAELTKIIVGVIEVCHSLGV

MHRDLKPENFLLVNKDDDFSLKAIDFGLSAFFKPGEIFSDVVGSPYYVAPEVLLKHYGPE

ADIWSAGVILYILLCGLPPFWAETIQEIFAAVLKGHIDFSSYPWPLISDSAKDLIRKMLC

IRPSERLTAHEVLCHPWICKNGVAPDKALDPAILSRLKQFSAMNKLKKMALRVIAESLSE

EEIAGLREIFTAMDTGKSGAITFDELKAGLQRYGATLKDTEIQDLMNAADVSNSGTIDYG

EFIAATIHLNKLEREEHLVAAFQYFDKDKSGYITIGELKQVCAELNVTNVLLEDIIQEVD

QDNDGRIDYAEFVAMMQKGNAGVGRRPIRNSLNMSIRDVPGSQ

>GhCPK96

MRRAIDHQAQYVLGHKTPNILELYTLGRKLGQGQFGVTYLCTEILTGIEYACKSISKRKL

ICKEDVEDVRREIQIMHHLASHKNIVTIKGAYEDPLFVHIVMELCSGGELFDRIIQRGHY

TERKAAELTKIIVGVVEACHSLGVMHRDLKPENFLLVNKDDDFSLKAIDFGLSVFFKPGE

VFTDVVGSPYYVAPEVLLKCYGPEADVWTAGVILYILLSGVPPFWAETQQGIFDAVLKGY

VDFDSDPWPLISDSAKDLIQKMLCSQPSERLTAHEVLCHPWICENGVAPDKALDPAVLSR

LKQFSAMNKLKKMALRVIAESLSEEEIAGLREMFTAMDTDNSGAITFDELKAGLRSYGST

LKDTEIRDLMDAADVDNSGTIDYGEFIAATVHLNKLEREEHLVAAFRYFDKDNSGYITVD

ELQQACTEHNMTDVLLEDIIREVDQDNDGRIDYGEFVAMMQKGNAGVGRRTMRNSLNMSM

RDVPAAL

>GhCPK97

MGGCLTKNKDPMPQHNGYRSGATTTAAVHQQRYQDPVRPAPIQPQVYHIPQKPGTQTPWK

PAVPAPSPKPAPNIDTILGKPFEDIRMHYTIGKELGKGQFGVTYHCTENSTGKQYACKTI

SKRKLVRKNDKEDMKREIQIMQHLSGQPNIVEFKGAYEDKQSVHVVMELCAGGELFDRII

AKGHYSERAAASICRAIVNVVHACHFMGVMHRDLKPENFLLSSKGENALLKATDFGLSVF

IEEGKVYQDIVGSAYYVAPEVLLRKYGKEIDIWSAGVILYILLSGVPPFWAETEKGIFDA

ILEGEIDFESQPWPSLSDSAKDLVCRMLTQDPKKRITSTQVLEHPWIREGGNASDKPLDS

AVLSRMKQFRRMNKLKQLALKVIAENLSREEIQGLKQMFANIDTDNSGTITYDELKTGLA

RLGSKLTETEVQQLMEAADVDGNGTIDYIEFITATMHRHRLERDEDLYKAFQHFDKDNSG

HITRDELEAAMKEYGMGDDDTIKEIISEVDTDNDGKINYEEFRDMMRSGTQHGQLF

>GhCPK98

MGNCCTRGDGSDKLEKAAAGYGNGDADPTVTSHQTSYRTAPSSQGASTVGKQSKPAPMGP

VLGRPMEDVKATYTIGKELGRGQFGITHLCTHKTTGEQFACKTIAKRKLSSKEDVEDVRR

EVQIMHHLTGQPNIVELKGAYEDKHAVHLKITYSVLAWFKTDEIFKDIVGSAYYIAPEVL

KRKYGPEADIWSVGVMLYILLSGVPPFWAESENGIFNAIIKSHVDFSGKPWPSISHQAKD

LVKRMLNPDPKRRLTAAQVLSHPWIKEDGEAPDTPLDNAVLSRLKQFKAMNQFKKVALKV

IAGCLSEEEIRGLKEMFKAMDTDNSGTITLEELRQGLAKQGTKLSEYEVKQLMEAADADG

NGTIDYDEFITATMHMNRMDREEHLYHAFQHFDKDNSGYITTEELEQALREYGINDSTDI

KQILSEVDADNDGRINYDEFVAMMKKGNPEPNPKKRRDVVV
