# Supplementary figures and images for: Calcium-dependent protein kinases in cotton: insights into early plant responses to salt stress
Source: BMC Plant Biol. 2018 Jan 17;18:15. doi: 10.1186/s12870-018-1230-8 (PMC5772696; doi:10.1186/s12870-018-1230-8)

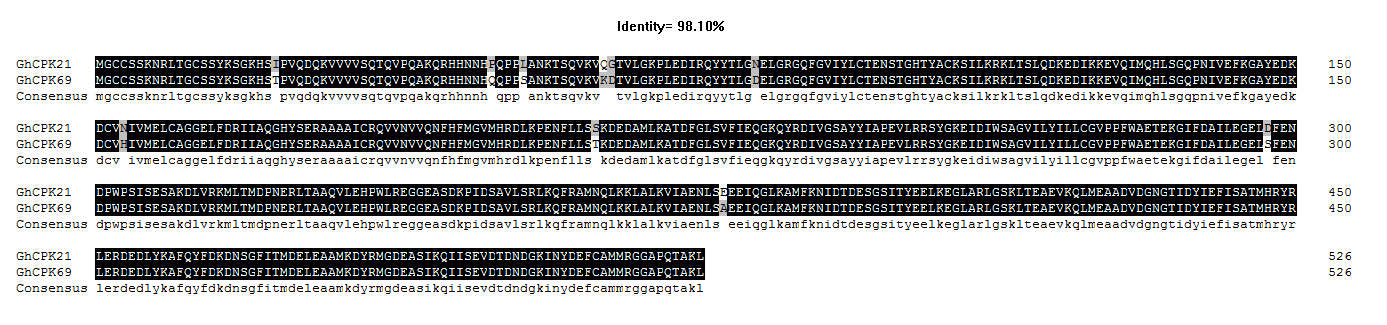

Supplement: Supplementary file 3 — Sequence alignments showing the homologies GhCPK21 and GhCPK69. The alignments were performed using DNAMAN software. Identical amino acids are indicated with black highlighting. (TIFF 96 kb) [file 12870_2018_1230_MOESM3_ESM.tif]

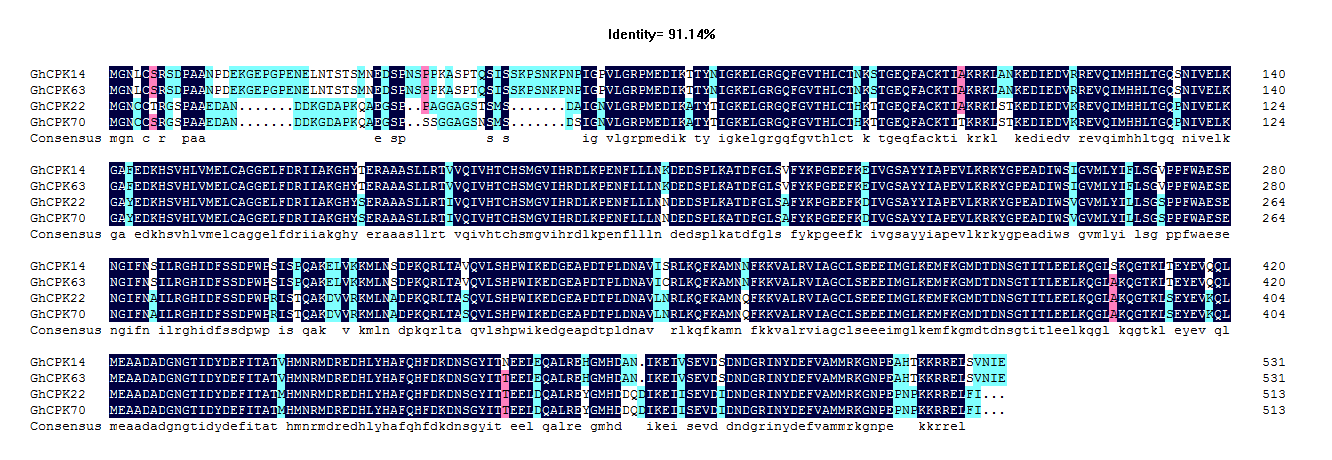

Supplement: Supplementary file 4 — The multiple sequence alignments showing the homologies of GhCPK14, GhCPK22, GhCPK63, and GhCPK70. The alignments were performed using DNAMAN software. Identical amino acids are shown with dark blue highlighting. (TIFF 192 kb) [file 12870_2018_1230_MOESM4_ESM.tif]

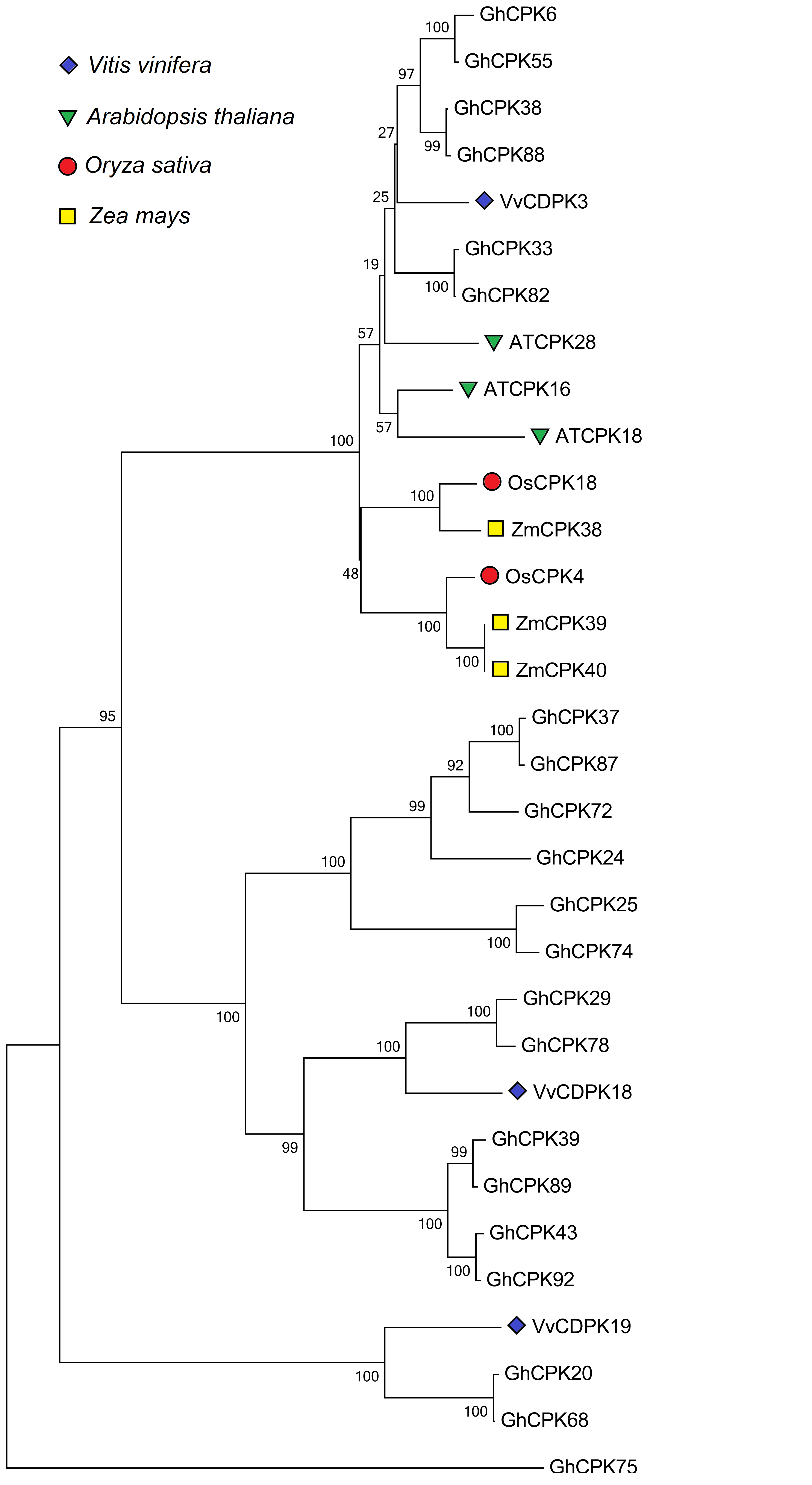

Supplement: Supplementary file 5 — The phylogenetic tree of group IV CPKs. The un-rooted tree was generated using MEGA6.0 with the neighbor-joining method (1000 bootstrap replicates). CPKs from different species are denoted with differently colored rhombuses. (TIFF 2051 kb) [file 12870_2018_1230_MOESM5_ESM.tif]

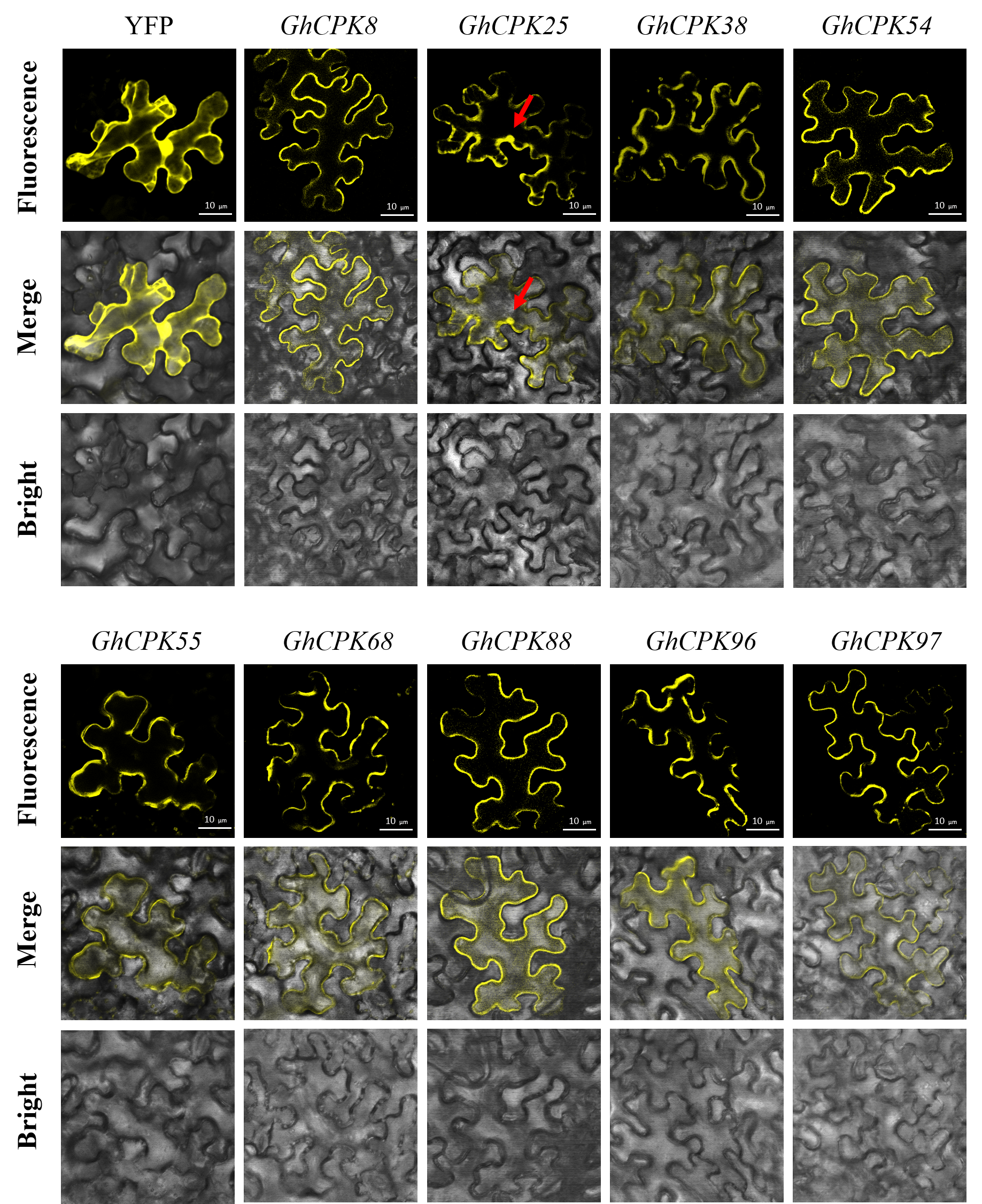

Supplement: Supplementary file 9 — Subcellular localization of GhCPKs without FM4–64 staining. The YFP observed in cell nuclei were marked with red arrows (scale bar = 10 μm). (PNG 3100 kb) [file 12870_2018_1230_MOESM9_ESM.png]
